# Smart Bioinks for 4D Bioprinting: Requirements, Design, and Applications

**DOI:** 10.1002/advs.77791

**Published:** 2026-09-27

**Authors:** Shangsi Chen, Jiahui Lai, Qiongjiao Zeng, Liangbin Zhou, Boguang Yang, Bin Zhang, Min Wang, Jiajing Zhou, Kieran Lau, Khoon S. Lim, Zhong Alan Li, Rocky S. Tuan

**Affiliations:** ^1^ Department of Biomedical Engineering The Chinese University of Hong Kong (CUHK) Shatin Hong Kong China; ^2^ InnoHK Center for Neuromusculoskeletal Restorative Medicine Shatin Hong Kong China; ^3^ Department of Mechanical and Aerospace Engineering Brunel University London London UK; ^4^ Department of Mechanical Engineering The University of Hong Kong Hong Kong China; ^5^ College of Biomass Science and Engineering Key Laboratory of Leather Chemistry and Engineering of Ministry of Education National Engineering Laboratory for Clean Technology of Leather Manufacture Sichuan University Chengdu China; ^6^ Charles Perkins Centre and School of Medical Sciences The University of Sydney Camperdown New South Wales Australia; ^7^ Institute for Tissue Engineering and Regenerative Medicine The Chinese University of Hong Kong (CUHK) Shatin Hong Kong China; ^8^ Peter Hung Pain Research Institute The Chinese University of Hong Kong (CUHK) Shatin Hong Kong China; ^9^ Shun Hing Institute of Advanced Engineering The Chinese University of Hong Kong (CUHK) Shatin Hong Kong China; ^10^ Key Laboratory of Regenerative Medicine Ministry of Education The Chinese University of Hong Kong (CUHK) Shatin Hong Kong China; ^11^ School of Biomedical Sciences The Chinese University of Hong Kong (CUHK) Shatin Hong Kong China

**Keywords:** 4D bioprinting, artificial intelligence, hydrogels, machine learning, smart bioinks, stimuli‐responsive

## Abstract

3D bioprinting is known for its high precision and reproducibility in fabricating complex and customized biomedical constructs. However, its applications are limited by their static nature; i.e., unlike native tissues, they cannot change shape or functionality over time. To overcome this, 4D bioprinting has emerged as a groundbreaking strategy by incorporating time as the fourth dimension, enabling dynamic structures that adapt in response to stimuli, thereby more accurately replicating living tissues. The success of 4D bioprinting hinges on the development of advanced smart bioinks, as their physicochemical properties uniquely dictate the shape‐morphing behavior, functionality, and performance of bioprinted constructs. These bioinks must be precisely engineered to respond to specific stimuli. This review first introduces 4D bioprinting technologies for tissue engineering scaffolds. We then outline essential requirements for smart bioinks and highlight how AI, particularly machine learning, is revolutionizing their design. Additionally, we examine widely used biomaterials for 4D bioprinting and discuss promising candidates for 4D printing. We also present cutting‐edge bioink applications in tissue engineering, drug screening, and disease modeling, showcasing their potential in regenerative medicine and personalized therapeutics. Finally, we discuss current challenges and future perspectives, underscoring the transformative impact of smart bioinks and 4D bioprinting on biomedical innovation.

## Graphical abstract

Smart bioinks empower 4D‐bioprinted constructs to dynamically adapt and remodel in response to stimuli, effectively biomimicking native tissues. Artificial Intelligence (AI) and Machine Learning (ML) play a guiding role in their rational design by optimizing relevant properties. Once the shape‐morphing process is completed, these constructs have extensive applications in tissue engineering, drug screening, and disease modeling, advancing regenerative medicine and personalized therapeutics.

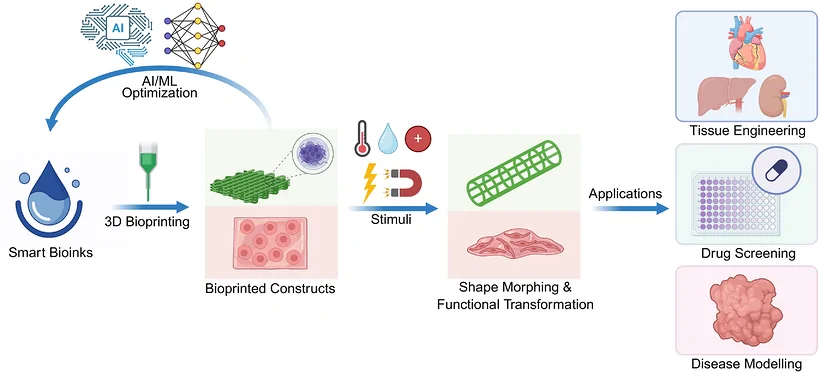

## 1 Introduction

**FIGURE 1:**
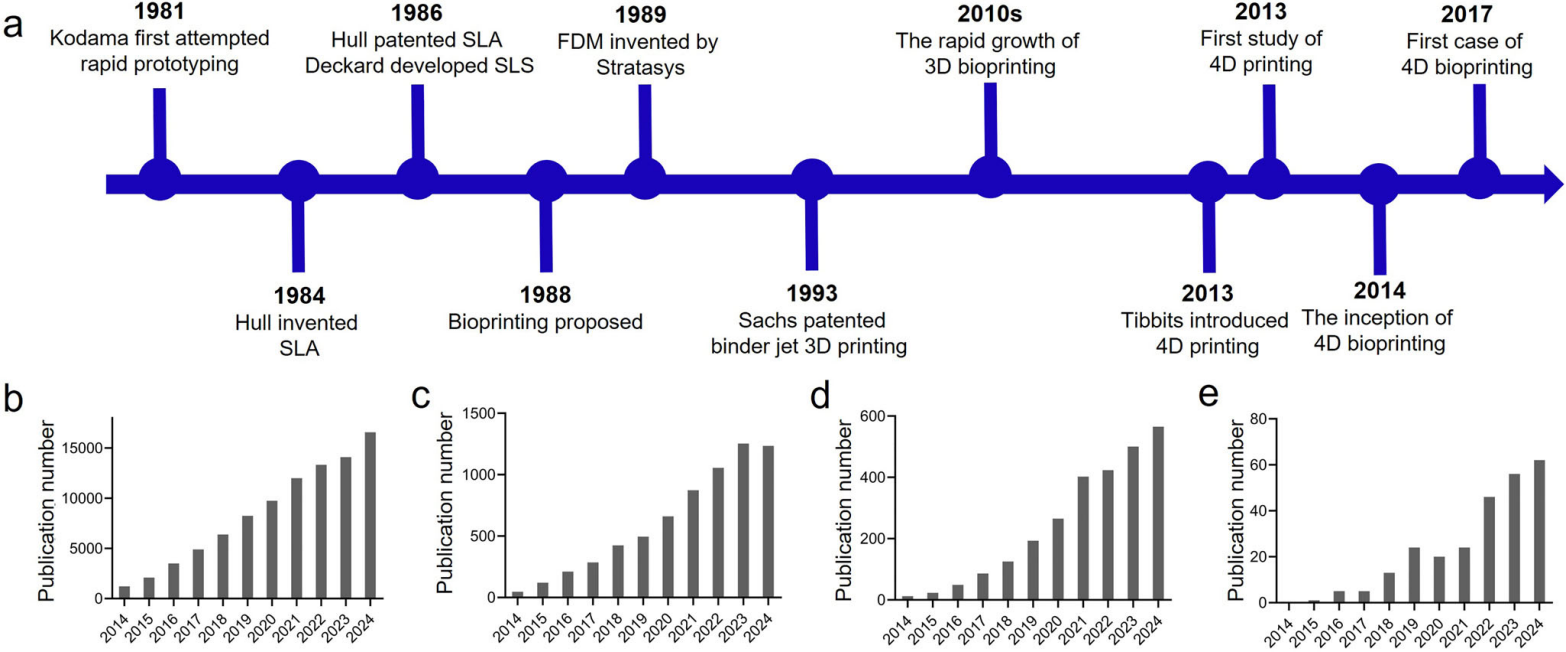
Development of 3D/4D printing/bioprinting technologies. (a) A brief timeline showing the major milestones in the development of 3D/4D printing/bioprinting technologies. (b‐e) Number of publications related to (b) 3D printing, (c) 3D bioprinting, (d) 4D printing, and (e) 4D bioprinting in the past decade. The publication data statistics were collected from the Web of Science database on Mar 18, 2026.

Many fabrication methods, including solvent casting/particulate leaching, freeze‐drying, gas foaming, thermally induced phase separation (TIPS), and electrospinning, have been extensively used to construct 3D structures, such as scaffolds for biomedical applications [1, 2]. However, these fabrication methods are unable to precisely control the microstructure (e.g., pore size, pore shape, geometry, porosity, and interconnectivity) of resulting 3D objects. In this context, 3D printing technologies have emerged and flourished in recent years (Figure 1a). 3D printing controls the micro/macrostructures of scaffolds through layer‐by‐layer positioning of materials, biomolecules, and even living cells, holding unprecedented potential in engineering complex customized biomedical products with high accuracy and reproducibility (Figure 1b) (Supporting information) [3, 4, 5, 6].

The concept of 3D printing can be traced back to the early 1980s (Figure 1a). In 1981, Dr. Kodama of Japan tried to develop a rapid prototyping system with a layer‐by‐layer approach for manufacturing, specifically using ultraviolet (UV) light to polymerize photosensitive resins. This was the prototype of stereolithography (SLA). Unfortunately, he was unable to meet the requirements to patent this technology. In 1984, Chuck Hull proposed SLA and two years later, patented the first 3D printer for SLA in the U.S., marking the advent of 3D printing technology [7]. Also in 1986, Deckard developed another 3D printing technology, selective laser sintering (SLS), which uses a laser beam to sinter solid powders layer‐by‐layer to form 3D structures, at the University of Texas at Austin [8]. The concept of bioprinting (the use of computer‐aided transfer processes for patterning and assembling living and non‐living materials with a prescribed 2D or 3D organization in order to produce bioengineered structures serving in regenerative medicine, pharmacokinetic and basic cell biology studies [9]) was first introduced in 1988, when Klebe used a standard Hewlett‐Packard (HP) inkjet printer to deposit cells for the construction of 3D synthetic tissues [10]. The 21st century has witnessed the flourishing and rapid development of 3D bioprinting (Figure 1c) [11, 12, 13].

**FIGURE 2:**
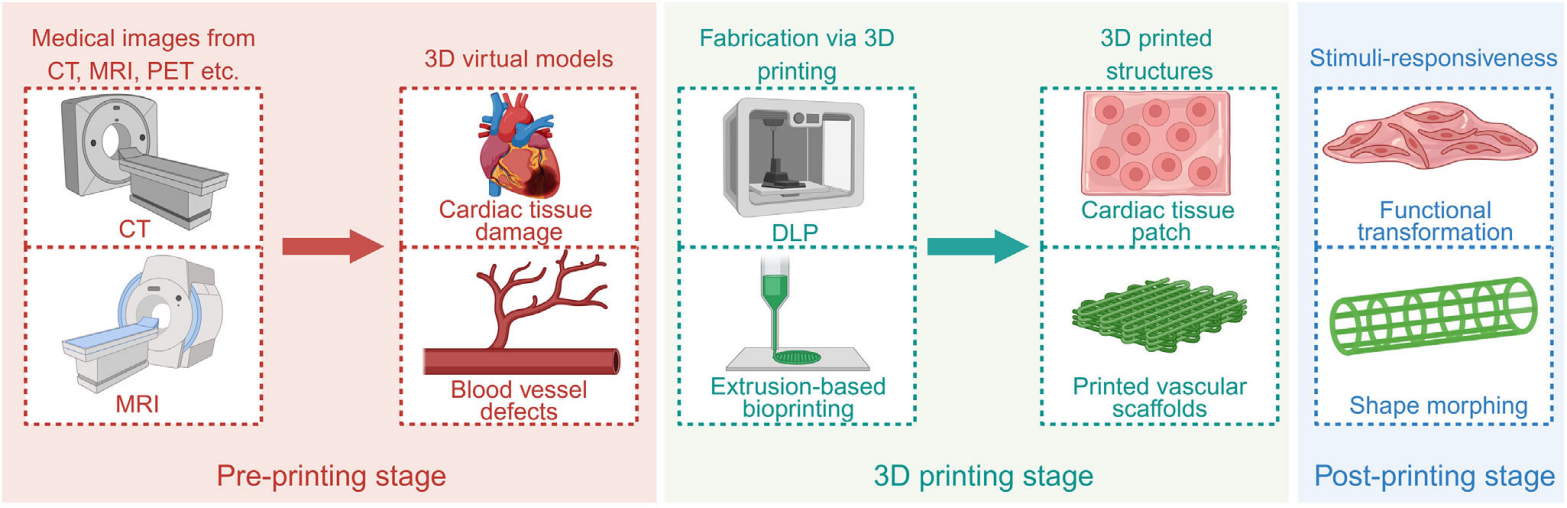
A typical 4D bioprinting process. CT, computed tomography. MRI, magnetic resonance imaging. PET, positron emission tomography. DLP, digital light processing. Created with BioRender.com.

In addition to their complex 3D hierarchical structures, native tissues and organs also exhibit dynamic properties, such as conformational changes and functional transformations, that enable their unique biological functions. This has inspired the development of 4D printing by introducing time as the fourth dimension [14]. The concept of 4D printing was first introduced in 2013 when Tibbits presented it in a TED talk. At that time, it was simply defined as “3D printing + time,” allowing the creation of 4D objects capable of changing shape over time [15]. Later, the first 4D printing study was published by H. Jerry Qi and colleagues in 2013 [16]. Currently, 4D printing refers to an advanced form of bioprinting where printed objects can alter their shape and/or properties over time in response to appropriate external or internal stimuli (e.g., temperature, pH, humidity, light, etc.) (Figure 1d) [17, 18, 19]. A key distinguishing characteristic of 4D printing is programmability, which refers to the capacity to accurately pre‐design and control the transformation pathway, kinetics, and final configuration via the rational design of material composition, spatial patterning, and printing parameters. This programmed responsiveness allows for the fabrication of dynamic structures capable of autonomously adapting to their environment, setting 4D printing apart from conventional static 3D printing. A typical 4D printing/bioprinting process is illustrated in Figure 2. Generally, a 4D printing/bioprinting process can be divided into three stages: pre‐printing stage, 3D printing stage, and post‐printing stage.

Generally, 4D printed structures can be fabricated using 3D printing techniques but require specialized materials that are sensitive to external or internal stimuli to enable the printed structures to change their shapes and/or functions in response to specific stimuli. Although the promise of “4D bioprinting” was enthusiastically illustrated shortly after the concept of 4D printing was coined in 2013 [20, 21], the first 4D bioprinted hydrogel structure was not reported until 2017 by Kirillova and colleagues [22]. They carried out extrusion‐based 3D bioprinting of mouse bone marrow stem cell (BMSC)‐laden alginate (Alg)/hyaluronic acid (HA) bioinks to obtain hydrogels that underwent shape change, i.e., self‐folding, in response to hydration/dehydration cycles. This study advanced 4D printing to 4D bioprinting by incorporating cells into dynamic hydrogel structures. Following this study, an increasing number of studies have used 4D bioprinting technologies to fabricate dynamic cell‐laden hydrogels for various applications, such as tissue engineering, drug delivery, and disease modeling [23, 24, 25, 26].

It is worth noting that the terms “printing” and “bioprinting” have often been used interchangeably in numerous publications, leading to potential misconceptions regarding the distinct emphasis and unique advantages of each technique [27, 28]. 3D/4D printing and 3D/4D bioprinting are both additive manufacturing techniques that construct a 3D structure layer‐by‐layer. In a broad sense, 3D/4D bioprinting involves the utilization of cell‐laden bioinks or inks incorporating other biologics (e.g., exosomes, growth factors, RNA, DNA, etc.) to fabricate intricate 3D/4D structures, whereas 3D/4D printing does not incorporate cells or biologics. However, in a strict sense, 3D/4D bioprinting employs cell‐laden bioinks to fabricate intricate 3D/4D architectures [29], while 3D/4D printing utilizes material‐based, cell‐free inks. In this context, the utilization of cell‐laden bioinks is a defining characteristic of bioprinting.

The incorporation of cells in 3D‐printed structures bestows significant advantages upon 3D/4D bioprinting over 3D/4D printing. Previous studies have demonstrated that using cell‐laden bioinks allows for significantly higher cell densities (>1 × 10^6^ cells/mL) in 3D/4D bioprinted constructs as compared to seeding cells onto 3D/4D printed structures [30]. Moreover, many studies have revealed that 3D/4D bioprinted structures carrying abundant exogenous or autologous cells could protect the cells and promote their bioavailability and significantly accelerate tissue remodeling after implantation [31, 32]. When a sufficient number of cells are introduced into a tissue defect, these cells could secrete a variety of biomolecules (e.g., growth factors, cytokines, and extracellular vesicles) through paracrine signaling [33]. These biomolecules can promote cell proliferation, microvessel formation, extracellular matrix (ECM) synthesis, and immune modulation, thereby establishing a conducive environment for tissue regeneration [34, 35]. The secretion of matrix proteins can facilitate ECM remodeling, which is beneficial for tissue organization and structural integrity. The immunomodulatory properties of the cellular secretome can balance anti‐inflammatory and pro‐inflammatory responses, thereby facilitating tissue healing. Furthermore, stem cells in 3D/4D bioprinted constructs can differentiate into specific cell lineages essential for tissue regeneration, such as osteoblasts facilitating bone repair, chondrocytes promoting cartilage restoration, and endothelial cells for improving blood vessel formation [11, 36]. Therefore, because of the benefits of encapsulated exogenous or autogenous cells, 3D/4D bioprinting is generally preferred to 3D/4D printing in tissue engineering and regenerative medicine.

Smart bioinks are a key component of 4D bioprinting, enabling the printed 3D constructs to dynamically adapt, functionally remodel, or self‐assemble over time in response to stimuli, thereby mimicking the natural behaviors of living tissues [19, 37, 38]. Unlike conventional bioinks, which produce static structures, smart bioinks incorporate stimuli‐responsive materials, such as shape memory polymers (SMPs) [39, 40, 41] and shape‐morphing hydrogels (SMHs) [36, 42, 43], that allow printed constructs to evolve over time. This dynamic behavior is critical for replicating the native tissue microenvironments, where cells interact with and respond to ever‐changing biophysical and biochemical cues. For example, shape‐morphing bioinks can self‐fold into tubular, vascular‐like grafts, or form contractile cardiac patches, mimicking natural tissue development after printing [22, 44, 45, 46]. Additionally, smart bioinks enhance biological functionality by enabling on‐demand drug release, matrix stiffening to guide stem cell differentiation, or gradual degradation synchronized with tissue regeneration [47, 48]. Such capabilities address key challenges in tissue engineering and regenerative medicine, particularly physical and structural integration into host tissues and long‐term functionality. Furthermore, 4D‐bioprinted constructs fabricated with smart bioinks can reduce our reliance on invasive surgical treatments, as these structures can adapt to the defect geometry post‐implantation [49]. By bridging the gap between engineered scaffolds and living systems, smart bioinks pave the way for patient‐specific, adaptive therapies.

The development of 4D bioprinting has gained significant momentum in recent years (Figure 1e). While many recent reviews have comprehensively discussed the advancements and prospects of 3D printing/bioprinting and 4D printing technologies [19, 50, 51], there is a paucity of reviews specifically focusing on an updated and comprehensive overview of 4D bioprinting. For example, Ding and colleagues recently comprehensively reviewed the technologies, materials, stimuli, design, and applications of 4D printing [19]. However, the significance of smart bioinks for 4D bioprinting remains underemphasized in existing reviews. Therefore, in the current review, we aim to provide a historical overview and establish a conceptual framework of smart bioinks for 4D bioprinting. Furthermore, we comprehensively discuss the design and significant properties of smart bioinks in 4D bioprinting for fabricating dynamic tissues and organs. We first briefly introduce the mainstream bioprinting technologies used in 4D bioprinting and highlight their respective advantages and limitations. Subsequently, we thoroughly discuss the properties and design principles of smart bioinks for 4D bioprinting, emphasizing the importance of recent advances in AI‐driven smart bioink design. Additionally, we outline the commonly used biomaterials in smart bioinks and explore the wide range of applications of 4D bioprinting. Finally, we provide an in‐depth discussion on the current challenges and future perspectives regarding the application of smart bioinks in 4D bioprinting.

## 2 4D Bioprinting Techniques

3D bioprinting is the foundational technology of 4D bioprinting, enabling the construction of cell‐laden 3D structures that subsequently undergo changes in shape, configuration, and/or function in response to external or internal stimuli. According to ISO/ASTM 52900:2021 standard, there are seven distinct 3D printing techniques: material extrusion (ME), vat polymerization (VP), powder bed fusion (PBF), material jetting (MJ), binder jetting, sheet lamination, and directed energy deposition. Although these 3D printing techniques have been widely used to manufacture various 3D objects for diverse applications, most of them are unsuitable for printing smart bioinks used in 4D bioprinting. For instance, PBF, binder jetting, and sheet lamination processes have low biocompatibility and, when used to process cell‐laden materials, can significantly compromise cell viability. Therefore, 4D bioprinting techniques compatible with cell‐laden smart bioinks can be categorized into only three main types: inkjet‐based bioprinting, laser‐assisted and light‐based bioprinting, and extrusion‐based bioprinting. Table 1 provides an overview of the key characteristics of these three bioprinting technologies.

**TABLE 1:** Comparative analysis of various 3D bioprinting technologies. The images are adapted from [74] with permission.

3D bioprinting technologies	Advantages	Limitations	Resolution	Cell density	Typical cell viability	Representative biomaterials	Refs.
Inkjet‐based bioprinting 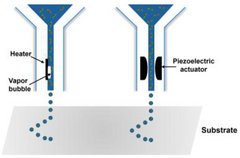	Low cost; High printing speed; High resolution; Relative high cell viability; Multi‐material printing;	Low viscosity of bioinks; Nozzle clogging; Low cell density; Weak mechanical properties; Limited bioink options	High (20–100 µm)	Low (1–10 × 10^6^ cells/mL)	>85%	Alg, HA, agarose, Pluronic F‐127	[75, 76]
Laser‐assisted and light‐based bioprinting 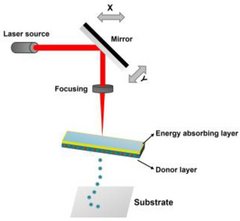	Nozzle‐free; Ultra‐high resolution; High cell viability; Wide cell density range; Multi‐material compatibility;	High cost; Complexity of controlling laser pulses; Risk of laser irradiation; Limited material choice;	High (10–50 µm)	High (1–50 × 10^6^ cells/mL)	>90%	Alg, gelatin methacryloyl (GelMA), collagen, Matrigel	[60, 61, 63]
Extrusion‐based bioprinting 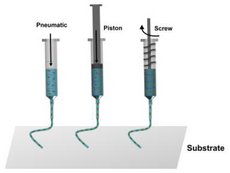	Compatibility with a wide material range; High viscosity; Scalability; High cell density; Structural integrity; Multi‐material & multi‐cell printing;	Low resolution; Nozzle clogging; Shear‐induced cell damage; Slower speed;	Medium (100–500 µm)	High (1–100 × 10^6^ cells/mL)	60%–90%	Alg, GelMA, collagen, Pluronic F‐127	[65, 77, 78]

### 2.1 Inkjet‐Based 3D Bioprinting

Inkjet‐based bioprinters eject cell‐laden bioinks through a nozzle, generating droplets that are subsequently deposited onto a substrate to construct 3D structures in a layer‐by‐layer manner [52, 53, 54]. Inkjet‐based bioprinting can be classified into two different categories: continuous inkjet bioprinting and drop‐on‐demand inkjet bioprinting. A continuous inkjet bioprinter is capable of ejecting continuous bioink droplets under pressure. Although the movement of these droplets can be controlled by electrical signals, the process lacks stability, and precise manipulation remains challenging. A drop‐on‐demand inkjet bioprinter enables precise control and positioning of droplets, as it utilizes an actuator to generate pressure pulses that eject droplets only when required. Based on the types of actuators, a drop‐on‐demand inkjet bioprinter can be a thermal or piezoelectrical system. In thermal drop‐on‐demand inkjet bioprinting, a voltage pulse is applied to the thermal actuator to heat the bioink locally. This leads to the formation, expansion, and eventual collapse of a vapor bubble, resulting in droplet ejection from the nozzle. In piezoelectric drop‐on‐demand inkjet bioprinting, a piezoelectric actuator expands or contracts in response to an applied voltage pulse. This deformation of the printing chamber generates pressure that forces droplets out of the nozzle.

Inkjet‐based bioprinting offers several advantages: (1) Affordability: owing to the use of commercial, off‐the‐shelf subsystems, the equipment cost of inkjet‐based bioprinters is relatively low; (2) High printing speed: inkjet‐based bioprinters can eject up to approximately 10 000 droplets per second; (3) High resolution: the resolution of this technique can be from 20 to 100 µm; and (4) Relative high cell viability: cell viability in inkjet‐based bioprinting generally reaches 85%–95%. However, inkjet‐based bioprinting also has several limitations: (1) Limited range of bioink viscosity: only bioinks with viscosities ranging from 3 to 12 mPa·s are suitable for bioprinting; (2) Potential nozzle clogging: the printer nozzles are prone to blockage; (3) Requirement for crosslinking treatments: additional crosslinking steps are often necessary to ensure structural integrity and fidelity of the printed constructs; and (4) Low cell density: due to the requirement for low‐viscosity bioinks, the cell concentration is typically limited to less than 10 × 10^6^ cells/mL.

### 2.2 Laser‐Assisted and Light‐Based 3D Bioprinting

The laser‐assisted 3D bioprinting technique employs the energy of a laser or light to pattern bioinks in a 3D layer‐by‐layer arrangement [55, 56, 57]. Laser‐assisted 3D bioprinting can be categorized into three main subtypes: laser‐induced forward transfer (LIFT), laser‐guided direct writing (LGDW), and selective laser cell bioprinting (SLCB). Currently, LIFT and LGDW are predominantly applied in single‐cell deposition [58, 59]. Light‐assisted 3D bioprinting refers to light‐based vat photopolymerization techniques, mainly including SLA, digital light processing (DLP), continuous liquid interface production (CLIP), two‐photon polymerization (2PP), and volumetric bioprinting. Among them, SLA and DLP have emerged as the dominant light‐based 3D bioprinting techniques due to their high precision and compatibility with cells and photocurable bioinks [60, 61, 62], whereas CLIP can achieve printing speeds up to hundreds of millimeters per hour—significantly faster than traditional light‐based methods—while maintaining high resolution (down to ∼50 µm).

Laser‐assisted and light‐based 3D bioprinting has many advantages, including a nozzle‐free design (eliminating clogging issues), high resolution (∼50 µm), and high cell viability (>95%). However, its drawbacks include the high cost of laser systems, the complexity of controlling laser pulses, the requirement for low‐viscosity bioinks (1–300 mPa·s), and the potential risk of laser‐induced cell damage. Overall, 3D bioprinting techniques that leverage laser‐ or light‐based mechanisms enable high‐precision cell patterning with minimal mechanical stress [60, 63].

### 2.3 Extrusion‐Based 3D Bioprinting

Extrusion‐based 3D bioprinting, derived from inkjet bioprinting systems, is one of the most widely adopted technologies for fabricating 3D constructs in tissue engineering [64, 65, 66]. In contrast to inkjet‐based bioprinting, which can only process bioinks with low viscosity (∼10 mPa·s), extrusion‐based bioprinting can handle materials with significantly higher viscosities, ranging from 30 to approximately 6 × 10^7^ mPa·s, through pneumatic or mechanical (piston‐ or screw‐driven) extrusion methods. Additionally, instead of ejecting discrete droplets as in inkjet bioprinting, extrusion‐based printing enables the deposition of continuous cylindrical filaments, allowing for layer‐by‐layer construction of complex 3D structures under controlled pressure. In extrusion‐based bioprinting, bioinks are loaded into a syringe, and by optimizing key printing parameters, such as pressure, printing speed, and temperature, continuous filaments are precisely extruded through the nozzle onto the substrate to form the desired 3D architecture. The resulting constructs are typically stabilized via crosslinking techniques such as exposure to UV light, thermal treatment, ionic solutions, or enzymatic reactions [67, 68].

Extrusion‐based 3D bioprinting technology possesses several advantages, including: (1) Compatibility with a wide range of biomaterials: many types of shear‐thinning solutions, including selected polymers and suspensions of ceramic or metal micro/nanoparticles [69, 70], that are amenable to cell encapsulation can be utilized for extrusion‐based 3D bioprinting. To facilitate cell integration, ceramic and metal phases can be incorporated into bioinks as particulate fillers to enhance structural support and printability while maintaining/enhancing cell viability. (2) High viscosity. Compared to inkjet‐based and laser‐assisted bioprinting, extrusion‐based bioprinting can process bioinks with much higher viscosity by adjusting printing parameters such as pressure and printing speed. (3) Scalability. Extrusion‐based bioprinting is capable of fabricating large‐scale 3D structures, such as human‐scale tissues or organs. For example, extrusion‐based 3D bioprinting enable to construct human‐scale tissues, such as cartilage patches, segments of blood vessels, and skin grafts, by meticulously depositing layer upon layer of living bioinks [71]. However, compared to inkjet‐based and laser‐based bioprinting technologies, extrusion‐based bioprinting generally offers lower printing resolution (∼100 µm). Furthermore, nozzle clogging is a common issue that is often difficult to avoid. In addition, the shear stress generated during the printing process may induce cell apoptosis and even death, compromising the viability of bioprinted constructs [72, 73].

## 3 Properties of Smart Bioinks and Bioprinted Structures

Smart bioinks are mostly cell‐laden polymeric biomaterials specifically designed for 4D bioprinting, allowing the printed structures to dynamically change over time in response to external or internal stimuli, such as temperature, pH, light, and moisture. These bioinks typically incorporate responsive polymers, nanoparticles, or biomolecules that impart properties such as shape‐memory behavior, self‐healing properties, or stimulus responsiveness to the printed structure [13, 79, 80]. In this regard, the most critical feature of smart bioinks in 4D bioprinting is their ability to respond to stimuli, enabling shape transformation, self‐assembly, or controlled degradation over time [81, 82]. Furthermore, like conventional bioinks used in 3D bioprinting, smart bioinks must exhibit sufficient printability, biocompatibility, and biodegradability, as well as ECM‐mimicking properties to support cell survival and functionality (Table 2).

**TABLE 2:** Key properties of smart bioinks for 4D bioprinting.

Properties	Specifications
Printability	Allowing continuous and consistent deposition Maintaining structural integrity in accordance with predefined design Possessing appropriate rheological properties (e.g., shear‐thinning behavior, viscoelasticity, and yield stress)
Biocompatibility	Eliciting no strong immune reaction/severe inflammatory response Supporting cell activities
Biodegradability	Having a controllable decomposition or degradation rate in a biological environment Degradation products eliciting no adverse cell responses
Stimuli‐responsive behavior	Enabling printed constructs to dynamically change shape or properties over time in response to stimuli Being adaptable to complex tissue anatomical structure
Cells	Suitable cell type and density High cell viability Being adaptive to changes in scaffold shape or properties
Bioactivity	Supporting cell proliferation and differentiation Facilitating desired physiological functions in vivo
Fidelity of printed structures	Maintaining their intended structure after printing Allowing for enhancement of structure integrity by modulating factors such as printing parameters, rheological properties, and crosslinking strategies

### 3.1 Printability

Printability is a fundamental requirement and a crucial consideration in evaluating the suitability of bioinks for 3D/4D bioprinting [83]. Generally, printability refers to the ability of bioinks to be continuously and consistently deposited while maintaining structural integrity according to a predefined design. This property significantly affects the final architecture of the printed construct, such as pore size, pore shape, and porosity, and, as a result, affects the mechanical properties and biological performance of printed structures. Many factors can influence the printability of bioinks, including structural design (e.g., pore shape, curvature, size, and layer thickness), material properties (e.g., cell density, viscosity, shear‐thinning behavior, and mechanical properties), and printing parameters (e.g., printing pressure, nozzle diameter, and printing speed) [84, 85]. Among these, layer thickness is a critical structural design parameter that strongly affects printability. Bioinks with poor printability may lead to inconsistent layer deposition, resulting in increased filament diameter, reduced pore size, and compromised printing resolution. Viscosity is another essential material‐related parameter [86, 87]. For example, gelatin and its derivatives, widely used biomaterials for 3D/4D bioprinting [88, 89], exhibit poor printability in extrusion‐based 3D bioprinting at low concentrations due to their low viscosities. The printability of gelatin‐based bioinks can be significantly improved when they are combined with other biomaterials to achieve enhanced viscosity [90, 91]. It should be noted that the printability of bioinks depends on the printing techniques used. For example, while extrusion‐based 3D bioprinting generally requires viscous bioinks, a high viscosity impairs the printability of bioinks in inkjet‐based 3D bioprinting as it can hinder droplet ejection, cause nozzle clogging, and reduce printing resolution. Thus, low‐viscosity bioinks are generally favored in inkjet‐based 3D bioprinting to achieve smooth and consistent droplet formation. Furthermore, the printing pressure used in extrusion‐based 3D bioprinting, which determines the extrusion rate of bioinks, should be appropriately adjusted to match the extrusion rate to the printing speed. Excessive pressure may lead to over‐deposition, resulting in increased filament thickness, reduced pore size, and pore shape distortion. Conversely, insufficient pressure can cause inconsistent extrusion of bioinks. For laser(light)‐assisted 3D bioprinting, the photo‐crosslinkable properties of the bioinks are the foremost characteristic influencing the printability. Effective photo‐crosslinking is essential for enabling rapid solidification and achieving high printing resolution [92].

**FIGURE 3:**
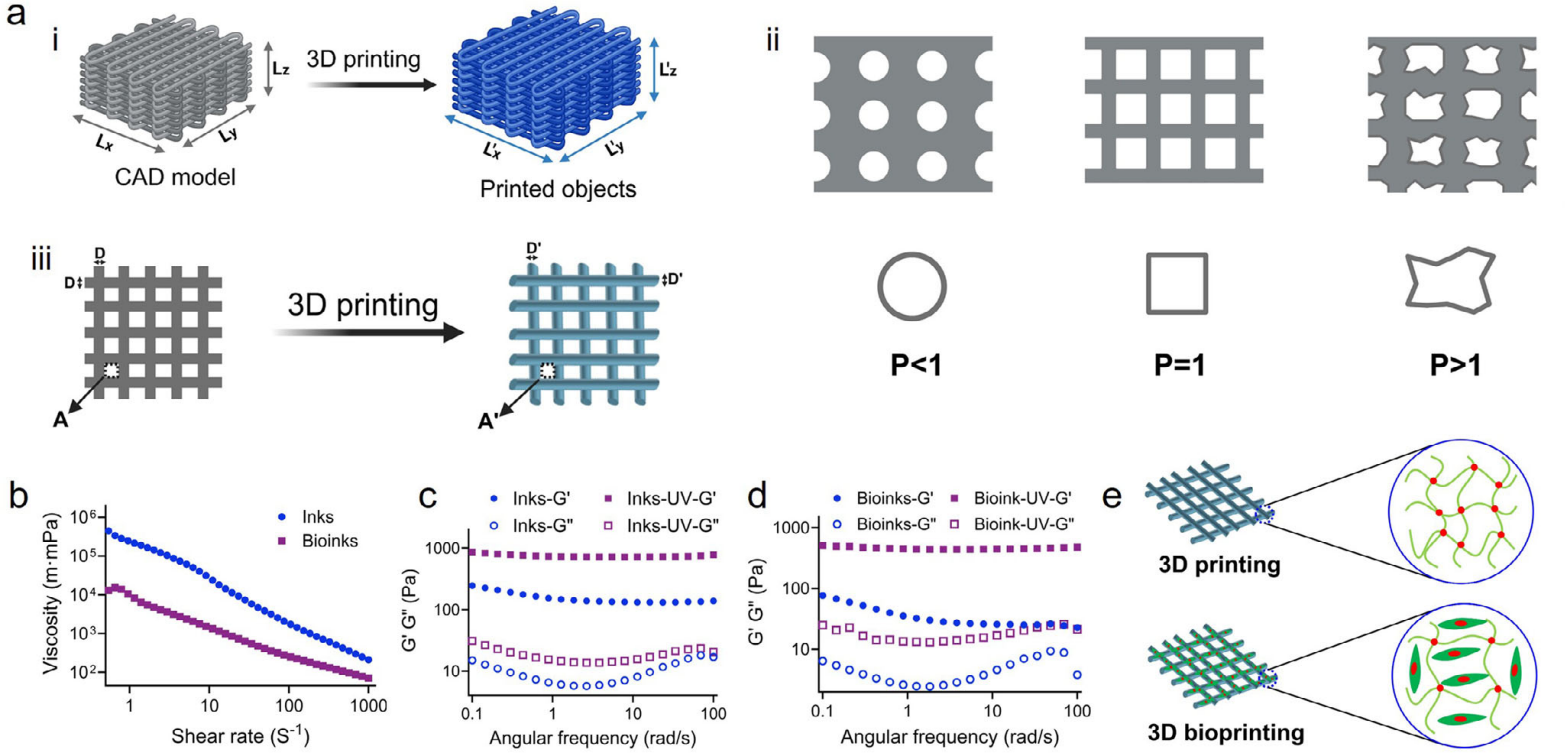
Evaluation of printability and effect of cells on rheological properties of bioinks and printed structures. (a) Printability evaluation. (i) Parameters used to evaluate the irregularity of a 3D printed object. (ii) Schematics of different pore printability. (iii) Parameters used to evaluate strand printability and diffusion rate. (b) Shear‐thinning behavior of inks and BMSC‐laden bioinks. Reproduced with permission [40]. Copyright 2024, Wiley. G’ and G” of (c) inks and (d) bioinks before and after UV crosslinking. Reproduced with permission [40]. Copyright 2024, Wiley. (e) Schematic illustration of cells in bioinks that interfere with polymer chain interactions. Figure 3a,e are created with BioRender.com.

Given the significance of printability in 3D printing, it is crucial to accurately evaluate and measure printability. Many methods and formulations have been developed and applied for printability evaluation. In general, indices that consider both the overall geometry and internal structure of printed objects, such as shape accuracy index, pore printability, strand printability, and diffusion rate, serve as key parameters for assessing printability (Figure 3a). The shape accuracy index of a 3D‐printed object is measured by comparing the experimentally obtained dimensions in three axes (X, Y, and Z) with the designed dimensions (Equation 1), reflecting the overall accuracy of 3D/4D bioprinting:

(1) $$I\;\left(\%\right)=100\frac{L^{\prime }x,y,z}{Lx,y,z}$$

where *I* denotes the shape accuracy index of a 3D‐printed object, *L'x,y,z* represents the experimentally measured dimensions (X, Y, Z) of the object, and *Lx,y,z* refers to the corresponding designed dimensions (X, Y, Z).

Pore printability is a commonly used metric to quantify the deviation between the actual and designed pore shapes (Equation 2):

(2) $$P=\frac{L^{2}}{16A}$$

where *P* is the pore printability, *L* is the perimeter of the pore, and *A* is the area of the pore.

Strand printability refers to the degree of conformity of the experimentally measured strand diameter to the designed strand diameter (Equation 3):

(3) $$P=\frac{D^{\prime }}{D}$$

where *P* is the strand printability, *D*′ is the experimentally measured strand diameter, and *D* is the designed experimental diameter.

Diffusion rate serves as an indicator of the extent to which the actual pore area deviates from the designed pore area (Equation 4):

(4) $$D\;\left(\%\right)=100\frac{A-A^{\prime }}{A}$$

where *D* is the diffusion rate, *A* is the area of the design pore, and *A*′ is the area of the actual pore.

### 3.2 Biocompatibility

Biocompatibility is the primary criterion for bioinks used in tissue engineering and regenerative medicine applications. A biocompatible bioink supports key cellular activities (e.g., cell adhesion, migration, proliferation, and differentiation) without eliciting strong immune reactions or severe inflammatory responses. Such materials not only ensure high cell viability and growth, but also facilitate the transmission of molecular and mechanical signals and establish favorable interactions with both endogenous tissues and the immune system. These factors are critical to achieving successful integration and functional performance after transplantation [93]. However, it should be noted that biocompatible materials may still trigger inflammatory responses after being implanted in vivo. Previous studies have revealed that an inflammatory response is inevitable upon the implantation of biomaterials [94]. Moreover, a controlled inflammatory response can be beneficial for tissue regeneration [95, 96].

Implanted biomaterials, whether metallic, ceramic, polymeric, or their composites, come into contact with biological tissues and interact with the surrounding environment, including water (at different pH levels), enzymes, and cells. These interactions lead to potential corrosion or degradation and may result in the release of degradation products, such as ions and debris. These by‐products must also be biocompatible and should not provoke significant host immune responses. Furthermore, they should be readily cleared by the host's physiological mechanisms. For instance, carbon nanotubes (CNTs) have been shown to promote the growth and functions of cardiac and neural cells in vitro due to their high electrical conductivity [97, 98]. However, the long‐term biocompatibility of CNTs remains a concern, since their elimination from the host organism and potential bioaccumulation depend on factors such as nanotube dimensions, surface functionalization, and administration route. While certain studies have reported the persistence of CNTs in vivo, others have shown clearance via renal or biliary pathways based on surface modification [99]. It is important to note that in some cases, although the materials incorporated in bioinks are biocompatible themselves, toxic solvents or chemicals are used as crosslinking agents during fabrication to achieve structural integrity. For example, gelatin‐based hydrogels have traditionally been crosslinked with glutaraldehyde to enhance their mechanical properties [100, 101]. Similarly, GelMA‐based bioinks are typically combined with photoinitiators, such as 2‐hydroxy‐4’‐(2‐hydroxyethoxy)‐2‐methylpropiophenone, to facilitate chemical crosslinking upon exposure to UV light. To mitigate possible downstream toxicity in host tissues that has yet to be fully understood, residual crosslinking agents should be thoroughly removed prior to implantation or remain within acceptable safety limits.

In summary, bioinks, as well as the resulting printed constructs, must meet biocompatibility requirements. Various international standards require significantly different biological evaluations of these materials [102]. Typically, such assessments encompass in vitro and in vivo studies assessing the cytocompatibility, genotoxicity, sensitization, irritation, acute and chronic toxicity, hemocompatibility, reproductive and developmental toxicity, and carcinogenic potential.

Upon in vivo implantation, the printed constructs are exposed to a complex biological environment characterized by continuous fluid flow, enzymatic activity, pH fluctuations, and immune cell infiltration. These conditions, which differ greatly from controlled in vitro settings, can accelerate or modify the degradation pathways of stimuli‐responsive materials. For example, the degradation of shape memory polymers like PLATMC and PLA can generate acidic by‐products (e.g., lactic acid), which may accumulate locally and potentially lead to chronic inflammation, tissue necrosis, and delayed healing [103]. Moreover, the addition of nanoparticles (e.g., MXene, Fe_3_O_4_, black phosphorus) to endow stimuli‐responsiveness brings about additional safety issues. The long‐term biodistribution, accumulation, and clearance of these nanomaterials have not been fully elucidated. For instance, although MXene nanosheets possess excellent photothermal properties, the potential bioaccumulation of these nanosheets in the reticuloendothelial system and their long‐term cytotoxicity require systematic research [104]. Similarly, although the degradation products of black phosphorus nanosheets, i.e., phosphates and phosphites, are generally biocompatible, their potential to disrupt local phosphate homeostasis or induce oxidative stress needs to be carefully evaluated [105].

The immune response to implanted biomaterials plays a crucial role in their long‐term success. Smart bioinks employed in 4D bioprinting present distinctive immunological challenges. First, the repeated or continuous application of external stimuli (e.g., NIR irradiation, magnetic and electrical fields) to initiate shape morphing can induce local thermal or mechanical stress, which could activate inflammatory pathways. Second, mechanical forces induced by dynamic 4D constructs undergoing swelling, contraction, or folding are likely to be detected by resident immune cells (as well as mechanosensitive cells) [106, 107]. These mechano‐immunological interactions can regulate macrophage polarization, altering the equilibrium between pro‐inflammatory and pro‐regenerative phenotypes. While controlled inflammation is needed for tissue regeneration, excessive or persistent inflammatory responses can result in fibrous encapsulation and implant failure [108]. Hence, the immunomodulatory properties of smart bioinks, including their degradation products and the mechanical signals they generate, need to be thoroughly characterized. Moreover, many synthetic stimuli‐responsive polymers (e.g., PNIPAM) and crosslinking agents (e.g., photoinitiators such as Irgacure 2959) have been shown to trigger immune responses in preclinical models [109]. The utilization of UV or near‐UV light during fabrication may cause protein denaturation or DNA damage in encapsulated cells if not carefully controlled. Strategies such as surface modification with immunomodulatory molecules or the incorporation of anti‐inflammatory agents may therefore be essential to alleviate these adverse effects.

### 3.3 Biodegradability

Once a biomaterial is implanted in vivo, biodegradation occurs through its interactions with various biological components. Biodegradability refers to the ability of a material to decompose or degrade in a biological environment and is another essential property for bioinks or 3D printed constructs for tissue engineering and regenerative medicine applications [110]. According to the definition provided by the International Union of Pure and Applied Chemistry (IUPAC), biodegradation is degradation caused by enzymatic processes resulting from cellular activities [111]. Similarly, in the USA, Mikos and Temenoff described biodegradation as the chemical breakdown of a material mediated by any component of the physiological environment—such as water, ions, proteins, cells, or bacteria—resulting in smaller constituents or low molecular weight products that can be processed, resorbed, or cleared by the body [112].

With polymeric hydrogels constituting the primary components, current bioink materials or 3D printed objects used in 4D bioprinting are typically biodegradable. Ideally, their biodegradation rate should align with the pace of tissue regeneration. It should be noted that non‐biodegradable, stable biomaterials, which can maintain their structural integrity and mechanical properties over long periods in biological environments, have also been widely employed to fabricate 3D constructs with sustained functionality [113]. For example, several metallic biomaterials have been employed to improve the mechanical strength of bone and dental implants [74, 114]. Additionally, bioceramics such as alumina, zirconia, and hydroxyapatite (HAp), by virtue of their high biocompatibility (and excellent osteointegration for HAp), have been used in bioinks in the form of nanoparticles for bone tissue engineering [115, 116].

Although most bioink materials are biodegradable, their degradation rates vary significantly depending on the material types. Natural polymers (e.g., collagen, gelatin, Alg, etc.) degrade rapidly in biological environments. Ceramics such as HAp and synthetic polymers [e.g., polylactic acid (PLA), polycaprolactone (PCL), poly(lactic‐co‐glycolic acid) (PLGA)] exhibit relatively slower degradation rates. Therefore, natural polymers can be combined with synthetic polymers or ceramics to fabricate composite bioink materials with optimized degradation rates. Additionally, natural polymers can be chemically crosslinked to preserve their structural integrity and functional properties over extended periods following implantation. For example, methacrylated polymers are a widely used material type in 4D bioprinting due to their ability to undergo photo‐crosslinking and form stable 3D constructs [117, 118, 119]. Moreover, architectural design (e.g., pore size and porosity) significantly influences the degradation behavior of 3D printed constructs. Compared to solid structures, porous scaffolds tend to degrade more rapidly, in part as a result of increased surface area [120].

### 3.4 Stimuli‐Responsive Behavior

Stimuli‐responsiveness is a defining characteristic of bioinks for 4D bioprinting, as it bridges the gap between static 3D scaffolds and dynamic, functional tissues. Leveraging smart bioinks capable of dynamically altering their shapes and functionalities in response to external or internal stimuli, 4D bioprinted structures mimic the adaptive remodeling processes observed in native tissues. This capability is a transformative advancement in regenerative medicine, as it enables engineered constructs to dynamically integrate with evolving microenvironments in vivo.

Temperature‐ or moisture‐responsive bioinks enable the fabrication of shape‐morphing hydrogels that can conform to the complex anatomical features of tissue defects after implantation [121, 122]. For instance, defects in human cranial bone and uterine tissue are often irregular in shape and frequently exhibit curvatures. Following 4D bioprinting, bioinks can undergo shape transformation to geometrically match the irregular defects, thereby achieving optimal therapeutic outcomes. Moreover, mechano‐sensitive bioinks have the potential to replicate the load‐responsive behavior observed in cartilage and bone [123]. In addition to structural adaptation, stimuli‐responsive bioinks can also be utilized for on‐demand drug delivery triggered by, for example, localized biochemical changes (e.g., release of inflammatory cytokines) to achieve targeted therapeutic responses [48, 124]. Furthermore, in biohybrid systems and soft robotics, bioinks that respond to external stimuli such as light or magnetic fields enable remote‐controlled actuation, opening new possibilities for minimally invasive medical interventions [125, 126]. The capacity to respond to multiple stimuli—such as concurrent thermal, chemical, and electrical signals—enhances biomimicry, enabling engineered constructs to replicate complex physiological behaviors like neuronal signaling and cardiac pulsation [127, 128].

Achieving reliable responsiveness to stimuli without compromising printability, shape fidelity, or biocompatibility remains a significant challenge. Advances in dynamic covalent chemistry, nanocomposite formulations, and bioengineered motifs (e.g., peptide‐based molecular switches) are helping to overcome these limitations and facilitate the development of next‐generation bioinks [49, 129]. By imparting life‐like adaptability to bioprinted constructs, stimuli‐responsive bioinks hold the potential to not only transform personalized medicine but also advance our understanding of tissue morphogenesis and disease mechanisms, ultimately narrowing the gap between synthetic and natural living systems.

### 3.5 Cells

Cells function as the fundamental living units that participate in or drive the transformation of smart bioinks from passive materials into dynamic, biologically functional systems for 4D bioprinting. Compared to differentiated cells, stem cells—such as adult tissue‐derived mesenchymal stem cells (MSCs) and induced pluripotent stem cells (iPSCs)—are more commonly utilized in bioinks due to their self‐renewal capacity, multilineage differentiation potential, and secretory functions [34]. With the ability to differentiate into multiple cell lineages, stem cells can be used to bioprint various tissue types, including bone, cartilage, and vascular structures [12]. Moreover, the paracrine signaling of stem cells contributes to angiogenesis and immune modulation [130]. In addition, the inclusion of cells can enhance the responsiveness of bioinks to external stimuli [36, 121]. For example, cytoskeletal reorganization activated by mechanosensing can guide or augment shape morphing of smart scaffolds [131], while cellular metabolic activities can trigger pH‐ or enzyme‐mediated matrix remodeling [132]. Ding and coworkers demonstrated that tissue constructs fabricated using a smart bioink with mechanically adaptive properties can undergo cell contractile force (CCF)‐driven shape morphing [131]. When cells are spatially patterned with precise arrangement and localization within the bioink, CCFs mediate multidirectional and progressively enhanced structural transformations, facilitating the formation of complex tissue architectures.

Cell density is a critical factor that influences the characteristics of bioinks and the printed 3D constructs, particularly the structural integrity and dynamic behavior [133, 134]. While cell densities typically range from 1 to 10 × 10^6^ cells/mL in many extrusion‐based bioprinting studies, the optimal cell density is highly dependent on cell type, bioink composition, printing technology, and target tissue application. Moreover, recent studies have demonstrated successful bioprinting at substantially higher densities, reaching 1 × 10^8^ cells/mL or greater [134]. Higher cell densities could enhance biological activities and lead to faster tissue maturation and responsive remodeling, but they may compromise printability in extrusion‐based printing due to changed viscosity. Furthermore, it can lead to cell leakage from the printed construct, particularly during the initial post‐printing phase [135]. In contrast, lower cell densities (<1 × 10^6^ cells/mL) generally improve the flow properties of bioinks but may result in insufficient cellular interactions needed for effective tissue formation.

While incorporating cells in bioinks—and consequently into 3D bioprinted structures—can enhance the implant's regenerative potential, it also introduces several limitations. Notably, the inclusion of living cells has excluded the use of certain 3D printing technologies that are highly effective for fabricating acellular scaffolds from being used in bioprinting applications. Furthermore, the inclusion of cells has restricted the range of bioink materials to those compatible with 3D bioprinting conditions. For instance, extrusion‐based 3D printing is one of the most widely used techniques for producing 3D scaffolds due to its compatibility with a broad spectrum of biomaterials [136]. Almost all types of shear‐thinning materials, including polymers, ceramics, and metals, can be used as inks to fabricate 3D structures through extrusion‐based 3D printing. For example, fused deposition modeling (FDM), an extrusion‐based 3D printing technique, has been widely utilized for the 3D printing of synthetic biodegradable polymers such as PCL, PLA, and PLGA [137, 138]. However, in extrusion‐based 3D bioprinting, where cells are suspended in cell‐laden bioinks, the types of suitable biomaterials are significantly reduced. Synthetic biodegradable polymers such as PCL, PLA, and PLGA typically require heating to high temperatures or dissolution in organic solvents to produce ink solutions suitable for 3D printing. However, due to the application of high temperatures or toxic solvents, these materials are unsuitable for preparing cell‐laden bioinks used in 3D bioprinting. Currently, only a limited number of natural polymers—such as collagen, fibrin, gelatin, HA, chitosan, carrageenan, Alg, cellulose, and gellan gum—as well as a few synthetic polymers including poly(ethylene glycol diacrylate) (PEGDA) and poly(N‐isopropylacrylamide) (PNIPAM), are employed to prepare cell‐laden bioinks for 3D/4D bioprinting [13, 139, 140]. Most other biomaterials used only serve as low‐concentration additives within 3D/4D bioprinting bioinks to enhance cellular activities such as migration, attachment, proliferation, and differentiation [115]. To date, nearly all successful bioinks have been developed in the form of hydrogels [141].

Cells within a bioink occupy a specific volume, which is determined by cell size and density [83]. The cell‐occupied volume within the polymeric matrix affects the rheological properties of the bioink as well as the efficiency and degree of polymer chain crosslinking (crosslinking is required in most cases). This, in turn, impacts the printability of bioinks and the fidelity of printed structures. For example, when bone marrow‐derived MSCs (BMSCs) were encapsulated in GelMA‐based inks at a concentration of 1 × 10^6^ cells/mL, the viscosity of cell‐laden bioinks decreased, and the shear‐thinning effect was attenuated (Figure 3b) [40]. Moreover, the storage modulus (*G*′) and loss modulus (*G*″) of cell‐laden bioinks were lower than those of cell‐free GelMA inks in both crosslinked and non‐crosslinked states (Figure 3c,d). This phenomenon could be attributed to the weaker interactions among adjacent GelMA polymer chains due to the obstruction and interference by BMSCs, resulting in a reduction in bioink viscosity and crosslinking degree and efficiency (Figure 3e). Consequently, the printability of GelMA‐based bioinks was compromised. Therefore, compared to 3D printed structures, 3D bioprinted structures generally exhibit inferior structural fidelity.

It is critical to maintain high cell viability (typically >80%) during and after the bioprinting process [142, 143]. The viability of cells in bioinks and printed hydrogels is mainly determined by the cell type, printing technology used, bioink concentration, and crosslinking density [142, 144]. Generally, differentiated cells exhibit higher immediate post‐printing viability owing to their more robust cytoskeletal architecture and greater resistance to mechanical stress [145, 146]. In contrast, undifferentiated stem cells are typically more vulnerable to the shear and pressure during printing. However, stem cells may show higher cell viability after long‐term culture as they can differentiate and remodel the matrix during tissue maturation. As mentioned above, different printing technologies generate distinct microenvironments during the printing process, profoundly influencing cell viability. Compared with inkjet‐based and laser‐assisted 3D bioprinting techniques, extrusion‐based 3D bioprinting employs mechanical forces to extrude bioinks, which generates shear stress that may induce cell damage and apoptosis. Consequently, extrusion‐based 3D bioprinting generally results in the lowest cell viability among these techniques. Moreover, a high bioink concentration and/or crosslinking density can result in a dense polymer hydrogel network. This leads to high internal stress and limits nutrient diffusion, reducing cell viability. For example, Ouyang and coworkers investigated the viability of astrocytes encapsulated in GelMA hydrogels with varied GelMA concentrations (2.5 to 10 wt%) [147]. They found that 2.5 and 3 wt% GelMA yielded the highest cell viability, attributed to the soft mechanical properties and loose hydrogel network.

### 3.6 Bioactivity

In addition to the fundamental requisite of biocompatibility, bioactivity of smart bioinks is another crucial characteristic pertaining to their ability to support cell proliferation, differentiation, and biological functions within a bioprinted construct. The bioactivity of smart bioinks plays an important role in engineering functional tissues and organs that accurately recapitulate in vivo properties. The bioactivity of smart bioinks is conferred in part by the polymeric hydrogel network, but is frequently augmented by additional incorporated components, such as nanoparticles, growth factors, biomolecules, and drugs.

In extrusion‐based bioprinting, bioinks composed of shear‐thinning natural polymers (e.g., gelatin, Alg, HA, and collagen) not only exhibit improved printability without compromising internal bonding, but also substantially enhance cell activities (e.g., cell spreading, migration, proliferation, and differentiation) and promote cell survival by reducing detrimental shear force. Moreover, recent studies indicate that bioinks with loose and dynamic (reversible) crosslinks promote better cell survival and spreading compared to those with tight, static crosslinks [148, 149].

Smart bioinks for 4D bioprinting should support cell proliferation, which is needed to ensure the long‐term viability and functional performance of engineered tissues. The bioink composition plays a critical role in cell proliferation. In contrast to synthetic polymers, natural polymers such as gelatin and collagen mimic the native ECM and provide cell‐adhesive motifs, such as RGD (arginine‐glycine‐aspartic acid) peptides, thereby promoting cell proliferation in bioprinted hydrogels [150, 151]. Additionally, different growth factors, such as fibroblast growth factor 2 (FGF‐2) and epidermal growth factor (EGF), have been incorporated in bioinks to construct sustained release systems with improved bioactivity [152]. Among different growth factors, FGF‐2 is known to accelerate the proliferation of various cell types, including fibroblasts, MSCs, and endothelial cells, by stimulating mitogenic signaling pathways such as MAPK/ERK and PI3K/AKT [153]. Additionally, FGF‐2 can enhance angiogenesis in vascularized tissues, thereby improving oxygen and nutrient supply to proliferating cells [154]. Furthermore, the proliferation of encapsulated cells can also be modulated by adjusting the mechanical properties of bioinks and printed hydrogels. Compared to hard hydrogels, softer hydrogels (1–10 kPa) generally promote faster cell spreading and division, and stress‐relaxing materials enable more efficient dynamic remodeling [155].

Smart bioinks are increasingly designed to precisely control cell differentiation and guide the formation of complex, functional tissues. To this end, their properties can be enhanced and diversified through various modifications. With the incorporation of tissue‐specific biochemical cues, such as growth factors [bone morphogenetic proteins (BMPs), vascular endothelial growth factor (VEGF), and nerve growth factor (NGF)], bioinks enable precise regulation of stem cell differentiation [156]. Nanoparticles have also been incorporated into bioinks to stimulate tissue regeneration, regulate angiogenesis, and promote stem cell differentiation [115]. For example, ceramic nanoparticles, such as HAp, bioactive glass (BG), and tricalcium phosphate (TCP), have demonstrated significant efficacy in enhancing the osteogenic differentiation of human MSCs within bioprinted constructs. Similarly, the addition of conductive elements (e.g., graphene) or piezoelectric materials can promote electrophysiological maturation of neural and cardiac tissues [157]. The dynamic nature of 4D bioprinted hydrogels allows for temporal control over cell differentiation and immunomodulation, whereby stimuli such as temperature, pH, and light can be used to trigger the sequential occurrence of biophysical and biochemical signals, thereby mimicking natural developmental processes [158].

### 3.7 Fidelity of Printed Structures

Fidelity is used to evaluate the structural differences between the designed and printed objects. This index can also be defined as the ability of printed objects to maintain their intended structure. Therefore, fidelity determines the resolution of 3D/4D bioprinting [159]. A printed structure with poor fidelity may experience diffusion of printed filaments, which increases filament diameter and decreases thickness, impairing 3D/4D bioprinting resolution. Furthermore, printed objects with poor fidelity are unable to maintain their spatial structures and often collapse, particularly during the fabrication of multilayered architectures. Fidelity is closely related to the pore shape and size of printed objects. In tissue engineering applications, 3D scaffolds rely on pores for oxygen and nutrient transport and waste removal. For constructs with poor fidelity, altered pore morphology and size can compromise essential mass transport functions.

Several key factors can significantly influence the fidelity of extrusion‐bioprinted objects. First, printing parameters such as pressure, printing speed, nozzle geometry, and diameter determine the structural arrangement of deposited filaments, thereby affecting the fidelity of printed objects. Additionally, the rheological properties—particularly viscosity—of bioinks significantly influence the fidelity of constructs fabricated by extrusion‐based 3D bioprinting. Generally, polymer bioinks at high concentrations exhibit greater viscosity, resulting in printed objects with enhanced shape retention after 3D bioprinting. Furthermore, the crosslinking strategies used also affect the fidelity of printed objects. Following 3D bioprinting, physical or chemical crosslinking is commonly applied to stabilize the final structure and minimize deformation. For example, constructs bioprinted from Alg‐based bioinks can be immersed in Ca^2^^+^‐containing baths to induce ionic crosslinking of Alg polymers, thereby improving their shape fidelity [70, 160]. Moreover, the speed of crosslinking plays an essential role in affecting the fidelity of printed structures. Photo‐ and ionic crosslinking offer high speed (seconds or less), enabling rapid stabilization and high fidelity in extrusion bioprinting by preventing filament spreading. In contrast, slower thermal and chemical crosslinking often compromise fidelity due to gravity‐driven deformation before full gelation, though they may improve interlayer adhesion.

## 4 Design of Smart Bioinks

### 4.1 AI‐Driven Smart Bioink Design

The required properties of smart bioinks discussed above can guide the design of new bioinks for 4D bioprinting. The development of high‐performance smart bioinks with optimal bioprintability, responsiveness, and biocompatibility remains challenging due to the complexity of balancing these multifaceted properties. Currently, 4D bioprinting at its nascent stage still faces the significant challenge of a limited availability of smart bioinks. This underscores the need for innovative bioink design strategies.

**FIGURE 4:**
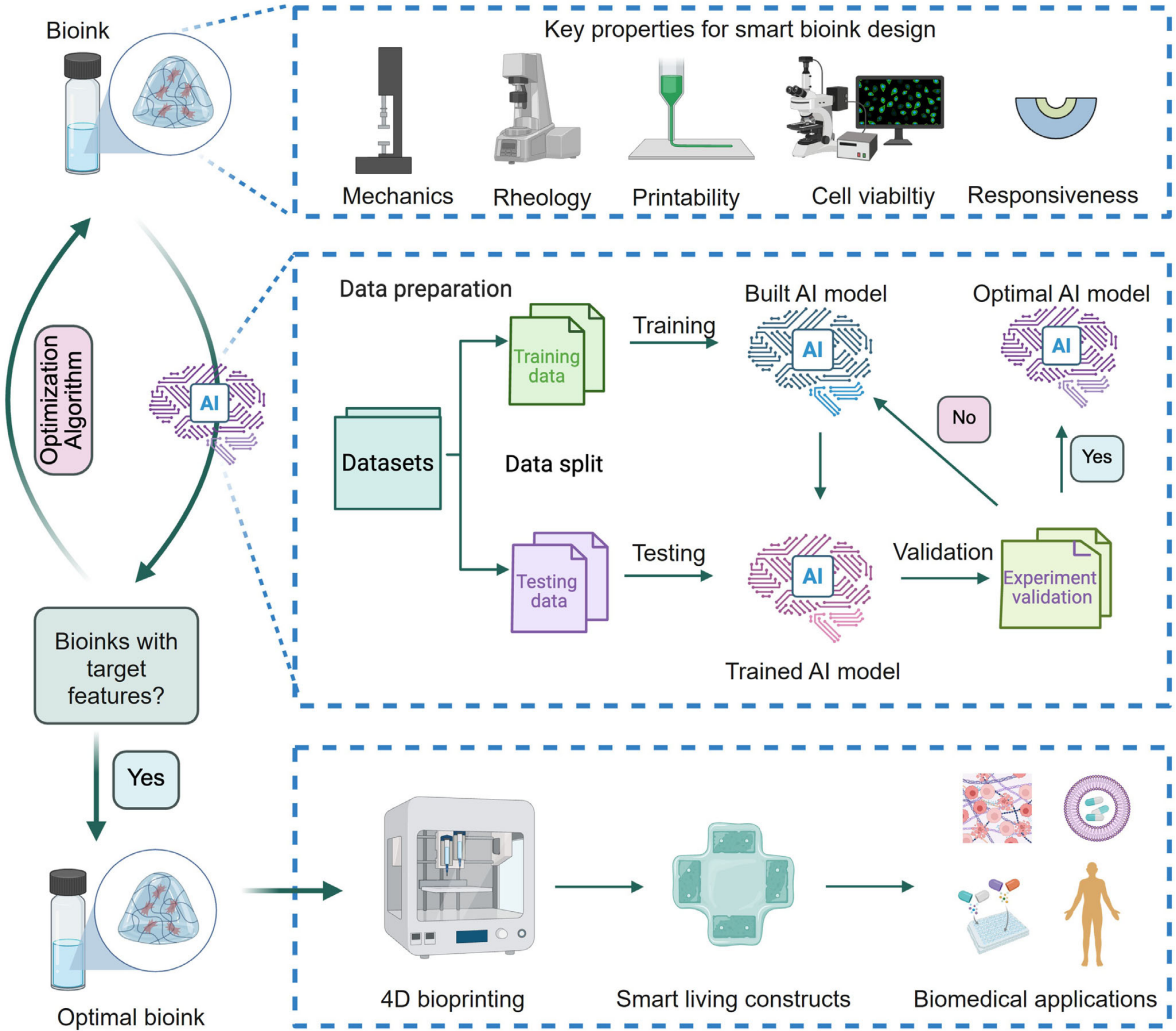
Schematic illustration of AI‐driven smart bioink design for 4D bioprinting. AI can facilitate bioink optimization by enabling high‐efficiency prediction and refinement of formulations and bioprinted construct designs. Created with BioRender.com.

Traditional bioink design relies heavily on the expertise of skilled researchers, who select biomaterials based on prior knowledge and desired properties, followed by extensive empirical testing. This trial‐and‐error strategy (i.e., Edisonian approach) necessitates numerous iterative experiments to refine formulations, making it time‐consuming, labor‐intensive, and costly. In recent years, artificial intelligence (AI), particularly machine learning (ML), has emerged as a transformative tool with the potential to revolutionize diverse fields, including regenerative medicine [161, 162]. AI‐driven approaches are increasingly instrumental in the design of smart bioinks, offering data‐driven solutions to optimize their properties for 4D bioprinting. Unlike conventional Edisonian approaches, ML provides a powerful, data‐driven framework to streamline bioink optimization by predicting and refining formulations with high efficiency (Figure 4). The ML‐driven design process begins with clearly defining the target properties, such as bioprintability, cell viability, and stimuli‐responsiveness. For single‐property optimization, a precise metric is established. For example, a printability score is generated based on filament formation and layer stacking in extrusion‐based bioprinting [163]. For multi‐property optimization, composite objective functions or multi‐objective optimization techniques are employed to navigate trade‐offs, such as balancing bioprintability with cell viability or mechanical stability [164].

**FIGURE 5:**
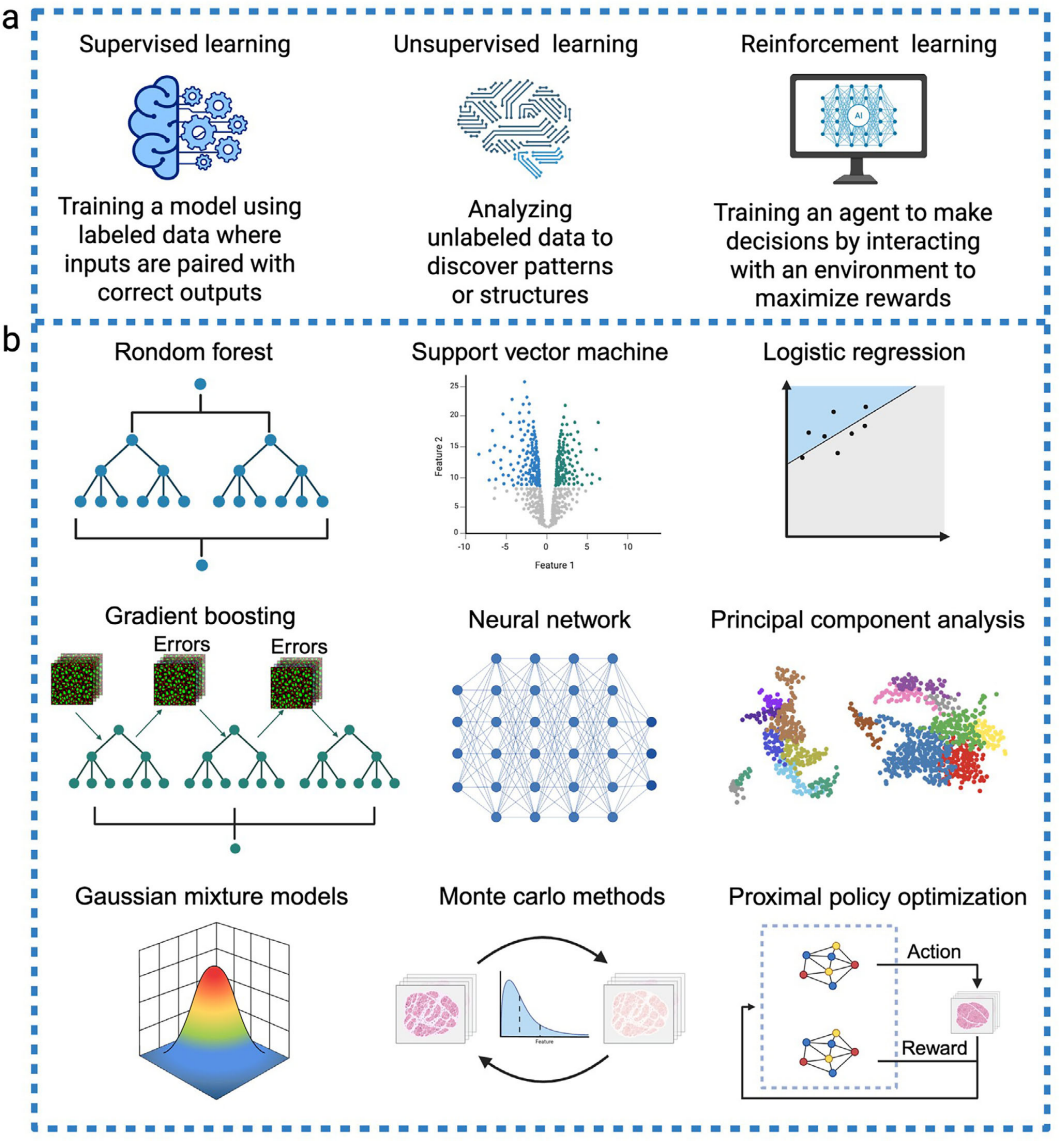
Conceptual framework of machine learning (ML). (a) Supervised, unsupervised, and reinforcement learning paradigms. (b) Representative ML models commonly applied in biomedical engineering. Created with BioRender.com.

The next step involves designing a series of bioink formulations by systematically tuning the relevant parameters, such as concentration, molecular weight, and ratio of different components. Experiments or computational simulations are conducted to quantify relevant properties, such as viscosity, filament size, shape fidelity, and cell viability, with data accurately recorded to construct robust datasets. An appropriate ML algorithm is then built based on the dataset and optimization goals. ML algorithms are broadly categorized into supervised learning, unsupervised learning, and reinforcement learning (Figure 5) [162]. Supervised learning methods, such as random forest, gradient boosting, logistic regression, support vector machines, and neural networks, utilize labelled datasets comprising input features (e.g., material compositions) and corresponding output properties (e.g., viscosity, cell viability) [165]. Through this supervised training process, the algorithm learns predictive mappings between inputs and outputs, reflecting statistical associations rather than causal relationships. Establishing causality would require dedicated causal‐inference frameworks, such as randomized interventions or structural causal models, which are rarely employed in this context. Conversely, unsupervised learning methods, such as principal component analysis and Gaussian mixture models, analyze unlabeled datasets to uncover intrinsic patterns or structures within the input data [166]. Reinforcement learning algorithms, such as Monte Carlo methods and proximal policy optimization, learn optimal actions through trial‐and‐error within an environment, aiming to maximize cumulative rewards [167]. Once trained, an ML model can iteratively suggest new formulations and predict their outcomes; these predictions are then experimentally validated and fed back into the model to refine its predictive accuracy over successive iterations. For instance, Gong's group reported a data‐driven methodology employing ML to engineer high‐performance acellular hydrogels, yielding significant enhancements in adhesive strength with maximum values surpassing 1 MPa, from an initial dataset comprising 180 bioinspired hydrogels‐[168]. In another study, Mei and colleagues applied ML to investigate the relationship between the adhesion of human pluripotent stem cells (hPSCs) and the properties of polyacrylate [169]. Partial least squares regression, an ML method, was utilized to correlate polymeric surface properties, including wettability, roughness, elastic modulus, and chemistry, with biological performance, identifying optimal hPSC adhesion substrates with high acrylate content and moderate wettability (water contact angle ∼70°).

The ML‐driven approach to smart bioink optimization has various advantages over conventional methods. It substantially reduces the number of experiments required, thereby saving time and resources. It also enables the exploration of a broader range of formulations, uncovering optimal bioinks that might be overlooked in traditional approaches. Furthermore, the scalability of ML enables efficient development of novel bioinks, thereby extending the library of smart bioinks for diverse 4D bioprinting applications. Despite this promise, it should be noted that most ML and AI studies discussed here span a range of related but distinct problem settings. Many published examples focus on general hydrogel or biomaterial ink design, material‐property prediction, printing‐parameter optimization, or acellular stimuli‐responsive 4D systems, rather than living 4D bioinks. Direct end‐to‐end ML‐guided design of cell‐laden smart bioinks capable of programmed 4D transformation therefore remains comparatively underexplored. Although these related studies presented in this section provide valuable methodological foundations, their findings should be extrapolated with caution, as cell‐laden systems involve additional complexities, including cytocompatibility constraints, dynamic cell–material coupling, and donor‐, cell source‐, and batch‐dependent biological variability.

Moreover, AI‐driven design is of particular significance for the development of endogenous stimuli‐responsive bioinks. Given the complexity of biological recognition, signal transduction, and in vivo variability, predictive modeling is essential to optimize responsiveness without compromising printability and biocompatibility.

### 4.2 Printability Optimization

Printability, the primary consideration in smart bioink design, is affected by various factors, such as composition and polymer concentration. ML has been increasingly applied to predict the printability of different bioink formulations for efficient optimization. Viscosity is an important property for evaluating a bioink's printability. ML approach has been utilized to streamline the process of identifying the optimal bioink compositions with suitable viscosity from a vast array of heterogeneous bioink options. An ML model for predicting the viscosity of polymer nanocomposites was proposed by combining ML algorithms with nonequilibrium molecular dynamics (NEMD) [170]. In this completely computational prediction model, the viscosity was determined using NEMD based on the conditions of shear rates, nanoparticle loading, and temperature. The NEMD‐generated data were then used to train four ML models, including linear regression (LR), support vector machine (SVM), decision tree (DT), and extreme gradient boosting (XGBoost). XGBoost showed the best performance in viscosity prediction, which was validated by comparison with physics‐based Cross, Carreau, and Herschel‐Bulkley models [170]. Similarly, Xu and colleagues established a constraint‐based Bayesian optimization (BO) ML model to predict the viscosity of hybrid bioinks composed of Alg, carboxymethyl cellulose, and TEMPO‐oxidized nanofibrillated cellulose [164]. This BO‐based ML model was employed to simplify the process of identifying printable hydrogels for extrusion‐based bioprinting by predicting and validating the viscosities of a series of bioink formulations. However, the above‐mentioned studies on bioink viscosity prediction did not consider the influence of shear rate, a critical parameter for nozzle‐based bioprinting that affects flow behaviour and viscosity. To address this issue, ML models such as polynomial fit, decision tree, and random forest were employed to predict the viscosity based on the component weights and shear rates [171]. The experimental data used for training and validation were obtained from bioinks composed of varying percentages of Alg (2–5.25%), gelatin (2–5.25%), and nanofibrillated cellulose (0.5–1%) at shear rates from 0.1 to 100 s^−1^. The random forest model achieved a coefficient of determination (R^2^) of 0.99 and a mean absolute error (MAE) of 0.09, outperforming other ML models.

Besides viscosity, ML has been applied to elucidate the relationship between printability and other crucial features, such as elastic modulus and yield stress. Kim's group utilized supervised ML, specifically inductive logic programming with the least general generalization algorithm, to model the printability of bioinks formulated from collagen, HA, and fibrin [172]. The study focused on elastic modulus and yield stress, and found that bioinks with high elastic modulus and low yield stress showed good printability for extrusion‐based bioprinting. These properties facilitated smooth extrusion and ensured shape fidelity post‐printing. Multiple regression analysis was subsequently employed to derive a simplified linear model for evaluating printability across various formulations, enabling rapid screening of bioprintable bioinks [172].

ML methodologies, particularly probabilistic approaches like Bayesian optimization, have proven highly effective in optimizing printing parameters to achieve high‐fidelity prints with minimal experimental effort. By modeling the interplay between printing parameters such as air pressure, printing speed, and thickness, ML provides helpful guidance for optimizing the bioprinting conditions for potential bioinks. For instance, Ruberu and coworkers developed a Bayesian optimization‐based ML model to optimize printing parameters for bioinks containing GelMA and HA methacrylate (HAMA) [173]. This ML model leveraged a scoring system to assess filament formation and layer stacking, crucial indicators of printability in extrusion‐based 3D bioprinting. The model enabled the authors to greatly reduce the number of experiments required to identify optimal printing parameters for GelMA‐containing inks with varying concentrations (10%, 7.5% & 5% (w/v)) and for GelMA: HAMA (10:2%, 7.5:2% & 5:2% (w/v)), accelerating the optimization process for different bioink formulations. In another study, Chen and colleagues proposed an AI‐assisted high‐throughput printing condition‐screening system (AI‐HTPCSS), which was composed of a pneumatic extrusion bioprinter and an AI‐assisted image analysis algorithm [174]. This system screened optimal printing parameters, including air pressure, printing speed, and nozzle distance, for Alg‐gelatin bioinks. Images of printed structures were captured and analyzed to assess shape and dimensional accuracy, with the AI algorithm correlating these outcomes to corresponding printing parameters. The trained AI model provided optimal printing conditions based on the designed shape, enhancing the efficiency and reproducibility of the bioprinting process for the same bioprinter and same bioink [174].

### 4.3 Programming of Dynamic Features

The design of bioinks to achieve precise, prescribed shape‐morphing behaviors is a complex challenge, requiring a delicate balance between material responsiveness, structural integrity, and biocompatibility. This process often depends on the expertise of skilled researchers who conduct iterative trial‐and‐error experiments to optimize 3D deformation outcomes, a methodology that is labor‐ and resource‐intensive. To mitigate these limitations, mathematical models [175, 176] and computational simulations, such as finite element analysis (FEA) [36, 177, 178], have been employed to predict and guide shape transformations of smart bioinks, particularly hydrogel‐based systems that undergo swelling or shrinking due to the gain/loss of solvent molecules. FEA has been utilized to simulate the diffusion‐driven shape transformations [179, 180]. It enables researchers to predict how a hydrogel morphs its shape based on computer‐aided design (CAD) patterns, accounting for variables such as crosslinking density, polymer concentration, and environmental stimuli [36, 181, 182]. Additionally, mathematical models, such as the classical Timoshenko theory for bimetallic strips [183], have been adapted to predict the curvature of bilayer hydrogel patterns, while also supporting the inverse design of prescribed shape‐morphing processes [175, 176]. Despite these advancements, the complexity of modeling intricate, non‐linear shape transformations—essential for fabricating many biomimetic constructs—often surpasses the capabilities of traditional computational methods, necessitating the integration of AI‐driven approaches.

**FIGURE 6:**
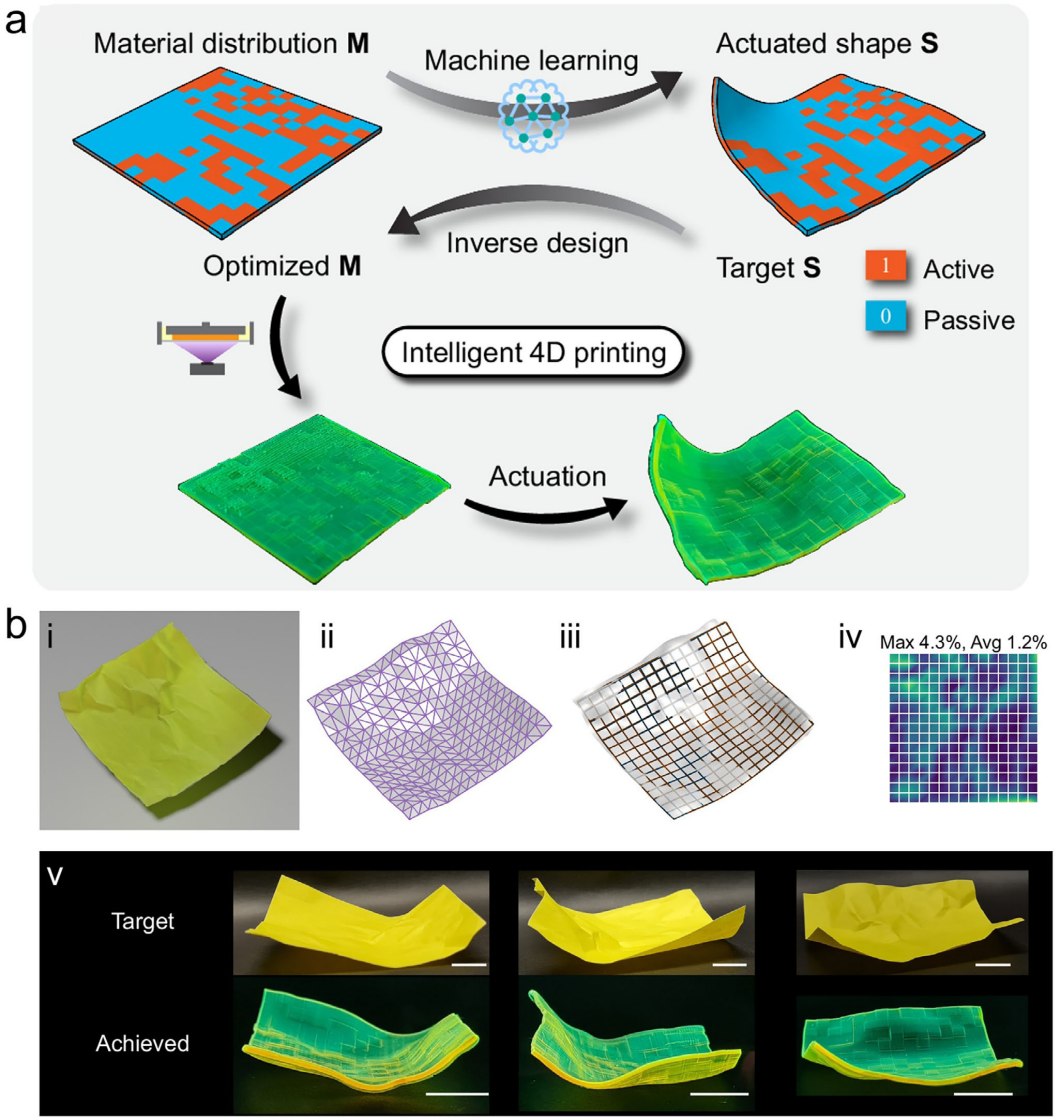
ML‐enabled, voxel‐level inverse design of 4D‐printed active composite plates, representing a promising strategy with potential applications in 4D bioprinting. (a) Schematic illustration of the ML‐driven 4D pattern design based on the given shape. (b) Design result for the scanned target of a crumpled paper, (i) the raw target, (ii) the patch representation, (iii) the optimized shape, (iv) the error map, and (v) the 4D‐printed, actuated shape. (Scale bars: 10 mm). Reproduced with permission [186]. Copyright 2024, Springer Nature.

The applications of AI, particularly ML, enable data‐driven design strategies that predict optimal material distributions and printing parameters, significantly accelerating the inverse design of 4D bioprinting to achieve targeted shape morphing. By leveraging large datasets from experimental measurements or computational simulations, ML models capture the complex, non‐linear relationships between bioink properties, distribution, and morphing behaviors, reducing the need for iterative experimentation. For example, Qi's group presented an approach that combined FEA, ML, gradient descent (GD), and an evolutionary algorithm (EA) to design active composite beams or plates with programmable 3D shape transformations (Figure 6) [184, 185, 186]. Two active composite materials with distinct swelling properties were encoded as “0” and “1” to represent different regions within the construct. FEA‐simulated shape transformations based on specific material distributions generated a comprehensive dataset of morphing outcomes. This dataset was used to train an ML model, which was combined with GD and EA to perform inverse design to identify material distributions yielding targeted 3D shapes. The resulting distributions were converted into grayscale printing slices for grayscale digital light processing (g‐DLP) printing, producing 2D patterns that transformed into complex 3D geometries upon swelling in acetone or water. This high‐fidelity approach demonstrated the power of ML‐driven inverse design to streamline the fabrication of dynamic constructs, offering significant potential for 4D printing adaptive scaffolds or implants [185, 186]. Similarly, Ren and coworkers developed an ML‐driven inverse design strategy for 4D printing of smart micro/nanostructures using the two‐photon polymerization (TPP) technique [187]. Two materials were used for TPP and encoded as “0” and “1”. During TPP fabrication, the “0” and “1” encoded regions were cured by low and high laser power, respectively. This led to variations in material properties, resulting in post‐fabrication shape morphing. FEA simulations predicted the morphing process, generating a dataset that trained a neural network model for forward prediction of shape morphing outcomes. A genetic algorithm, built upon the neural network, performed inverse design to determine optimal material distributions for target shapes, such as arbitrary hand‐drawn lines or a flexible micromanipulator operating within a confined space. The 4D‐printed structures successfully achieved the desired shape transformations under stimuli, highlighting the versatility of ML‐driven inverse design for programming complex, arbitrary shape changes in TPP‐based 4D printing [187].

### 4.4 Mechanical Property Optimization

Smart bioinks should possess appropriate mechanical properties to support the integrity and shape transformation of bioprinted constructs during in vitro/in vivo development. Designing bioinks with mechanical properties that mimic the diverse characteristics of native tissues and organs—ranging from soft neural tissue to stiff bone—presents significant challenges. The mechanical behavior of bioinks is influenced by various factors, such as material composition, molecular structure, crosslinking density, and manufactured architecture (e.g., porosity, pore size, and interconnectivity). Traditional Edisonian approaches rely on iterative experimental cycles to establish the relationships between formulation parameters and mechanical outcomes. Thus, conventional optimization of the mechanical properties of bioinks is time‐consuming and resource‐intensive. As a result, the inverse design of bioinks—where formulations are tailored to achieve specific mechanical properties—has been a complex task. The emergence and applications of ML have accelerated this process by enabling data‐driven inverse design strategies that predict and optimize bioink formulations and architectures based on prescribed mechanical properties [165, 188]. Hydrogels, particularly natural hydrogels, are widely used in bioinks due to their excellent printability and biocompatibility. Deep learning, an ML approach, has been used to predict the relationship between the mesoscopic network structure and macroscopic mechanical properties of hydrogels [189]. By constructing mesoscopic network structural models using the self‐avoiding walk method, two ML algorithms, including deep neural networks and 3D convolutional neural networks, were employed to map these structures to macroscopic mechanical properties. These ML models successfully predicted stress–stretch curves of hydrogels under uniaxial tension, offering insights into their elasticity and strength. Additionally, ML has been applied to predict the mechanical properties of composite bioinks, which incorporated bioglass to enhance the stiffness or toughness of collagen [190]. An FEA‐based ML model was developed to predict the Young's modulus and Poisson's ratio of bioglass‐collagen composite hydrogels. By generating 2000 microstructural images with varying bioglass particle distributions and using FEA simulations to calculate the mechanical properties, a convolutional neural network regression model was trained to achieve high predictive accuracy.

Living cells are an essential component of bioinks, and their presence in hydrogels greatly affects their mechanical properties. However, few studies have systemically investigated the impact of cells on the mechanical properties of bioinks due to the high costs and long processing time required for both cell culture and bioprinting. To address the limitation of costly bioinks, Guan and colleagues introduced a neural network‐based ML technique and combined it with transfer learning to predict the stiffness of cell‐laden hydrogel scaffolds produced via DLP [191]. They first generated the stiffness dataset using a cost‐effective 293T cell line by using different cell densities and light exposure durations while maintaining other parameters such as GelMA concentration, photoinitiator concentration, and light intensity. The resulting dataset was then used to train the ML model with varying inputs, including cell density, exposure time, and measured stiffness. To extend the model's applicability to other costly cell types, transfer learning was employed to predict the stiffness of scaffolds containing HepG2 hepatoma cells using a significantly reduced dataset. Transfer learning enabled the adaptation of the ML model originally trained with 293T cells to predict the stiffness of HepG2 cell‐containing scaffolds, thereby greatly reducing the required experimental data [191]. Such ML‐driven strategies hold promise for scaling up the design of smart bioinks, enabling the production of 4D bioprinted constructs with precise mechanical characteristics for applications in tissue engineering and regenerative medicine.

In addition to the intrinsic material properties of bioinks, the manufactured shape and architectural features, such as porosity, pore size, and interconnectivity, also significantly influence the mechanical behaviors of bioprinted constructs. The architecture of the printed construct must support both initial structural stability and subsequent dynamic transformations. Some simulation approaches, such as FEA, have been used to investigate the force distribution and predict the mechanical behavior according to the designed architectures [192], thereby assisting researchers in optimizing their mechanical performance. Moreover, ML, combined with advanced bioprinting techniques, has further accelerated the inverse design of structures based on prescribed mechanical properties, effectively bypassing traditional iterative design‐manufacturing cycles. For instance, Ha and colleagues reported an inverse design methodology that used generative ML and an SLA 3D printer to create a series of uniaxial compressive stress‒strain curves while accounting for process‐dependent errors from printing [193]. Customized mechanical behaviors were achieved with nearly 90% fidelity between the targeted and experimentally measured results. This high level of accuracy, accomplished without iterative design‐manufacturing cycles, contributed to the inverse design of materials with prescribed mechanical properties.

### 4.5 Degradation Optimization

Degradation kinetics are a crucial consideration in the design of smart bioinks for 4D bioprinting. The resulting constructs must degrade at a rate that supports cellular development both in vitro and in vivo, ideally in synchrony with the ingrowth of newly forming tissue [194]. Recent advances in ML have enabled data‐driven prediction and optimization of hydrogel degradation behaviors, offering transformative potential for tailoring bioink degradation. For instance, Entekhabi and colleagues explored the degradation behavior of genipin‐crosslinked gelatin scaffolds using ML techniques [195]. Genipin, as a crosslinker, enhanced the mechanical properties of gelatin while modulating its degradation rate. Biodegradable porous scaffolds were fabricated with varying weight percentages of gelatin (2.5–10% w/v) and genipin (0–1.0% w/v), and their degradation rates were experimentally determined in phosphate‐buffered saline (PBS, pH 7.4) at 37 °C over 28 days. The resulting dataset was used to train two ML models: an artificial neural network (ANN) and kernel ridge regression (KRR). The ANN model outperformed KRR, achieving a mean squared error of 2.68% compared to 4.78% for KRR, demonstrating superior predictive accuracy. In another study, Robles‐Bykbaev and coworkers developed a method combining artificial vision and ML to optimize the analysis of collagen type I scaffold degradation in bone regeneration systems [196]. A random forest classifier was used to segment optical microscopy images, accurately identifying regions of interest, such as collagen, ECM, nuclei, and background, based on texture features like structure tensor and entropy. This ML‐driven segmentation achieved correct classification of 97.3% of pixels, enabling precise quantification of collagen degradation and osteocyte growth. Finally, the parametric logistic mixture models were utilized to model and predict collagen degradation resulting from the biological activities [196]. Overall, these studies demonstrated the capacity of ML to decode complex degradation mechanisms and accelerate the development of bioinks with programmable, tissue‐specific bioresorption rates—a prerequisite for 4D bioprinted systems intended to recapitulate native tissue remodeling processes.

### 4.6 Cell Viability Optimization

Cell viability is a critical determinant of successful 4D bioprinting, where living cells encapsulated within bioinks must survive the bioprinting process and grow well in dynamic, stimuli‐responsive constructs. Predicting and optimizing cell viability remain challenging due to the interplay between multiple factors, such as cell type, rheological properties (related to shear rate and shear stress), and hydrogel crosslinking mechanisms (the use of UV or near‐UV light). These variables influence cell survival during bioprinting and subsequent shape transformations, necessitating advanced strategies to enhance biocompatibility. ML has been integrated with bioprinting technologies to accelerate the optimization of cell viability in smart bioinks.

In extrusion‐based bioprinting, living cells are often subjected to high shear stresses when passing through a nozzle, which can compromise their viability. ML approaches have been employed to model and predict cell viability as a function of these stresses. For instance, a study utilized multi‐layer perceptron (MLP) regressors to predict the viability of various cell types exposed to shear stress for different durations [72]. The MLP models were trained with data from human vascular endothelial cells, MSCs, cervical cancer cells, and embryonic fibroblasts, capturing the effects of different shear stress (1.5–5.0 kPa) and exposure durations (100–700 ms). The results revealed distinct viability thresholds: the viability of vascular endothelial cells was reduced to 80% at 2.05 kPa for 400 ms, while MSCs, cervical cancer cells, and embryonic fibroblasts reached this viability threshold at 2.65, 2.85, and 3.72 kPa, respectively. These findings showed great potential of ML in optimizing the viability of various cell types in bioinks [72].

Cryogenic bioprinting, which combines extrusion‐based bioprinting with cryopreservation, has become increasingly popular for its ability to print low‐viscosity bioinks at low temperatures, thereby enhancing shape fidelity and enabling the fabrication of complex constructs [197, 198, 199, 200]. In addition, cryogenic bioprinting holds promising potential for producing off‐the‐shelf living tissues. However, low‐temperature environments pose significant challenges to cell survival, necessitating the use of cryoprotective agents (CPAs) to mitigate permeable shock and ice crystal formation. The properties of CPA‐containing bioinks and their impact on cell viability are complex and difficult to predict using conventional methods, as the relationships between CPA type, concentration, and cellular response are highly non‐linear. To solve this problem, Qiao and coworkers utilized an ML approach to predict the effects of CPAs on the GelMA bioinks [201]. Two datasets were generated by investigating the viability of cells embedded within GelMA bioinks containing two commonly used CPAs, ethylene glycol and glycerol. Four ML models—multiple linear regression, decision tree, random forest, and ANN—were trained on these datasets. The ANN model demonstrated superior performance, achieving a coefficient of determination (R^2^) of 0.96 and a mean absolute error (MAE) of 0.08. Therefore, this model was subsequently used to predict the effects of additional CPAs on cell viability and bioink properties. This approach accelerated the development of cryoprotective bioinks by identifying optimal CPA formulations that maximized cell survival while maintaining printability and shape fidelity [201].

## 5 Biomaterials for 4D Bioprinting

**FIGURE 7:**
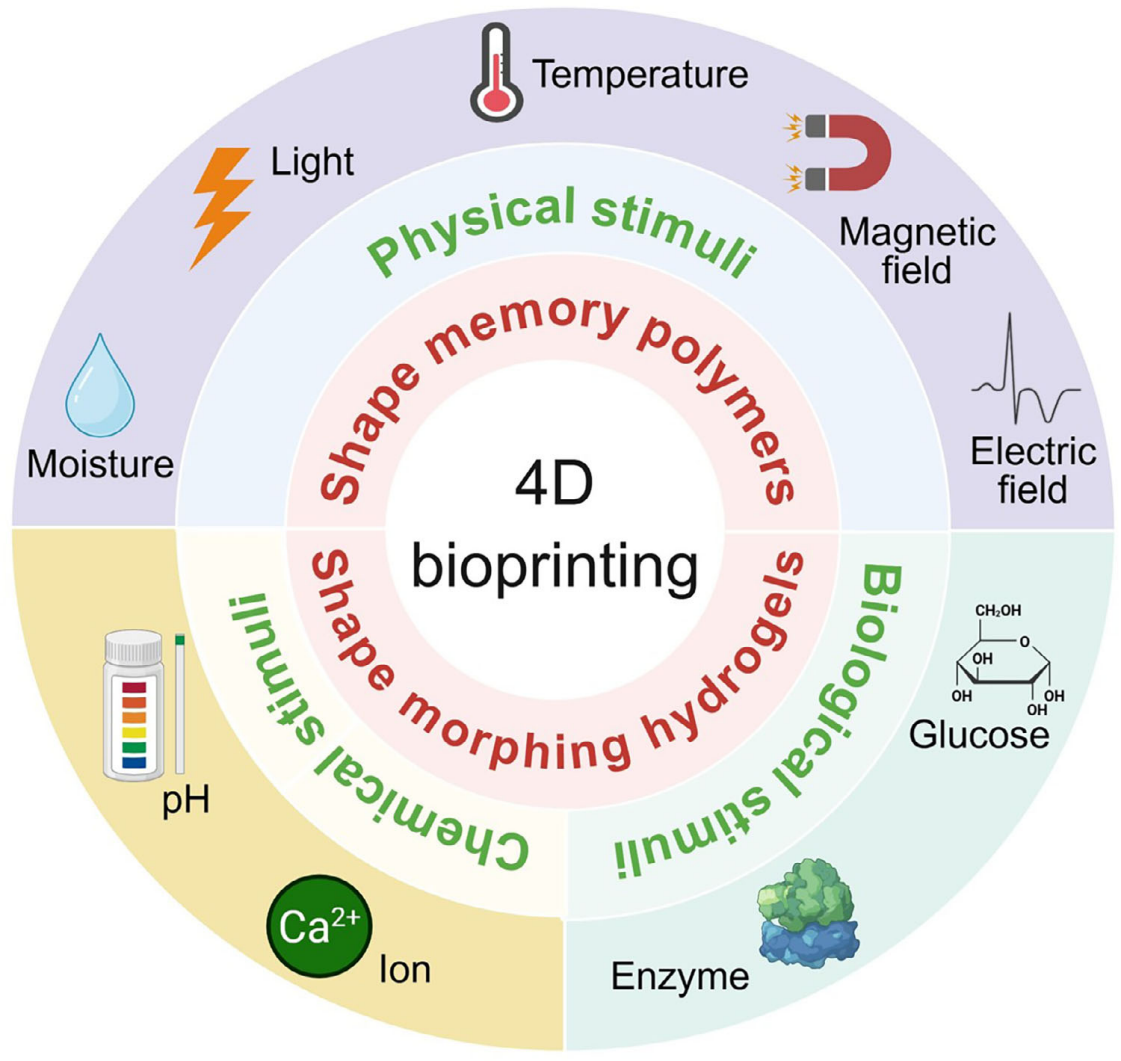
Stimuli‐responsive smart bioinks including SMPs, SMHs, and their composites undergo programmed transformations in response to physical, chemical, or biological triggers for 4D bioprinting applications. Created with BioRender.com.

Biomaterials serve as the fundamental components of smart bioinks for 4D bioprinting. Currently, SMPs, SMHs, and their composites are the most widely used biomaterials for smart bioinks. These materials can undergo shape morphing or functional transformation in response to various internal or external stimuli (Figure 7), as discussed in this section. Given that 4D bioprinting is still in its early developmental stage, we will also explore biomaterials that have been successfully used in conventional 4D printing applications and demonstrated strong potential for adaptation to 4D bioprinting systems (Table 3). Additionally, we will discuss those biomaterials that have been used for hybrid 4D biofabrication. Furthermore, prospective smart biomaterials that have great potential for future applications are emphasized.

**TABLE 3:** Summary of biomaterials for 4D printing and 4D bioprinting.

Biomaterials	Category	Printing technologies	Mechanisms	Cell type	Reversibility	Response time	Validation level	Principal limitations	Applications	Refs.
PNIPAM/Alg	4D bioprinting	Extrusion‐based	Temperature‐triggered shape morphing	EA.hy926 cells	Yes	Minutes	In vitro	Poor mechanical strength	Vasculature	[211]
PLATMC/GelMA multilayer	Hybrid 4D biofabrication	Extrusion‐based	Temperature‐triggered shape morphing	BMSCs	No	Minutes	In vitro	Acellular substrate; complex fabrication	Uterine tissue	[40, 41]
PEG bilayers	4D bioprinting	SLA	Differential swelling of PEG bilayers	β‐TC‐6 cells	No	Hours	In vitro	Limited complexity	Vasculature	[221]
Sil‐MA bilayer	4D bioprinting	DLP	Anisotropic volume change of Sil‐MA bilayer	Turbinate‐derived MSCs	No	Hours	In vivo	Slow response	Trachea	[181]
Oxidized and methacrylated Alg/GelMA bilayer	4D bioprinting	Extrusion‐based	Differential swelling of bilayers	hMSCs	No	Hours	In vitro	Slow kinetics	Cartilage‐like tissue	[204]
Oxidized and methacrylated Alg/GelMA/8‐arm PEG‐acrylate	4D bioprinting	Extrusion‐based	Different swelling of hydrogel layers with crosslinking gradient	MSCs	No	Hours	In vitro	Gradient control challenges	Bone	[220]
GelMA/PEGDA	4D bioprinting	DLP	Different swelling of hydrogel layers with crosslinking gradient	NIH3T3	No	Hours	In vitro	UV exposure risk	Advanced 4D bioprinting	[42]
Gel/GelMA/MXene	4D bioprinting	Extrusion‐based	Different swelling of hydrogel layers with crosslinking gradient	PC12 and HUVECs	No	Hours	In vitro	Nanoparticles toxicity unknown	Advanced 4D bioprinting	[205]
Epoxy‐based SMPs	4D printing	SLA	NIR laser‐triggered shape morphing	NSCs	No	Seconds	In vitro	No cell encapsulation	Brain	[229]
Fe_3_O_4_@CS/GelMA	4D bioprinting	Extrusion‐based	Magnetic field‐triggered functional transformation	Chondrocyte	Yes	Seconds	In vivo	Nanoparticle bioaccumulation	Cartilage	[235]
Methacrylated decellularized ECM	4D bioprinting	Extrusion‐based	Electrical field‐triggered functional transformation	hASCs	No	Continuous	In vitro	Complex setup	Muscle	[237]
GelMA	4D bioprinting	Extrusion‐based	Electrical field‐triggered functional transformation	C2C12 cells	No	Continuous	In vitro	Limited to electroactive tissues	Muscle	[238]
PAA/GelMA/PEGDA	4D bioprinting	Extrusion‐based	pH‐triggered swelling	hMSCs	Yes	Minutes	In vitro	Non‐physiological pH range	Advanced 4D printing	[242]
Oxidized and methacrylated Alg/GelMA bilayer	4D bioprinting	Extrusion‐based	pH‐triggered swelling of bilayer gradient Alg‑GelMA hydrogel	Fibroblasts and hASCs	Yes	Hours	In vitro	Non‐physiological pH range	Cartilage/bone	[243]
OMA jammed micro‐flake hydrogel (MFH) system	4D bioprinting	Extrusion‐based	pH‐triggered swelling	NIH3T3	Yes	Hours	In vitro	Non‐physiological pH range	Cartilage‐like tissue	[244]
Alg‑gelatin with pre‑crosslinking Ca^2^^+^/Co^2^^+^ + Gelatin (thermosensitive)	4D bioprinting	Extrusion‐based	Ion‐triggered crosslinking and thermosensitive component	cell spheroid or single cells	No	Minutes	In vitro	Heavy metal ion cytotoxicity	4D extrusion bioprinting	[247]
Alg/HA	4D bioprinting	Extrusion‐based	Ion‐triggered swelling	BMSCs	No	Hours	In vitro	Poor mechanical strength	Vasculature	[22]
Interparticle‐crosslinked zwitterionic microgels	4D bioprinting	Extrusion‐based	Ion‐triggered (Hofmeister effect‐driven ionic response: contraction/expansion behaviors)	Human primary chondrocytes	Yes	Minutes	In vitro	Complex formulation	Multi‐material 4D bioprinting	[248]
GOx/HRP glucose‐mediated Alg‑Ph ink	4D bioprinting	Extrusion‐based	Glucose‐triggered (GOx catalyzes the production of H_2_O_2_ from glucose, triggering HRP crosslinking)	Mouse 10T1/2 fibroblasts	No	Minutes	In vitro	Limited to crosslinking	4D bioprinting	[206]
Gelatin‑norbornene‐hydroxyphenylacetic acid (GelNB‑HPA)/HA hybrid gel	Prospective smart materials	Not applicable	Enzyme triggered (Tyrosinase‐triggered crosslinking/stiffening)	COLO‑357 (PDAC)	No	Hours	In vitro	No cell encapsulation	Enzyme‐triggered stiffening mimic ECM remodeling	[207]
Hyaluronan + peptide folding‐mediated dynamic hydrogel	Prospective smart materials	Extrusion‐based	Enzyme triggered crosslinking (Specific peptide folding regulates peptide‐polymer crosslinking for enzyme‐mediated biomineralization control)	HepG2	Yes	Hours	In vitro	No cell encapsulation	Dynamic modulation of printed structure	[257]

### 5.1 Technological Readiness Classification

To facilitate evaluation of the translational potential of the smart biomaterials and bioprinting strategies discussed in this review, we introduce a hierarchical classification system that distinguishes technologies based on their maturity and cellular integration level. The 4D printing and bioprinting technologies are categorized into four progressive readiness levels. The first level is acellular 4D printing, an established technology involving stimuli‐responsive materials without living cells. These systems are the most mature, with well‐characterized SMPs and SMHs that have demonstrated reproducible performance under controlled conditions [202, 203]. The second level is hybrid 4D biofabrication, which encompasses merging approaches that combine acellular stimuli‐responsive substrates with cell‐laden hydrogel layers or biological components. These systems leverage the reliable mechanical performance of synthetic SMPs while providing a biocompatible environment for cells [40, 41]. In hybrid strategies, the shape‐morphing function is typically driven by the acellular component, while the cellular component provides biological activity. The third level is 4D bioprinting, which refers to the fabrication of cell‐laden constructs entirely composed of stimuli‐responsive hydrogels or smart bioinks that undergo shape or functional transformation [204, 205]. These systems represent the most biologically integrated approach, with cells directly participating in or responding to the dynamic environment. The fourth level is prospective smart materials, which are biomaterials and bioink formulations with demonstrated stimuli‐responsive properties in acellular systems or promising biocompatibility profiles, but lacking validation in 4D bioprinting contexts. These materials represent the frontier of the field, offering new mechanisms of responsiveness (e.g., enzyme‐triggered, glucose‐sensitive) that could address limitations of current systems [206, 207].

### 5.2 Physical Stimuli‐Responsive Biomaterials

#### 5.2.1 Temperature‐Responsive Biomaterials

Temperature is one of the most accessible and precisely controllable physical stimuli in biomedical environments, making it especially effective for eliciting responsive behaviors in smart bioinks. Within the framework of 4D bioprinting, temperature‐responsive biomaterials provide a unique opportunity to develop constructs capable of dynamically altering their structures or functionalities post‐fabrication. These biomaterials are engineered to undergo predictable and reversible structural or functional transformations—such as sol–gel transitions, expansion, contraction, or phase separation—within a relatively narrow and often physiologically relevant temperature window, typically ranging from 30°C to 37°C. Such responsiveness is essential for realizing various biomedical applications, such as minimally invasive delivery systems, shape‐adaptive implants, dynamic tissue scaffolds, and stimulus‐responsive drug delivery platforms.

**FIGURE 8:**
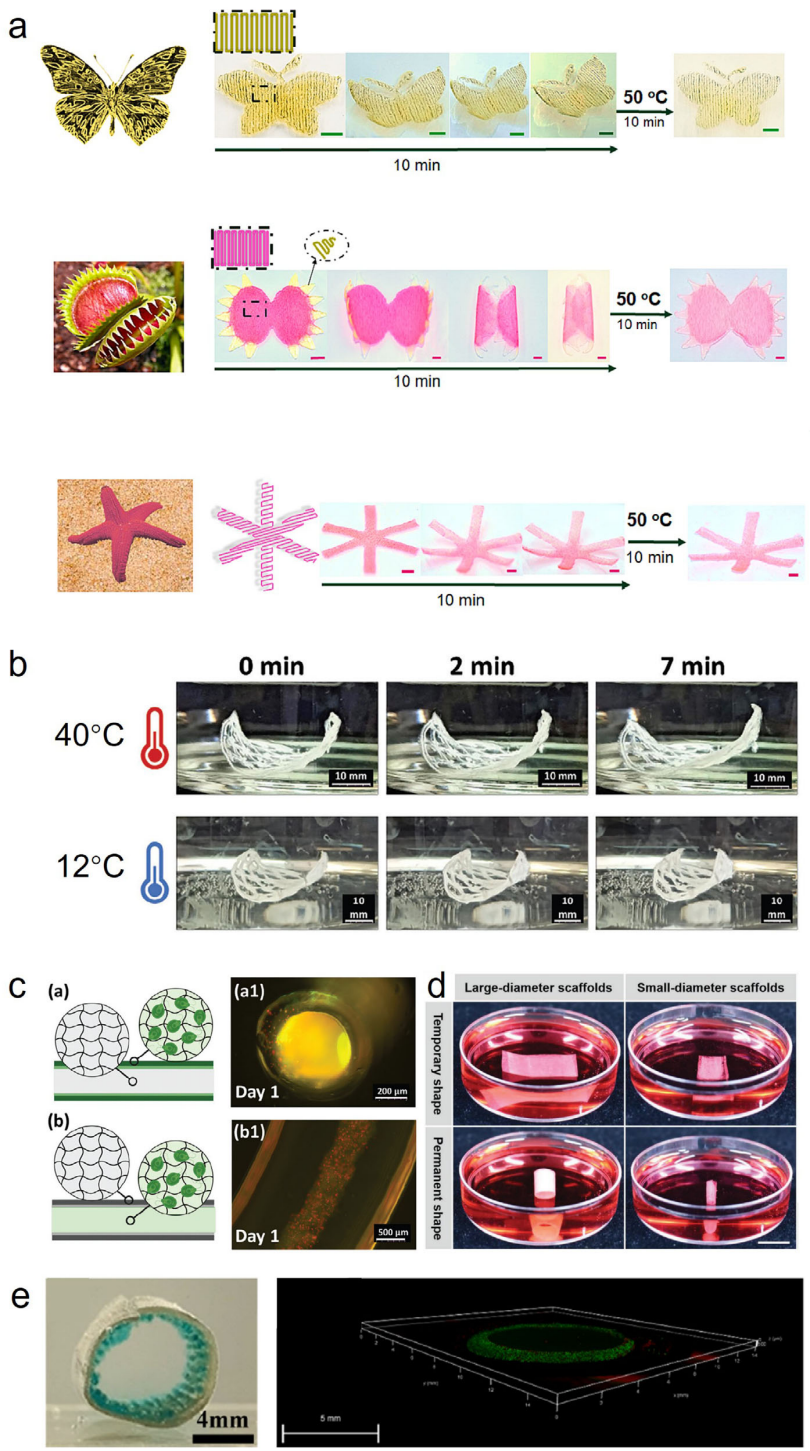
Temperature‐responsive biomaterials for 4D printing/bioprinting. (a) Printed biomimetic anisotropic PNIPAM hydrogel patterns (i.e., butterfly‐like, flytrap‐like, and starfish‐like ones) underwent shape morphing at 50°C in water. Reproduced with permission [210]. Copyright 2023, Elsevier. (b) Shape‐morphing process of printed PLATMC/MXene hydrogels at different temperatures (12°C and 40°C). (c) Microalgae‐assisted visualization of coaxially 4D‐printed microtubes. Reproduced with permission [211]. Copyright 2023, Wiley. (d) Shape‐morphing process of PLATMC scaffolds to form tubes with different diameters after being immersed in culture medium at 37°C. Reproduced with permission [212]. Copyright 2018, Wiley. (e) Multi‐layer scaffolds using PLATMC as the substrate to realize hybrid 4D biofabrication. Reproduced with permission [41]. Copyright 2024, American Chemical Society.

Many SMPs such as PLA, PNIPAM, and poly‐(lactic acid‐co‐trimethylene carbonate) (PLATMC) are typical temperature‐responsive biomaterials and have been widely used for 4D printing [208, 209]. Among them, PNIPAM stands out as it exhibits a sharp lower critical solution temperature (LCST) at approximately 32°C, which is close to body temperature. Below the LCST, PNIPAM exists in a hydrated, expanded state, whereas above this temperature, it undergoes a rapid and reversible collapse into a hydrophobic, compact form. This distinct and predictable transition makes PNIPAM highly suitable for biomedical applications, where an external thermal cue can trigger a functional response, such as shape change or controlled release [203]. For example, Li and colleagues fabricated nature‐inspired anisotropic hydrogel actuators by 4D printing temperature‐responsive composites of short carbon fibers (SCFs) and PNIPAM [210]. Upon stimulation by temperature, the hydrogels had unbalanced swelling and shrinking, leading to reversible biomimetic shape morphing. For example, butterfly‐like, flytrap‐like, and starfish‐like hydrogel actuators could be constructed, and these actuators showed reversible shape morphing when immersed in 25°C and 50°C water (Figure 8a). Although SCFs/PNIPAM inks could be employed to encapsulate cells for 4D bioprinting, PNIPAM has suboptimal mechanical strength and limited biocompatibility. Thus, PNIPAM is frequently copolymerized with natural polymers like gelatin, HA, and Alg to achieve better biological integration and tunable viscoelastic properties. For example, Podstawczyk and colleagues used PNIPAM/Alg bioinks to fabricate microtubes via coaxial 4D bioprinting for vascular tissue engineering [211]. PNIPAM renders the microtube temperature‐sensitive and endows it with a shape‐morphing property (Figure 8b). Microalgae *Chlamydomonas reinhardtii* can be printed in either the core or shell part of the microtubes while maintaining high viability, demonstrating the good biocompatibility of PNIPAM/Alg bioinks (Figure 8c).

The thermoresponsive shape‐memory behavior of PLA enables precise programmed shape changes in 4D printed constructs heated above its glass transition temperature (Tg, approximately 60°C). However, due to the relatively high Tg of PLA, which exceeds physiological temperatures, its application in 4D bioprinting is limited. Therefore, PLA is often modified to lower its Tg. One example is the development of PLATMC. The Tg of PLATMC can be precisely adjusted by controlling the LA:TMC ratio. Zhao and colleagues demonstrated the 4D transformation of a PLATMC membrane through programmed shape morphing upon immersion in a 37°C culture medium [212]. With a suitable Tg of PLATMC, the shape‐memory PLATMC tubes were able to retain their temporary planar shapes at room temperature (25 °C), and completely recovered their permanent tubular structures at physiological temperature (37°C) due to the glass transition of PLATMC (Figure 8d). Wang and coworkers also employed PLATMC/MXene inks to fabricate artificial nerve guidance conduits (NGCs) to enhance peripheral nerve injury regeneration [213]. Upon stimulation by body temperature, the printed sheet‐like PLATMC/MXene structure could quickly self‐roll into a conduit‐like structure. Although PLATMC demonstrates excellent temperature responsiveness and holds significant potential for 4D printing applications, it is infeasible to utilize PLATMC ink for cell encapsulation in hybrid 4D biofabrication. To address this limitation, Chen and colleagues proposed a novel strategy of bioprinting cell‐laden hydrogels on a substrate of 3D‐printed PLATMC membrane [40, 41]. As shown in Figure 8e, after being cultured in medium at 37°C, the planar multi‐layer constructs transformed to a tubular structure suitable for uterine regeneration, ultimately realizing hybrid 4D biofabrication [41].

Although many other temperature‐responsive biomaterials, including poly(acrylic acid) (PAA), poly(glycerol sebacate) acrylate‐co‐hydroxyethyl methacrylate (PGSA‐HEMA), and poly(glycerol dodecanoate) acrylate (PGDA), have been demonstrated to be effective for 4D printing, the applications of these biomaterials for cell encapsulation remain challenging [214, 215, 216]. Recent studies have also indicated the potential of 4D printing in ceramics [178]. Thus, flat structures of hydrogel‐ceramic composites could transform into complex 3D shapes through dehydration and ultimately become solid ceramics after sintering. However, there is still a long way to go before achieving the complexity and functionality associated with 4D bioprinting of such materials.

While temperature is one of the most accessible and precisely controllable physical stimuli, its application in 4D bioprinting must be carefully calibrated to ensure cell viability and in vivo safety. For cell‐laden constructs, the actuation temperature should ideally fall within physiological limits (30°C–42°C) to maintain high cell viability (>85%) and prevent thermal stress or cell apoptosis. Temperatures exceeding 42°C can induce heat shock responses, protein denaturation, and cell death, while temperatures above 45°C are generally considered cytotoxic for most mammalian cells. Similarly, for in vivo applications, localized temperature increases must be carefully controlled to avoid thermal damage to surrounding healthy tissues. Therefore, thermoresponsive materials designed for 4D bioprinting should ideally exhibit transition temperatures within this biocompatible window [202]. Moreover, for light‐, magnetic‐, and electric field‐responsive biomaterials that involve the generation of localized heating for 4D actuation, the thermal conversion efficiency should be carefully considered to ensure that the transition temperatures are within the biocompatible window.

#### 5.2.2 Moisture‐Responsive Biomaterials

Moisture‐responsive biomaterials offer a biologically inspired platform for smart bioinks in 4D bioprinting, utilizing water, a ubiquitous and biocompatible stimulus in physiological environments, to drive controlled shape or functional transformations [217]. These materials exhibit dynamic, reversible changes in shape, stiffness, or volume upon exposure to humidity or aqueous media, making them particularly suitable for biomedical applications characterized by the presence of water (e.g., repair of skin, mucosal tissues, and internal organs). Their responsiveness primarily stems from hygroscopic swelling mechanisms: water molecules diffuse into the polymer network, interacting with hydrophilic functional groups to induce volumetric expansion, softening, and macroscopic shape changes. Naturally derived polymers such as Alg, HA, gelatin, and cellulose derivatives are particularly promising due to their exceptional water‐absorption capacities. These materials often can absorb water up to several times their dry weight and undergo pronounced hydration‐induced alterations in mechanical properties and microstructure. By strategically modulating polymer chemistry, molecular weight, and crosslinking density, these transformations can be precisely engineered to meet specific 4D bioprinting requirements, enabling applications such as self‐bending wound dressings, dynamic tissue scaffolds, and responsive drug delivery systems [218, 219].

**FIGURE 9:**
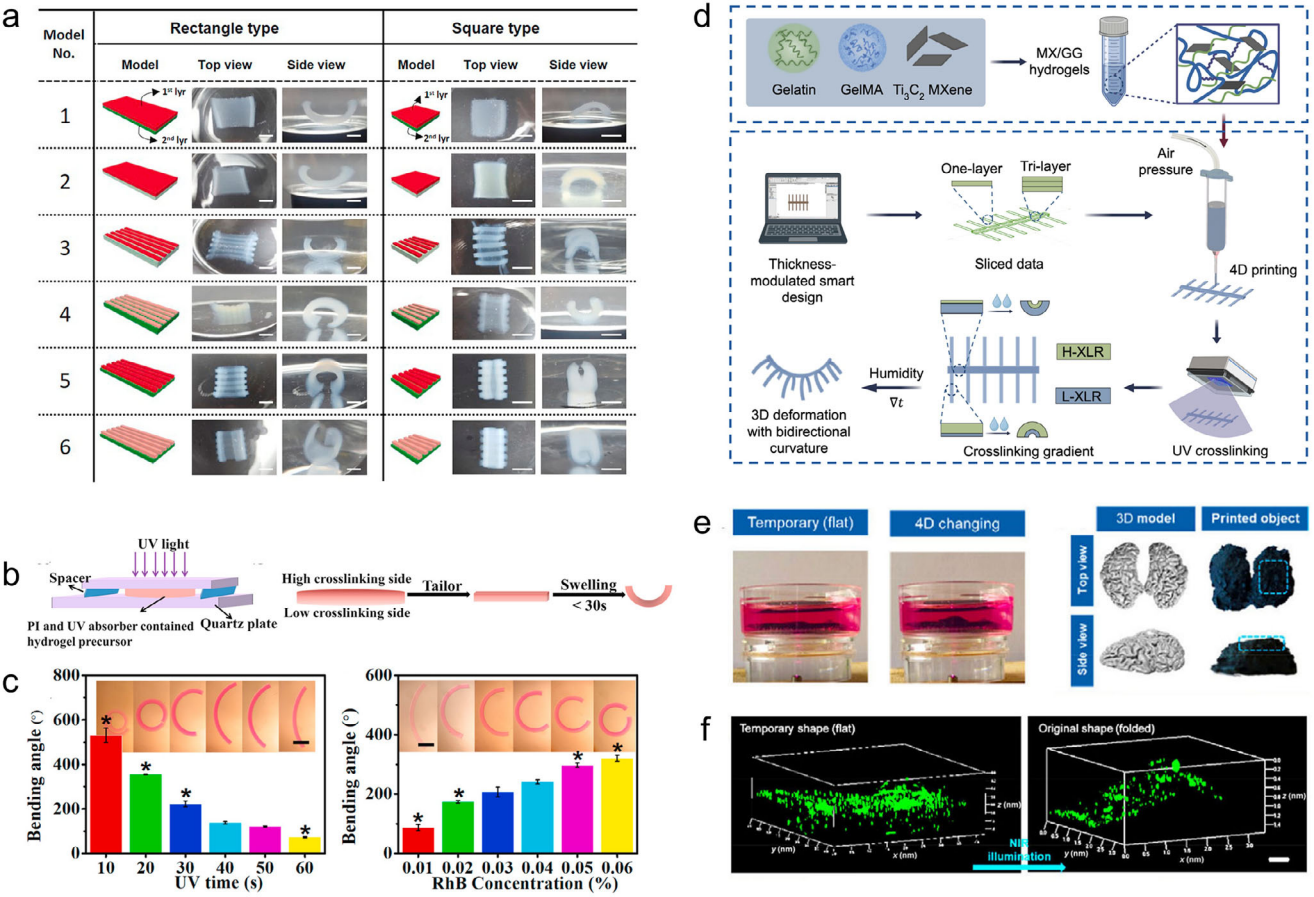
Moisture‐ and light‐responsive biomaterials for 4D printing/bioprinting. (a) Shape‐morphing Sil‐MA hydrogel sheets with rectangle and square shapes. Dark red or dark green: high‐concentration Sil‐MA (30%), light red or light green: low‐concentration Sil‐MA (15%). Scale bars, 500 µm. Reproduced with permission [181]. Copyright 2020, Elsevier. (b) Schematic illustration showing the fabrication of a hydrogel with crosslinking density gradient. (c) Bending degree of hydrogels as a function of UV crosslinking time and photo absorber concentration. Reproduced with permission [220]. Copyright 2022, Elsevier. (d) Schematic illustration of 4D bioprinting of cell‐laden MX/GG hydrogels with programmable, moisture‐responsive 3D deformation. H‐XLR: high crosslinking region; L‐XLR: low crosslinking region. Reproduced with permission [205]. Copyright 2022, Wiley. (e) 4D transformation of brain constructs from a temporary flat shape to the original folded shape in the culture medium when exposed to NIR illumination. (f) Neural stem cells (NSCs) on 4D printed brain constructs before (in the flat shape) and after shape morphing (in the folded shape). The scale bar is 500 µm. Reproduced with permission [229]. Copyright 2019, Springer Nature.

Currently, the most widely adopted 4D bioprinting strategy involves the fabrication of multi‐layered scaffolds [204, 220]. Shape morphing occurs as a result of the varying swelling ratios for the different layers. For example, Jamal and colleagues developed photopatterned PEG‐based hydrogel bilayers capable of shape‐morphing to form curved structures [221]. In this study, the difference in crosslinking density between the top and bottom layers of PEG resulted in distinct swelling behaviors in culture medium, thereby inducing the self‐folding of β‐TC‐6 cell‐laden hydrogels. Additionally, Kim and coworkers used photocurable silk fibroin (Sil‐MA) as bioinks to prepare bilayered hydrogels via the DLP bioprinting technique for damaged trachea regeneration [181]. By modulating the printing patterns of two layers, bilayered Sil‐MA hydrogels underwent shape morphing due to the anisotropic volume change in aqueous environments (Figure 9a). Ding and colleagues employed a 4D cell‐condensate bioprinting approach to construct a shape‐morphing bilayer system [204]. This bilayer system comprised a preformed hydrogel layer with a crosslinking gradient and a printed photocurable and degradable microgel (MG) layer. The former functioned as the actuation layer responsible for driving shape transformation, and the latter enabled the deposition of a cell‐only bioink and maintained the structural integrity of the printed cellular architecture.

Another feasible approach to achieving moisture‐responsive shape changes is to establish a gradient in crosslinking density throughout the entire structure. Figure 9b shows the formation of a cell‐laden gradient hydrogel with pre‐programmable deformation. It can be fabricated by simply photocrosslinking a homogeneous mixture of a photocrosslinkable polymer macromer, a photoinitiator, a UV absorber, and live cells [220]. Due to the presence of the UV absorber, the top layer of the hydrogel becomes highly crosslinked, whereas the bottom layer remains relatively lightly crosslinked. After being cultured in the medium, the resulting swelling difference between the layers induced shape transformation. By adjusting the concentration of the UV absorber, the thickness of the hydrogel, and the duration of UV crosslinking, the shape morphing behavior of the hydrogel could be precisely regulated (Figure 9c). Similarly, Bharath and coworkers developed a 4D bioprinting system using GelMA, PEGDA, and a photoabsorber as the bioink [42]. With a crosslinking gradient caused by photoabsorber‐induced light attenuation, the bioprinted constructs exhibited structural anisotropy, enabling rapid shape deformation when cultured in a 37°C medium. Furthermore, we recently employed MXene as an effective photo‐absorber to develop gelatin/GelMA bioinks for 4D bioprinting [205]. As shown in Figure 9d, MX/GG hydrogels with distinct spatial crosslinking gradients were fabricated by simply adjusting the domain‐specific pattern thickness and a single UV exposure. FEA simulations were utilized to effectively guide the thickness‐controlled shape‐morphing process.

#### 5.2.3 Light‐Responsive Biomaterials

Light serves as a particularly attractive external stimulus for 4D bioprinting, and light‐responsive biomaterials can provide precise spatial and temporal control over biological and structural transformations [222]. Light can be applied remotely and non‐invasively. Moreover, its intensity, wavelength, duration, and location can be modulated to guide cellular behavior, regulate biochemical signaling, and induce mechanical changes within engineered constructs at micrometer or even sub‐micrometer resolution.

One of the key advantages of utilizing light in biomedical applications is its tunability across a range of wavelengths. Near‐infrared (NIR) light, generally spanning 700 to 1100 nm, presents distinct advantages for biomedical applications. This is attributed to its ability to penetrate tissues while being minimally absorbed by water and hemoglobin. Although penetration depths of several centimeters have been documented in highly transparent tissues, the actual penetration of NIR light is highly tissue‐specific and notably diminished in vascularized or pigmented tissues. In comparison with UV or visible light, NIR light experiences less scattering and absorption. As a consequence, it exhibits lower phototoxicity and allows for deeper tissue penetration in numerous applications. These features have promoted the development of NIR‐responsive biomaterials that can be selectively activated through external light exposure [223, 224].

Smart bioinks composed of light‐responsive systems are typically categorized based on whether their functional transformations are driven by photochemical or photothermal processes. Photochemical systems operate through light‐induced molecular changes that alter the material's properties. For example, spiropyran, a photochromic molecule, undergoes reversible isomerization into a more polar merocyanine form upon exposure to UV light [225]. This change in polarity affects the hydrophilicity of the surrounding polymer matrix, thereby inducing either volume expansion or contraction, depending on environmental conditions. Another photochemical strategy involves incorporating o‐nitrobenzyl groups into polymer chains. When exposed to light, these groups undergo cleavage, resulting in the de‐crosslinking of the polymer network. This mechanism enables controlled degradation, release of encapsulated cargo, or softening of the material, making it suitable for applications requiring temporary scaffolds or tunable release profiles [226].

In comparison, smart bioinks based on photothermal systems are more preferable for 4D bioprinting. Photothermal systems typically utilize nanomaterials that can efficiently convert absorbed light into heat. Commonly used photothermal nanoparticles include gold nanorods (AuNRs), graphene and graphene oxide sheets, and conductive polymers such as polypyrrole. Upon irradiation with NIR light, these nanoparticles generate localized heat, which can induce a secondary response in adjacent thermosensitive materials. For example, Wang and coworkers incorporated black phosphorus nanosheets into 4D‐printed β‐TCP/PLATMC composite scaffolds to treat irregularly shaped critical‐size bone defects [227]. When the composite scaffold was exposed to on‐demand irradiation with an 808 nm near‐infrared (NIR) laser, its temperature rapidly rose to 45 °C, allowing the scaffold to undergo shape reconfiguration. This enabled easier implantation and precise adaptation to irregular bone defects. Sheikhi and colleagues 4D printed polydopamine‐functionalized granular hydrogels for application in soft robotics [228]. Upon exposure to irradiation at an intensity of 1000 mW·cm^−^^2^ for 2 minutes, the internal temperature of the hydrogels increased to 38.2–43.0°C, which melted the crystalline domains and consequently induced shape morphing. As shown in Figure 9e,f, a 4D‐printed proof‐of‐concept brain model was developed using an NIR light‐responsive nanocomposite [229]. In this study, epoxy was chemically modified to synthesize an SMP with a Tg of approximately 45 °C. The incorporation of graphene enabled the printed brain model to exhibit shape‐morphing behavior upon NIR light irradiation. This model was evaluated in terms of its controllable 4D transformation and the feasibility of modulating neural stem cell behaviors with photothermal stimulation. Moreover, a NIR light‐sensitive cardiac construct featuring a highly aligned microstructure and tunable curvature was fabricated through 4D printing [230]. Given the variation in curvature across the surface of the heart, these 4D‐printed cardiac constructs can dynamically adapt their shape to replicate the curved topology of myocardial tissue, facilitating seamless integration.

#### 5.2.4 Magnetic Field‐Responsive Biomaterials

Magnetic field‐responsive smart bioinks can interact with external magnetic fields in a remote and highly controllable manner. In contrast to physical actuators or chemical triggers, magnetic fields eliminate the need for direct physical contact or invasive wiring, offering distinct advantages for biomedical applications such as targeted drug delivery, tissue regeneration, and minimally invasive implantation techniques [231]. The precision and sustained effectiveness of magnetic stimuli—whether applied as continuous static fields or dynamic alternating fields—enable long‐term control and activation of 3D printed constructs. This capability to dynamically regulate cellular environments and scaffold architecture on demand positions magnetic field‐responsive biomaterials at the forefront of programmable and intelligent tissue engineering systems.

The fundamental responsiveness of magnetic field‐responsive smart bioinks is typically achieved by incorporating magnetic nanoparticles (MNPs) into the bioink's polymer matrix [232]. Among the most commonly used MNPs are iron oxide (Fe_3_O_4_) and cobalt ferrite (CoFe_2_O_4_), which are known for their strong magnetic properties and tunable surface chemistry. To enhance their dispersion, colloidal stability, and biocompatibility, these MNPs are often functionalized with surfactants, usually polymers such as polyethylene glycol (PEG), or specific proteins. Once integrated into hydrogels or other scaffold materials, MNPs impart magneto‐responsiveness through two primary mechanisms. Under static magnetic fields, they enable manipulation, alignment, and targeted localization of printed constructs. These fields can direct magnetized structures to specific sites within the body, enabling non‐invasive spatial control. In contrast, alternating magnetic fields (AMFs) induce localized heating through magnetic hysteresis and/or generate physical oscillations of MNPs. This energy conversion can activate mechano‐transduction pathways in embedded cells or trigger the response of thermally sensitive biomaterials, thereby offering dual functionality for dynamic tissue modulation.

**FIGURE 10:**
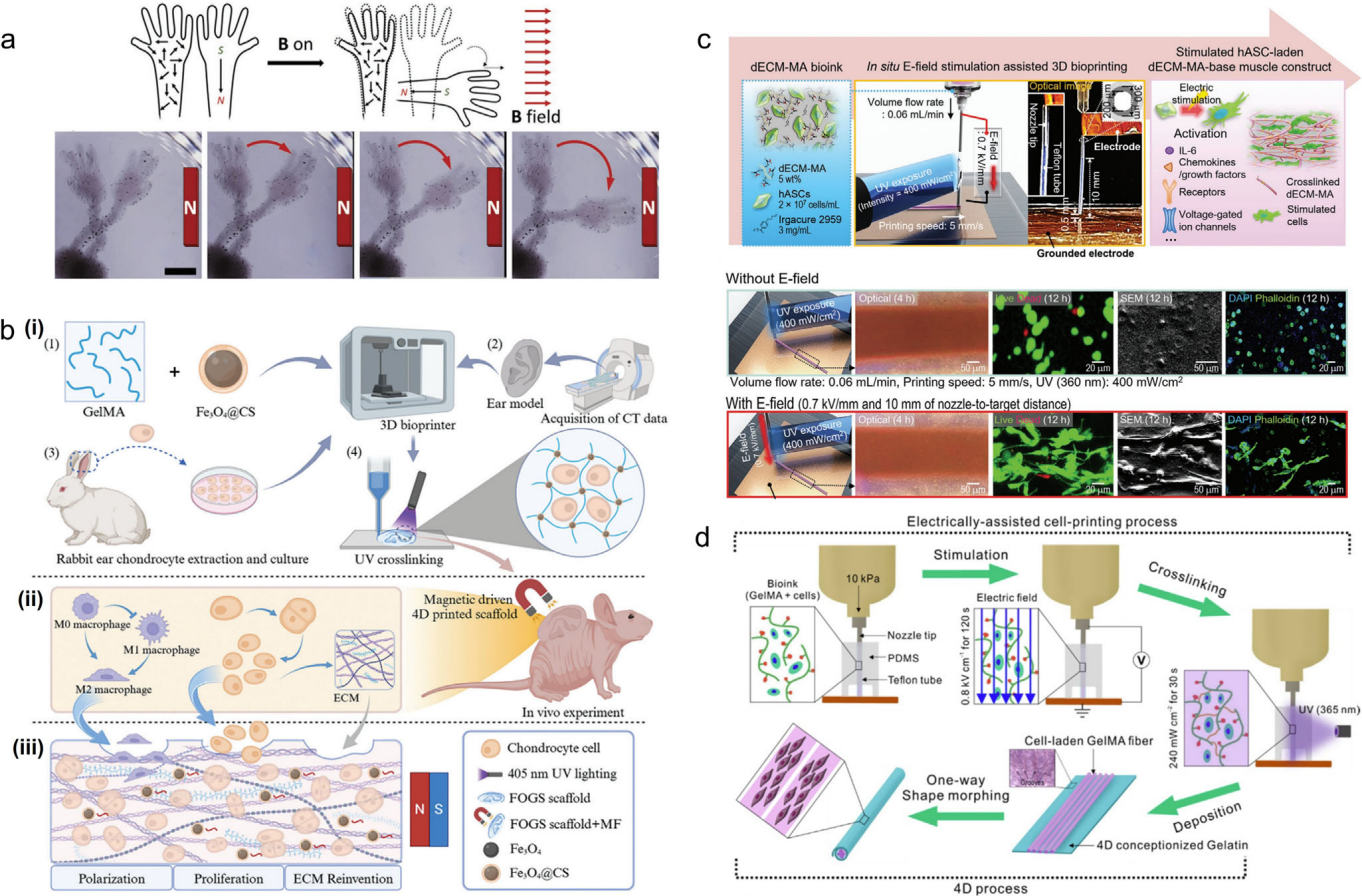
Magnetic and electric field‐responsive biomaterials for 4D printing/bioprinting. (a) Unpaired movement of patterned 4D‐printed shape‐morphing PNIPAM/Fe_3_O_4_ hydrogels. Reproduced with permission [234]. Copyright 2022, Wiley. (b) Schematic illustration of (i) 3D‐printed magnetic hydrogel scaffolds, (ii) subcutaneous implantation into mice and the application of magnetic field to achieve 4D magnetism‐driven regulation of the scaffold, (iii) in vivo scaffold development for enhancing cartilage regeneration. Reproduced with permission [235]. Copyright 2025, Wiley. (c) Schematic illustration of bioprinting process supplemented with in situ electrical stimulation for hASCs‐laden structures and behavior of hASCs with/without electrical stimulation. Reproduced with permission [237]. Copyright 2021, Wiley. (d) 4D bioprinting process for the fabrication of muscle fiber‐like structures. Reproduced with permission [238]. Copyright 2021, Ivyspring International Publisher.

For 4D printing and bioprinting, the spatial control of MNP distribution within the printed construct is of critical importance. By generating MNP gradients or patterns of concentrated MNPs, researchers can design materials that, when subjected to a magnetic field, are capable of bending, folding, twisting, or rotating in a programmable manner. This precise regulation of deformation pathways facilitates the development of sophisticated, shape‐morphing implants that can adapt to physiological conditions or meet specific surgical requirements. For example, Wang and colleagues developed a shape‐transformative structure by precisely controlling the deposition pattern of PNIPAM/Fe_3_O_4_ inks on an elastomer substrate [233]. They observed that the printed scaffolds underwent a rapid volume collapse under an alternating magnetic field due to the magnetothermal effect. This phenomenon arose because of the mismatch in the response rates between the magnetic hydrogels and the elastomer, thereby enabling shape transformation of the composite structure. Additionally, Kuhnt and coworkers employed anisotropic Fe_3_O_4_ MNPs to precisely control the movement of 4D printed structures, enabling complex motions of attraction or repulsion depending on the direction of the applied magnetic field [234]. As shown in Figure 10a, a pair of “hands” was 4D printed using PEGDA/Fe_3_O_4_ inks, where the left hand contained randomly oriented MNPs and the right hand featured MNPs aligned along its longitudinal axis. Upon application of a magnetic field, the randomly oriented MNPs in the left hand resulted in no significant movement, whereas the aligned MNPs in the right hand moved toward the field, thereby generating a waving motion.

In 4D bioprinting, smart bioinks that combine MNPs with supportive hydrogel matrices such as Alg, gelatin, or fibrin are widely utilized to balance magnetic functionality and cellular viability. These hydrogels offer a biocompatible and hydrated environment conducive to cell survival and differentiation, while the embedded MNPs enable remote controllability. Zhang and coworkers developed a magnetoresponsive hydrogel scaffold by incorporating chitosan‐coated, magnetic ferroferric oxide nanoparticles (Fe_3_O_4_@CS MNPs) into chondrocyte‐laden GelMA bioinks (Figure 10b) [235]. The 4D bioprinted magnetic hydrogel scaffolds demonstrated significantly enhanced cartilage tissue regeneration both in vitro and in vivo under the application of an external magnetic field.

#### 5.2.5 Electric Field‐Responsive Biomaterials

Electric field‐responsive biomaterials are increasingly being recognized in the development of smart and adaptive systems for tissue engineering and 4D bioprinting [236]. Their capacity to transduce electrical signals into mechanical or chemical responses renders them particularly suitable for applications where electrical activity is integral to physiological function. This characteristic is especially pertinent in the engineering of electroactive tissues, such as nerves, skeletal muscle, and cardiac muscle. These biomaterials can deform, swell, or facilitate ion transport in response to applied electrical stimuli, thereby providing not only structural support but also enabling functional interactions with biological systems. Electric field‐responsive biomaterials that interface with electrically excitable cells offer dynamic platforms for real‐time communication with host tissues to promote their regeneration and functional restoration.

Biomaterials with electric responsiveness are typically ionic hydrogels or conductive polymers. Ionic hydrogels, such as Alg, chitosan, and PAA, are responsive to electric fields due to the mobility of ions embedded within their polymer networks. When subjected to electrical stimulation, these ions redistribute, creating electrochemical and osmotic gradients that induce anisotropic swelling, bending, or shape transformation. These characteristics render ionic hydrogels highly suitable for applications in soft actuators and ion‐mediated signaling within tissue constructs.

In contrast, conductive polymers such as polypyrrole (PPy), polyaniline (PANI), and PEDOT:PSS demonstrate a distinct mechanism of responsiveness. These materials undergo reversible redox reactions in response to electrical stimulation, leading to changes in volume and conductivity. These changes can be utilized to simultaneously realize mechanical actuation and signal transmission, enabling engineered scaffolds capable of delivering both mechanical and electrical stimuli to cells. When incorporated into bioinks or composite matrices, conductive polymers facilitate the recreation of native electrochemical microenvironment essential for cellular functions, especially in electrically excitable tissues.

For example, a bioengineered skeletal muscle construct composed of human adipose‐derived stem cells (hASCs) and methacrylated decellularized ECM was fabricated using in situ electrical stimulation‐enhanced 3D bioprinting to successfully induce aligned muscle tissue structures [237]. In this study, electrical stimulation was applied concurrently with the bioprinting process to achieve functional hASC differentiation, promoting cytoskeletal alignment and myogenesis by modulating the membrane potential and intracellular mechanical stresses in hASCs. The activation of voltage‐gated ion channels, which regulate multiple signaling pathways, resulted in enhanced cell viability and fully aligned cytoskeletal organization in the printed constructs (Figure 10c). Such cytoskeletal alignment was not observed in conventionally bioprinted (without electrical stimulation) cell‐laden structures. In another study, Yang and colleagues developed a skeletal muscle model using a GelMA‐based bioink capable of promoting cell alignment during electric field‐assisted 4D bioprinting [238]. Electrical stimulation applied to the GelMA fibers induced directional cell alignment. This electric field‐induced alignment significantly enhanced myotube formation, resulting in better‐organized and more mature myotubes than the conventional electric field‐free bioprinting process. Furthermore, the researchers fabricated a fiber bundle that mimics the natural architecture of skeletal muscle, which was capable of undergoing shape morphing to form a tubular structure (Figure 10d).

### 5.3 Chemical Stimuli‐Responsive Biomaterials

#### 5.3.1 pH‐Responsive Biomaterials

**FIGURE 11:**
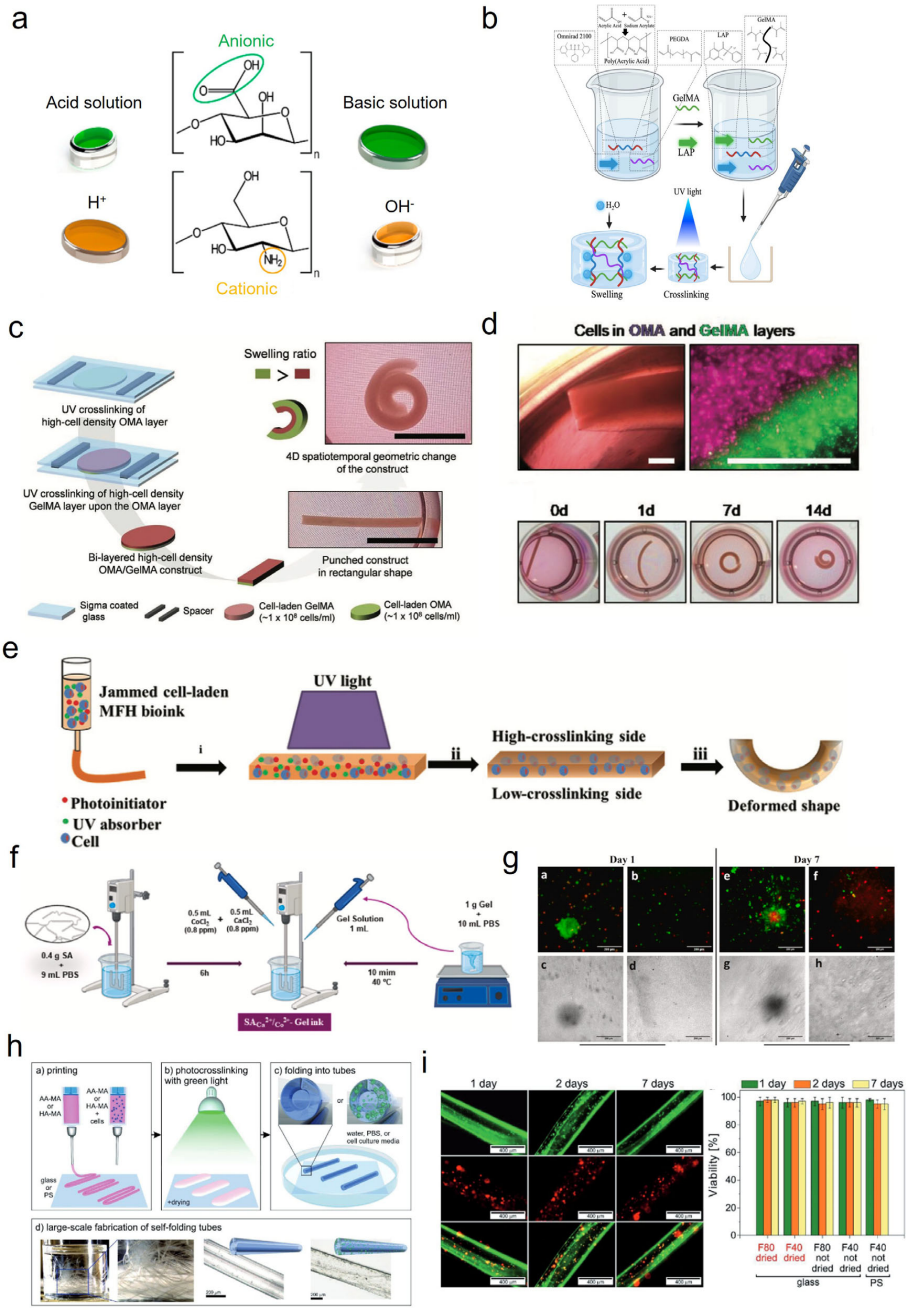
Chemical‐responsive biomaterials for 4D bioprinting. (a) Schematic showing the swelling/deswelling behavior of pH‐responsive hydrogels in acidic and basic solutions, depending on their ionic nature, anionic or cationic. Reproduced with permission [239]. Copyright 2020, MDPI. (b) Schematic of SwelMA [combining gelatin methacryloyl (GelMA) and sodium polyacrylate (SPA)] synthesis. Reproduced with permission [242]. Copyright 2025, Royal Society of Chemistry. (c) Schematic diagram for fabricating 4D high cell density model construct based on different swelling ratios between oxidized and methacrylated alginate (OMA) and GelMA. (d) Photographic images over 14 days of 4D high cell‐density constructs composed of 0.2 mm cell‐laden GelMA and 0.4 mm cell‐laden OMA layers, with NIH3T3 cells in both layers. Reproduced with permission [243]. Copyright 2021, Wiley. (e) Scheme of the photoinitiator‐ and UV absorber‐incorporated 4D micro‐flake hydrogel (MFH) bioprinting: i) printing the jammed cell‐laden MFH bioinks into a bioconstruct; ii) UV exposure to generate a crosslinking gradient within the 3D printed bioconstruct; iii) culturing the bioconstruct in media to drive shape morphing. Reproduced with permission [244]. Copyright 2022, Wiley. (f) Illustrative diagram detailing the preparation of sample SA_Ca_^2+^/_Co_^2+^‐gelatin [pre‐crosslinking sodium alginate (SA) and gelatin inks with divalent cations Ca^2+^ and Co^2+^]. (g) Cell viability in the SA_Ca_^2+^/_Co_^2+^‐gelatin bioink on day 1 and day 7 after 3D printing. Reproduced with permission [247]. Copyright 2024, Elsevier. (h) Schematic of the 4D biofabrication of self‐folding, hydrogel‐based (cell‐laden) tubes. (i) Representative fluorescence microscopy images of the cell‐laden methacrylated alginate (AA‐MA) tubes after 1, 2, and 7 days of culture (left) and quantitative analysis of the viability of cells inside the self‐folded tubes for all the tested samples (right). Green fluorescence images depict live cells within the self‐folded tubes, red fluorescence images show the total cell population (live + dead), and merged green/red channels distinguish live cells (orange) from dead cells (red). Reproduced with permission [22]. Copyright 2017, Wiley.

pH fluctuations serve as indicators of physiological processes. pH‐responsive hydrogels exhibit volumetric changes—swelling and deswelling—in response to alterations in pH levels, driven by the ionization of functional groups within the hydrogel matrix (Figure 11a). The generation of ions modifies the osmotic pressure, thereby regulating the hydrogel's dynamic swelling behavior [239]. These materials contain chemical groups (e.g., carboxyl, pyridine, sulfonic, phosphate, and tertiary amines) that release or accept protons when pH changes [240]. The ionization state of these functional groups—charged or neutral—shifts as external pH traverses a critical threshold, inducing expansion or contraction of the material [241]. PAA and poly(methacrylic acid) (PMAA) represent foundational pH‐responsive polymers whose conformational transitions are governed by carboxyl group ionization. These transitions modulate chain hydration states, enabling programmable drug release through pH‐dependent diffusional gating. However, exposure to extreme pH conditions can adversely affect cell viability. Therefore, stimuli‐responsive materials that function within the narrow pH range characteristic of physiological environments are generally preferred to ensure optimal cell survival and function. Additionally, during the phase transition of pH‐responsive materials, the potential release of embedded compounds into the cell culture medium must be carefully considered; such released substances should not markedly influence cell behavior or functionality.

Gonzalez‐Fernandez’ group engineered SwellMA, a high‐swelling composite hydrogel integrating GelMA with sodium polyacrylate (SPA) (Figure 11b). This design synergistically merges GelMA's high cytocompatibility and cell‐instructive properties with SPA's swelling capacity, enabling pH‐triggerable 4D architectural reconfiguration for dynamic biomimetic structuring [242]. The Alsberg group pioneered 4D‐bioprinting platforms using oxidized methacrylated Alg/gelatin methacryloyl (OMA/GelMA) composites densely laden with cells (Figure 11c,d). These systems enable geometric complexity beyond conventional biofabrication limits, achieving programmable architecture reconfiguration through spatiotemporally controlled crosslinking gradients [243]. Furthermore, the cell‐encapsulated OMA microgranular inks developed by the Alsberg group allow light‐directed structural bending and exhibit pH‐modulated shape‐morphing behavior (Figure 11e), where protonation‐induced carboxyl exposure triggers differential network contraction/expansion for biomimetic dynamic patterning [244].

Although pH‐responsive biomaterials have exhibited programmable shape morphing in vitro, their translation into cell‐laden 4D bioprinting systems has been limited by insufficient validation within physiologically relevant environments. In particular, the demonstration of cytocompatible, pH‐triggered structural transformations in complex biological milieus is scarce. To address this gap, future research should focus on the development of multilayered composite bioinks—such as Alg‐GelMA hybrids that exploit the protonation of Alg's carboxyl groups under acidic conditions (pH < 4.5)—to enable spatially resolved morphogenesis while maintaining high cell viability. These systems must be rigorously evaluated in environments with physiological pH gradients to ensure translational relevance. Additionally, dynamic covalent networks based on hydrazone chemistry, although not yet fully harnessed for programmable 4D shape evolution, offer a compelling design framework due to their rapid and reversible bond exchange kinetics. Integrating such adaptable crosslinking motifs into cell‐laden matrices, particularly in conjunction with extrusion‐based bioprinting, may unlock spatiotemporally encoded deformation capabilities, enabling the fabrication of next‐generation constructs with latent, stimulus‐activatable structural reconfiguration.

#### 5.3.2 Ion‐Responsive Biomaterials

Ionic concentration‐responsive biomaterials represent a class of smart hydrogel systems capable of undergoing changes in volume, shape, stiffness, or mechanical properties in response to variations in the concentrations of specific ions, such as Ca^2^^+^, Mg^2^^+^, and Co^2^^+^. For successful application in 4D bioprinting, such materials must exhibit not only dynamic deformability but also high biocompatibility and sustained structural fidelity [245]. There are generally three types of ion‐responsive mechanisms: differential ion‐induced crosslinking, ion concentration‐mediated modulation of pre‐crosslinking density, and reversible ion exchange‐driven transformations. Harnessing these mechanisms in a controlled and spatially defined manner holds considerable potential for engineering reconfigurable, stimuli‐adaptive constructs that mimic the complexity of native tissue microenvironments [246].

Marcos and colleagues employed a composite system consisting of Ca^2^^+^ and Co^2^^+^ ions to pre‐crosslink Alg, which was supplemented with gelatin to impart a thermosensitive response (Figure 11f,g). This approach resulted in a bioink with tunable rheological properties. After printing, the shape of the constructs could be precisely modulated by manipulating the temperature and ionic conditions, enabling the fabrication of 4D‐printed structures with biological functionality [247]. Ionov's research team developed a double‐layer Alg/HAp bioink containing cells, wherein Ca^2^^+^ ions were employed to induce interlayer swelling differences (Figure 11h,i). This mechanism facilitated the self‐folding of the construct into a tubular structure with a diameter comparable to that of microvessels. Importantly, cell viability was preserved for at least seven days, demonstrating the potential of this approach for creating functional, biomimetic vascular structures [22]. Ozbolat and colleagues recently introduced an ionically responsive microgel platform (ionically crosslinked microgel, IP‐XMG) that enables cell‐friendly, spatiotemporally controlled 4D bioprinting. This system leverages reversible ion‐induced particle crosslinking to achieve exceptional moldability during extrusion‐based printing while maintaining structural responsiveness to ionic triggers [248].

Alg‐based bioinks represent a foundational material in 4D bioprinting owing to their excellent cytocompatibility and rapid ionic crosslinking induced by physiologically benign Ca^2^^+^, which supports high viability of encapsulated cells. However, efforts to enhance the stiffness of printed constructs using alternative divalent cations, such as Co^2^^+^, Ba^2^^+^, Mn^2+^, and Zn^2^^+^, are hindered by dose‐dependent cytotoxicity that compromises cell viability and functions. To address this inherent trade‐off between mechanical robustness and biocompatibility, innovative strategies have emerged, most notably the incorporation of pre‐formed cellular spheroids as discrete biofabrication units. Unlike conventional single‐cell suspensions, spheroids preserve cell–cell interactions and reduce direct cellular exposure to cytotoxic agents, thereby enhancing cellular viability and functional integrity during post‐printing shape transformations [247].

Despite these advancements, current ion‐responsive biomaterial‐based bioinks remain limited by their reliance on a narrow spectrum of crosslinking ions, leaving critical gaps in our understanding of how underexplored, biologically relevant cations, such as Zn^2^^+^, Mg^2^^+^, and Fe^3^^+^, modulate the structural and dynamic performance of bioinks. Bridging this knowledge gap is essential for developing next‐generation 4D constructs with programmable shape‐morphing capabilities. Future progress depends on the rational design of multi‐ionic bioinks that leverage orthogonal cationic interactions and spatially encoded ion gradients to enable reversible, stimuli‐responsive behaviors. Two primary objectives must be prioritized: (1) elucidating the synergistic or antagonistic roles of specific ion combinations in governing hydrogel deformation through systematic experimentation, and (2) developing multi‐modal responsive systems that integrate temperature, pH, enzymatic activity, and ionic cues to more faithfully recapitulate the complex biochemical milieu of native tissues.

### 5.4 Biological Stimuli‐Responsive Biomaterials

While physicochemical stimuli‐responsive bioinks have dominated previous 4D bioprinting studies, their clinical translation is often impeded by the need for external actuation devices, potential tissue damage, and limited penetration depth. In contrast, endogenous stimuli‐responsive bioinks, which are materials capable of sensing and responding to localized in vivo cues such as inflammatory cytokines, disease‐specific enzymes, pH shifts, and oxidative stress, offer a pathway to intelligent, autonomous therapeutic systems. Yet, studies on these bioinks specifically tailored for 4D bioprinting are still scarce, indicating a crucial gap that the field must address urgently.

#### 5.4.1 Glucose‐Responsive Biomaterials

**FIGURE 12:**
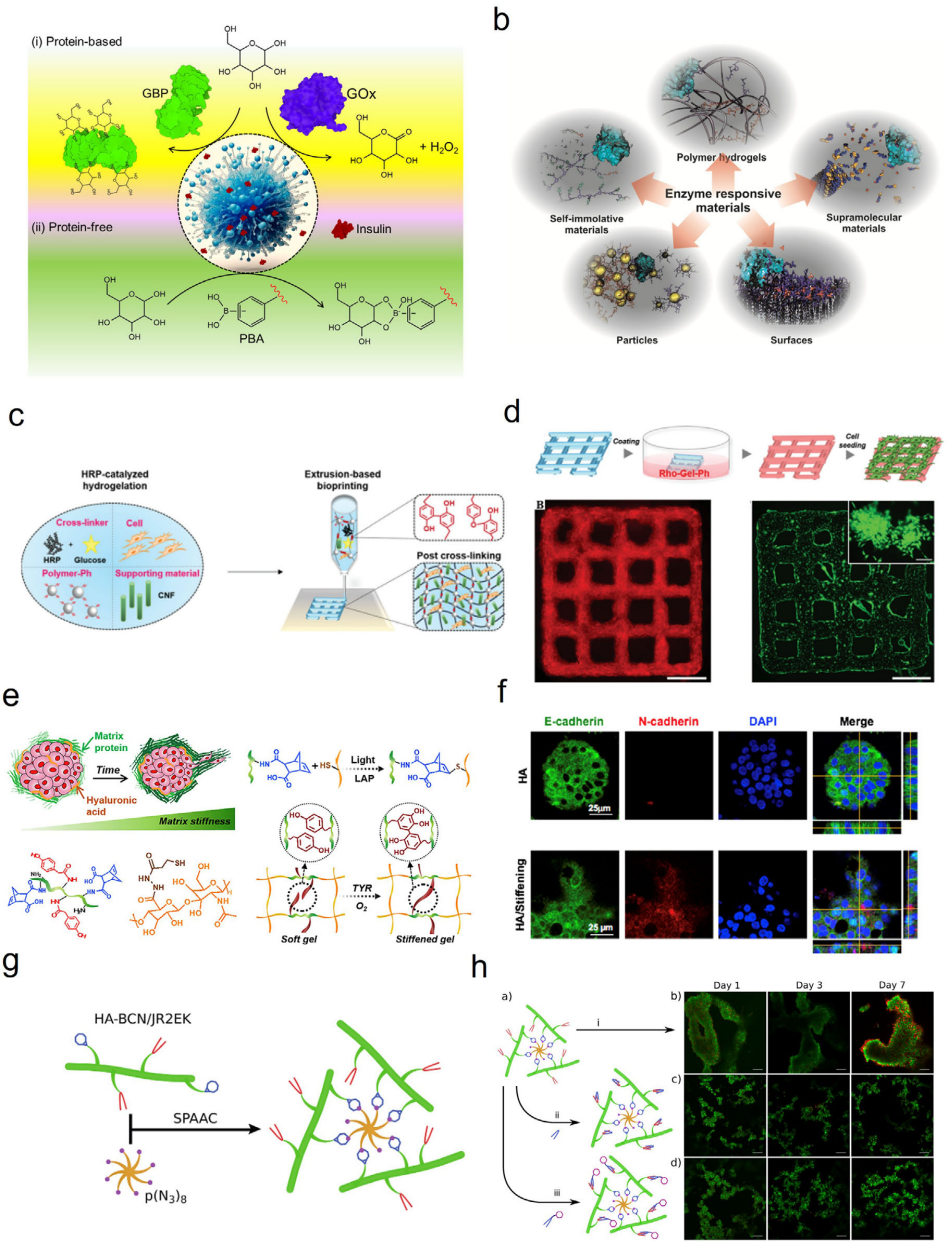
Biological stimuli‐responsive biomaterials for 4D bioprinting. (a) Schematic diagram of glucose‐responsive material fabricated from (i) protein‐based and (ii) protein‐free strategies. Reproduced with permission [258]. Copyright 2025, American Chemical Society. (b) The classification of enzyme‐responsive materials based on their structural design elements. Reproduced with permission [252]. Copyright 2013, Royal Society of Chemistry. (c) Schematic of extrusion‐based bioprinting through glucose‐mediated enzymatic hydrogelation. (d) Workflow of modifying hydrogel surface after printing and following crosslinking (top). Fluorescence images of printed lattice‐shaped hydrogel after coating (bottom left) and 10T1/2 cells cultured on the hydrogel for 2 days (bottom right). Reproduced with permission [206]. Copyright 2020, Accscience Publishing. (e) Enzyme‐triggered on‐demand stiffening of biomimetic hydrogels for in vitro cancer cell research. (f) Evaluation of selected epithelial and mesenchymal markers in COLO‐357 cells grown in hyaluronic acid‐containing soft gel (HA), or in HA‐containing TYR‐stiffened gel (HA/stiffening). Reproduced with permission [207]. Copyright 2018, Elsevier. (g) Schematic representation of bioorthogonal crosslinked hydrogels. (h) Supramolecular tuning of cell‐hydrogel interactions using SH‐SY5Y neuroblastoma cells. Reproduced with permission [257]. Copyright 2020, IOP Publishing.

Originally developed for diabetes management and smart insulin delivery, glucose‐responsive biomaterials are now being increasingly repurposed for applications in advanced tissue engineering and 4D bioprinting [249]. These materials enable the fabrication of dynamically reconfigurable scaffolds, stimulus‐responsive architectures, and adaptive therapeutic platforms capable of sensing and responding to physiological glucose fluctuations (Figure 12a). Their integration into biofabrication strategies holds significant potential for creating living systems with enhanced functionality, responsiveness, and therapeutic precision [250, 251]. For instance, Sakai and colleagues developed an extrusion‐based bioprinting strategy in which the printed bioink was stabilized via horseradish peroxidase (HRP)‐mediated enzymatic crosslinking (Figure 12c,d). In this system, hydrogen peroxide (H_2_O_2_), required for the HRP‐catalyzed reaction, is generated in situ through the reaction of HRP with glucose. The bioink formulation comprises living cells, HRP, glucose, phenol‐conjugated Alg (Alg‐Ph), and cellulose nanofibers, enabling the fabrication of 3D hydrogel constructs with enhanced structural fidelity and biocompatibility [206].

Although glucose‐responsive materials have demonstrated considerable utility in drug delivery applications, the integration of these materials into cell‐laden, dynamically morphing constructs for 4D bioprinting is still in its infancy. Current research efforts predominantly focus on two foundational aspects: (1) the development of printable bioinks incorporating glucose‐sensing elements, particularly hydrogels functionalized with phenylboronic acid that exploit reversible diol interactions, and (2) the rigorous validation of cytocompatibility and functional performance within cellularized systems. These efforts represent critical prerequisites for the realization of genuinely bioresponsive 4D constructs, wherein embedded cells not only endure but actively participate in glucose‐triggered shape and function transformations. Advancing this field will require the strategic integration of multimodal responsive chemistries with cutting‐edge bioprinting platforms to engineer next‐generation bioinks capable of (1) programmable shape morphing in response to physiologically relevant glucose gradients, (2) reversible actuation cycles that recapitulate native biological rhythms, and (3) maintenance of cell‐instructive microenvironments that preserve long‐term phenotypic stability. Achieving this triad—intelligent deformation, dynamic reciprocity, and cytocompatibility—will be essential for constructing adaptive, living systems capable of autonomous feedback within metabolically active niches.

#### 5.4.2 Enzyme‐Responsive Biomaterials

Enzyme‐responsive hydrogels represent a class of dynamically programmable biomaterials that undergo precise structural reconfiguration or mechanical modulation through specific enzyme‐substrate interactions. These systems exploit enzymatic catalysis, such as protease‐mediated peptide cleavage or transglutaminase‐driven crosslinking, to emulate physiological remodeling processes observed in native ECM (Figure 12b) [252]. Their biorthogonal responsiveness has enabled transformative applications spanning spatiotemporally controlled drug release, hierarchical tissue scaffold fabrication, and 4D bioprinting of adaptive architectures [253]. Particularly in 4D bioprinting applications, these materials enable three critical functionalities: (1) simulate the dynamic remodeling of tissue microenvironments; (2) utilize enzyme‐triggered deformation to achieve self‐organizing behavior in tissue construction; and (3) endow printed structures with sequential functional activation capabilities [254].

The design of enzyme‐responsive hydrogels leverages a diverse repertoire of biocatalysts that enable precise spatiotemporal control over material properties. Matrix metalloproteinases (MMPs)—naturally secreted by cells for ECM remodeling, often accompanying tissue degeneration or tumor invasion/metastasis—provide precise cleavage of engineered peptide crosslinkers, allowing cell‐directed hydrogel degradation. Tyrosinase drives phenolic coupling reactions, facilitating bioinspired polymer crosslinking through tyrosine‐rich domains. Glucose oxidase (GOx) converts ambient glucose into hydrogen peroxide, serving as both a dynamic crosslinking initiator and a stimulus for secondary oxidative reactions. Proteases and peptidases offer sequence‐specific hydrolysis of designed peptide linkers, creating temporal control over network integrity. Multiple peroxidases are produced by neutrophils and immune cells as a result of inflammation and during wound healing. Such enzymatic diversity enables orthogonal control mechanisms where multiple enzymes can operate concurrently without interference, a critical feature for engineering complex, multifunctional biomaterial systems.

However, because eliciting a response without exogenous enzymes requires overexpression of the relevant enzyme, enzyme‐responsive mechanisms are typically slow. An enzyme that has been determined to trigger the swelling and degradation of specific hydrogels is matrix metalloproteinase (MMP). To replicate the dynamic remodeling behavior of native ECM, Kim and coworkers synthesized MMP‐sensitive, HA‐based hydrogels, which not only exhibited tunable enzymatic degradation profiles but also supported robust MSC attachment and proliferation [255]. In parallel, Sortase A, a bacterial transpeptidase, has emerged as a promising enzymatic crosslinker for hydrogel‐based bioinks. Its rapid gelation kinetics and low immunogenicity make it particularly attractive for 4D bioprinting applications requiring cytocompatible and temporally controlled network formation [256]. Lin and colleagues engineered an enzymatic crosslinking paradigm where tyrosinase‐mediated conjugation of bisphenol motifs in hydroxypropyl acrylamide (HPA) generates programmable matrix stiffening, recapitulating ECM mechanotransduction dynamics (Figure 12e,f). This enzyme‐triggered rheological evolution provides a biomimetic platform for investigating stiffness‐dependent cancer cell behaviors in vitro, while its spatiotemporally controllable gelation kinetics offers distinct advantages for constructing 4D‐bioprinted architectures with graded mechanical cues [207]. As exemplified by Daniel and coauthors, precisely folded peptide architectures can be programmed to modulate both crosslinking density and biological functionality within bioprinted constructs (Figure 12g,h). Such systems exemplify the emerging paradigm of enzyme‐triggered 4D bioinks, where spatiotemporal control over material properties allows for the creation of biologically active scaffolds that progressively evolve to mimic native tissue development and remodeling processes [257].

While enzyme‐responsive hydrogels offer controllable degradation and high biocompatibility, which are favorable qualities for tissue regeneration scaffolds, their use as bioinks in 4D bioprinting remains largely confined to mechanistic studies, and a well‐defined cell‐laden, shape‐morphing system has yet to be established. Nevertheless, enzyme‐responsive hydrogels hold great potential in simulating ECM remodeling and controlling intelligent structures, important directions for future intelligent bioprinting research.

## 6 Applications of 4D bioprinting

### 6.1 Tissue Engineering

#### 6.1.1 Skin

The programmed dynamic properties of 4D bioprinted constructs offer significant advantages for addressing the complex and evolving needs of wound healing. These constructs can conform to irregular wound geometries, promote tissue regeneration, and help combat infections in skin repair applications. Unlike 3D printed/bioprinted skin scaffolds, which are static and limited in functionality, 4D bioprinted constructs offer personalized solutions that enhance healing outcomes through smart hydrogels capable of changing their properties by responding to external stimuli. For example, a pH‐responsive hydrogel blend comprising gallic acid (GA)‐functionalized HA and HAMA was developed for extrusion‐based bioprinting of wound dressing [259]. GA is a polyphenol compound with three phenol units known as catechol moieties and is recognized for its tissue adhesive properties and antioxidant activity. The bioprinted HAGA/HAMA constructs remained stable at varying pH values during the skin healing process and exhibited excellent tissue adhesion and antioxidant activity, showing high potential for defected skin repairing.

**FIGURE 13:**
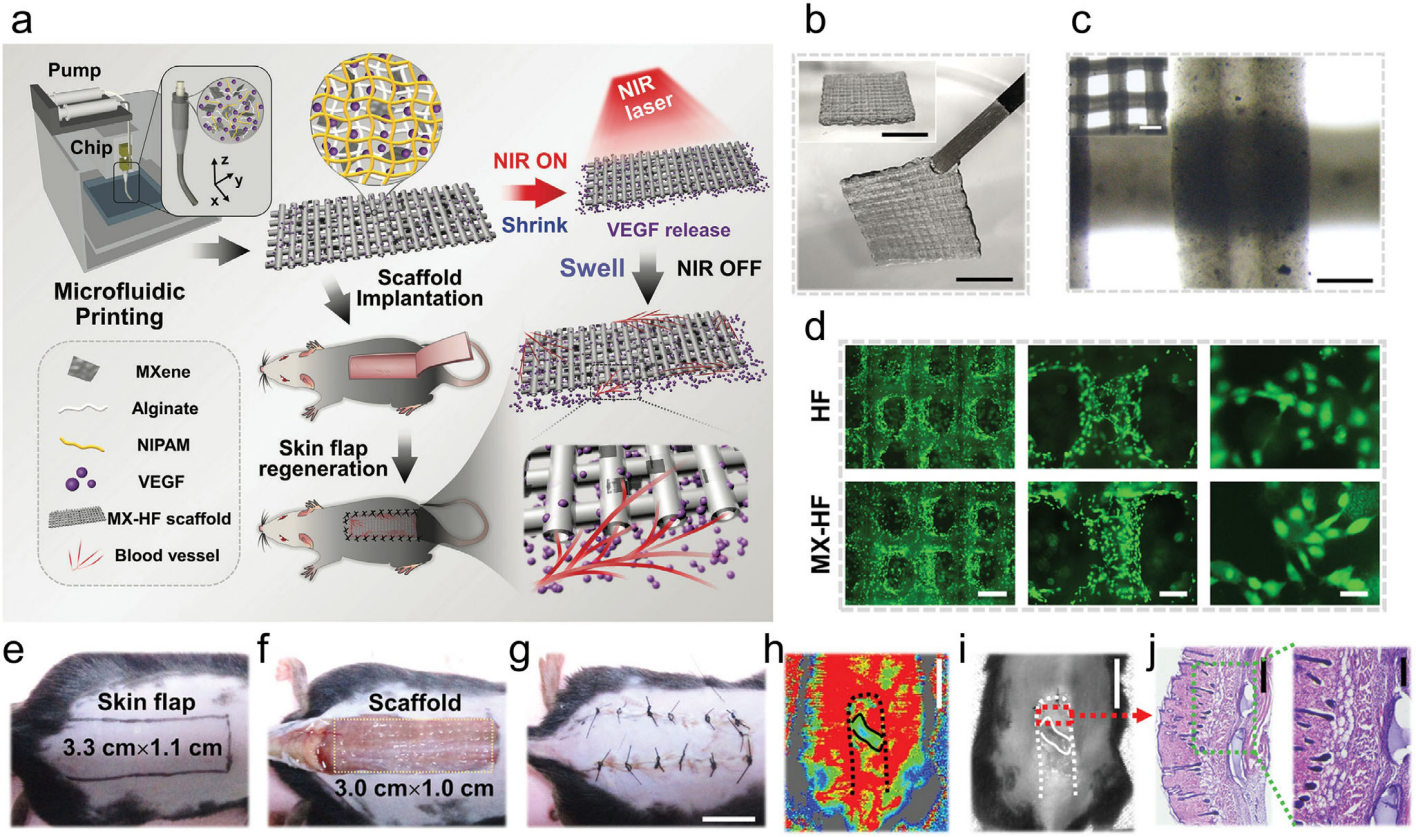
4D printed skin scaffolds for skin flap regeneration. (a) Schematic illustration of dynamically responsive scaffolds fabricated via a coaxial capillary microfluidic 3D printing strategy. (b) Photographs showing the printed MXene‐incorporated hollow fibrous (MX‐HF) scaffolds (scale bar: 1 mm). (c) Optical micrographs showing the MX‐HF with straight channels (scale bar: 200 µm and inset scale bar: 500 µm). (d) Live/dead images showing the viability of HUVECs adhering to the surface of HF (without MXene) and MX‐HF (with MXene) scaffolds 24 h after printing (Scale bars indicate 1 mm, 300 µm, and 50 µm from left to right). (e–g) Photographs showing implantation of the hollow scaffolds into a rat with a skin defect (scale bar: 1 mm). (h–j) Histological analysis of the necrotic junction of the skin flaps 9 days after skin recovery: (h) real‐time blood flow image captured with laser speckle contrast imaging (scale bar: 1 cm), (i) corresponding photos of skin flaps (scale bar: 500 µm); (j) H&E staining showing necrosis and survival junction area of the skin flap (scale bars indicate 500 and 200 µm for the left and right image, respectively). Reproduced with permission [260]. Copyright 2022, Wiley.

By incorporating bioactive agents, such as antibiotics, growth factors, or anti‐inflammatory compounds, 4D bioprinted constructs can respond to wound‐specific stimuli and deliver therapeutics on demand. For instance, NIPAAM is a commonly used temperature‐responsive hydrogel that undergoes shrinking/swelling in response to changes in surrounding temperature. Using its temperature‐sensitive reversible phase behavior, Wang and colleagues 4D printed dynamic scaffolds using a smart bioink containing Alg, NIPAAM, MXene, and VEGF for skin flap regeneration (Figure 13) [260]. Upon NIR irradiation, heat was generated due to the photothermal effect of MXene, triggering NIPAAm shrinking and enabling controlled VEGF release to the defective skin. This contributed to improved angiogenesis and skin regeneration. In another study, a smart ink for DLP 4D printing was obtained by combining NIPAAM with curcumin‐loaded Pluronic F127 micelles (as an antibacterial agent), and poly(ethylene glycol) diacrylate‐dopamine (as a crosslinker and to ensure tissue adhesion) [261]. The thermo‐responsive property of NIPAAM facilitated wound contraction triggered by the physiological temperature (37°C), promoting healing in full‐thickness skin defects in diabetic rat models. These studies highlight the ability of 4D bioprinting to create dynamic living constructs that address the multifaceted challenges of skin repair, including vascularization, infection prevention, and tissue integration.

#### 6.1.2 Bone

**FIGURE 14:**
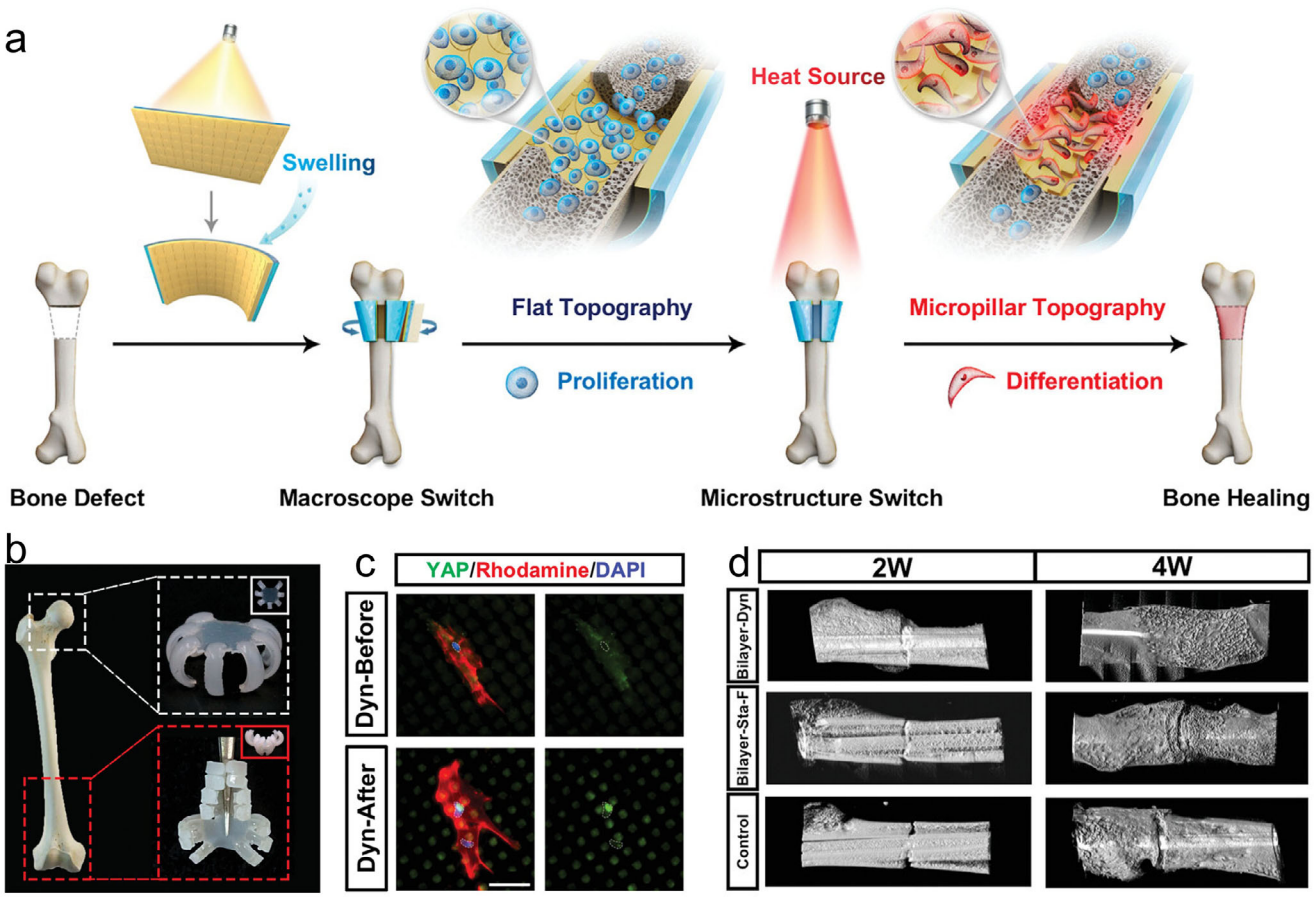
4D‐printed dual‐responsive scaffolds for bone tissue engineering. (a) Schematic demonstration of 4D printed bilayer membrane for bone repair with water‐responsive self‐folding behavior for fitting defect geometry and heat‐responsive microstructural transformation for inducing cell differentiation. (b) Photographs showing 4D‐printed bilayer membranes tmorphing to conform to the bone model. (c) Fluorescence images of cells stained with Rhodamine (red), YAP (green), and DAPI (blue) on scaffolds with/without heat‐triggered microstructural transformation (Scale bar: 50 µm). (d) Micro‐CT images showing fracture repair outcomes at week 2 and 4 for the control group (without any scaffolds), static membrane, and 4D printed dynamic bilayer membrane. Reproduced with permission [263]. Copyright 2021, Wiley.

4D bioprinting as an advanced biofabrication technique has facilitated the creation of advanced bone tissue engineering scaffolds with programmed shape or functional transformations in response to environmental cues, thereby enhancing integration with native tissue and bone regeneration [262]. The programmable 3D deformation of these scaffolds addresses the demand for customized bone defect repair, while functional maturation processes enhance the osteogenic differentiation of stem cells. By integrating stimuli‐responsive biomaterials with advanced 4D bioprinting techniques, dynamic scaffolds have been developed to meet the multifaceted demands of bone repair. For instance, Wang and coworkers developed photothermal‐responsive shape memory bone tissue engineering scaffolds via cryogenic extrusion 4D printing of a nanocomposite combining black phosphorus nanosheets, osteogenic peptide (OP), β‐TCP, and PLATMC [227]. Upon NIR irradiation, the black phosphorus nanosheets heated the scaffolds to 45 °C due to the photothermal effect, triggering shape reconfiguration to precisely fit irregular bone defects. After implantation, the sustained release of OP enhanced osteogenesis at bone defect sites, accelerating bone regeneration of rat cranial bone defects. In another study, You and colleagues manufactured a dual‐responsive membrane combining an SMP layer and a hydrogel layer via DLP 4D printing (Figure 14) [263]. The SMP was synthesized via thermal radical polymerization of two poly(ε‐caprolactone)‐diacrylates (PCLDA) with molecular weights of 2,000 and 10,000, respectively. The hydrogel layer was obtained by photocrosslinking a mixture of 2‐hydroxy ethyl acrylate, PCLDA, and 3‐sulfopropyl methacrylate potassium salt. The SMP layer was featured with temperature‐responsive surface microstructures that toggled between proliferation and differentiation phases to promote bone formation. The hydrogel enabled the membrane to adapt its 3D geometry upon swelling to match specific bone defect shapes after deployment. In vivo studies demonstrated that this 4D shape‐shifting membrane achieved over 30% greater new bone formation compared to a static microstructure membrane, highlighting the potential of dynamic scaffolds to enhance bone regeneration outcomes. However, these shape‐memory scaffolds were not fabricated using cell‐laden bioinks during the printing process; instead, cells were loaded post‐fabrication through a cell‐seeding approach. In contrast, Alsberg's group developed a smart bioink comprising oxidized and methacrylated Alg (OMA), a UV absorber, and hMSCs, which was biofabricated into strips with a crosslinking gradient across their thickness direction [220]. These cell‐laden strips self‐folded into curved structures upon immersion in osteogenic medium and maintained their curvature during four weeks of in vitro culture. During the incubation period, the cells maintained high viability and differentiated toward bone tissues, as indicated by increased ALP expression and calcium deposition.

#### 6.1.3 Articular Cartilage

Smart bioinks that combine biocompatibility with stimuli‐responsive properties enable 4D bioprinting of dynamic scaffolds that mimic the curvature of native articular cartilage, promoting chondrogenesis and seamless integration with surrounding tissue. For example, Alsberg's group demonstrated this approach by fabricating cartilage constructs using smart jammed bioinks composed of hMSCs, OMA microgels, and a photoabsorber in lithography‐based 4D bioprinting [204, 244]. The photo absorber resulted in a crosslinking gradient across the thickness direction during photocrosslinking, enabling self‐folding of the scaffolds to form curved “C” like structures. During in vitro culture in the chondrogenic medium, the hMSCs in the curved hydrogels retained high viability and differentiated toward chondrocytes, as indicated by the expression of glycosaminoglycan and collagen type II. Similarly, in another study, Wu and coauthors developed a jammed smart bioink composed of Alg methacrylate (AlgMA), TGF‐β1 loaded GelMA microspheres, a photoabsorber, and hMSCs [264]. The smart bioink was extrusion‐4D bioprinted into strips, which generated a gradient network during photocrosslinking and transformed into curved shapes during in vitro incubation. Under the stimulation of sustained TGF‐β1 release, MSCs laden in the hydrogels underwent chondrogenic differentiation with the production of glycosaminoglycan, type II collagen, and aggrecan.

**FIGURE 15:**
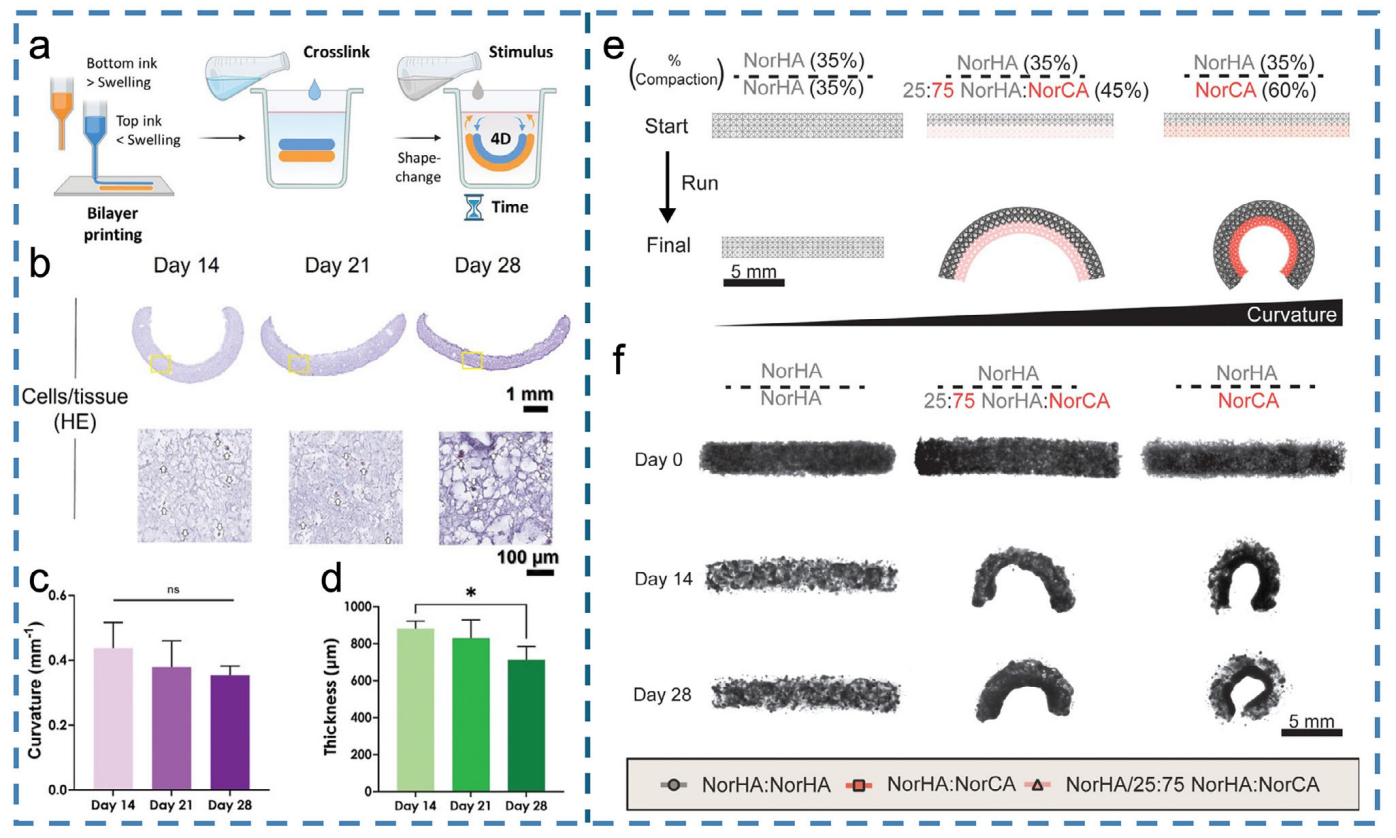
4D biofabrication of cell‐laden self‐folding strips for cartilage tissue engineering. (a‐c) Extrusion 4D bioprinting of self‐folding bilayer constructs using two types of bioinks, tyramine‐functionalized HA (HAT, high swelling) and Alg/HAT (low swelling), with different swelling degrees: (a) Schematic illustration of 4D bioprinting of the bilayer constructs. (b) HE staining images of the self‐folding hMSC‐laden strips after 14, 21, and 28 days of culture in chondrogenic differentiation medium, and corresponding (c) quantified curvature and (d) thickness. Reproduced with permission [121]. Copyright 2023, Wiley. (e, f) 4D biofabrication of cell‐laden granular composites with self‐folding ability. (e) Schematic illustration of the self‐folding bilayer strips fabricated using different types of granular composites and (f) corresponding curvature during in vitro culture at different time points. Reproduced with permission [265]. Copyright 2025, American Association for the Advancement of Science.

Besides 4D bioprinting of single smart bioinks, multi‐bioink 4D bioprinting has been performed to create programmable shape‐morphing scaffolds for articular cartilage tissue engineering. In the study by Park's group, a silk‐based smart bioink was developed to fabricate bilayer self‐folding cartilage structures using DLP 4D bioprinting [181]. In the bioprinting process, the first layer was bioprinted as a solid membrane with a silk bioink containing MSCs, while the second layer was printed into a specific pattern with a silk bioink containing chondrocytes. The two layers were cured over different exposure times, resulting in the formation of a gradient network that enabled self‐folding behavior of the construct upon immersion in water. The researchers employed FEA for predictive design of specific shape transformation. The well‐designed 4D bioprinted structures were then implanted into a damaged trachea of a rabbit for 8 weeks. The implants integrated with the host trachea naturally, and both epithelium and cartilage were formed at the predicted sites, indicating the potential of 4D bioprinted tissue‐mimetic structures in regenerative medicine. In another study, Díaz‐Payno and colleagues performed 4D bioprinting of bilayer cartilage structures using two ink formulations: tyramine‐functionalized hyaluronan (HAT, high‐swelling) and Alg with HAT (AHAT, low‐swelling), as shown in Figure 15a‐d [121]. The differential swelling of the two layers led to the self‐bending of the scaffolds upon immersion in water. Subsequently, hMSCs were incorporated into the AHAT, followed by biofabrication of self‐curved, cell‐laden strips. After 28 days of in vitro culture in chondrogenic medium, the scaffolds retained clear curvatures with visible cartilage matrix production in histological analysis. In another study by Burdick's group, two HA‐based granular composite bioinks with differing hydrolytic stabilities were developed to create curved structures for articular cartilage tissue engineering (Figure 15e,f) [265]. By combining MSC spheroids with stable (NorHA) and degradable (CA or NorCA) microgels in a bilayer configuration, the composites achieved programmable shape transformations, with the degradable layer driving mechanical compaction and curvature and the stable layer providing structural support. These dynamic scaffolds exhibited enhanced deposition of ECM, particularly sulfated glycosaminoglycans, and maintained stable curved morphologies over 28 days of culture in chondrogenic medium, demonstrating potential for engineering dynamic articular cartilage constructs.

#### 6.1.4 Vasculature

4D bioprinting represents a powerful manufacturing platform for fabricating vascular structures that are critical for nutrient delivery and waste removal in engineered tissues. Traditional 3D bioprinting often struggles to replicate the intricate, hierarchical vasculature with small‐diameter features like capillaries, while 4D bioprinting enables more precise control over vascular structures by using shape‐morphing hydrogels. As presented by Kirillova and coworkers, extrusion 4D bioprinted AlgMA and HAMA strips self‐folded into tubular structures with internal diameters as small as 20 µm, comparable to natural capillaries [22], demonstrating the capability of 4D bioprinting in fabricating high‐resolution vascular networks.

**FIGURE 16:**
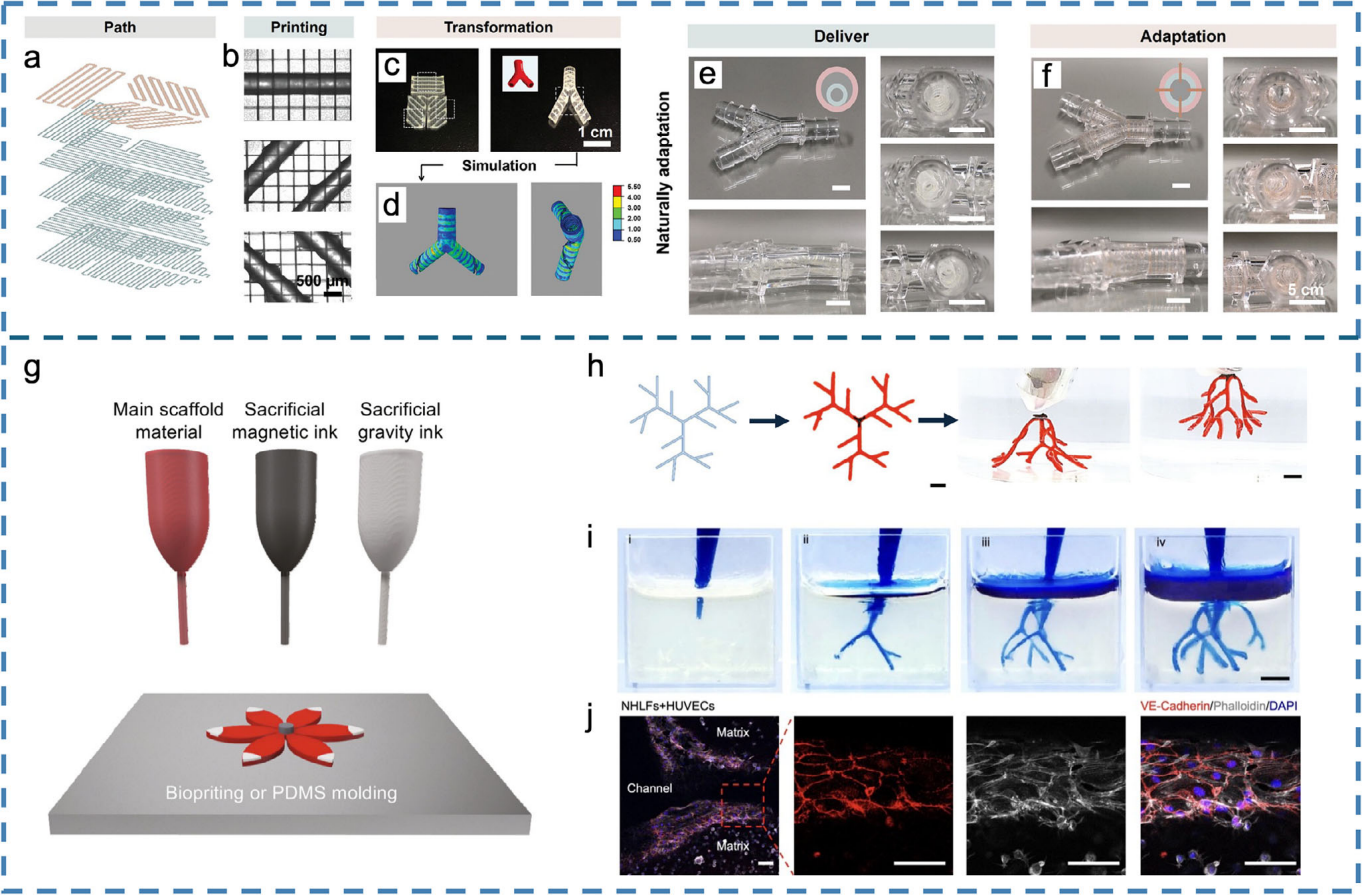
4D printing of vascular constructs. (a–f) 4D printing self‐tubing bifurcated constructs. (a) Design of the multi‐layered Janus scaffolds. (b) Optical microscope images showing the printed 2D patterns. (c) 2D‐to‐3D transformation of the 2D patterns to become a bifurcated vessel and corresponding (d) FEA‐simulated results. (e) Photographs showing the transformation of the 4D‐printed scaffolds to a branching vascular model, and (f) subsequent shape change of the scaffold to fit the geometry of the vascular model. Reproduced with permission [177]. Copyright 2024, Wiley. (g‐j) Magnetic 4D printing of complex vascular networks. (g) Schematic illustration of the 4D printing process using three types of inks. (h) 4D printing process showing the path design, 4D printed pattern, and following magnetic field‐driven shape morphing to form a vascular network within a matrix (Scale bar: 1 cm). (i) Photograph showing the injection of a blue dye into the hollow vascular network within a fibrin matrix (Scale bar: 5 mm). (j) Immunostaining for VE‐cadherin (red), F‐actin (gray), and DAPI (blue) in the fibrin scaffold with HUVECs lining the channel wall and human lung fibroblasts (NHLFs) within the fibrin matrix. (Scale bar: 100 µm). Reproduced with permission [266]. Copyright 2024, American Association for the Advancement of Science.

Recent developments further highlight the potential of 4D bioprinting in vascular reconstruction. Plant leaves typically exhibit an asymmetric bilayer structure comprising an adaxial mesophyll layer and an abaxial vein layer. Inspired by this, Wang and colleagues introduce a Janus bilayer scaffold with a passive PCL fibrous layer, resembling the “mesophyll” layer of the leaves, and an active GelMA/Alg hydrogel layer, resembling the leaves’ “vein” layer [177]. The passive layer of submicron PCL mesh was fabricated via melt electrowriting, followed by deposition of a hydrogel layer onto the PCL layer via direct ink writing. The curvature and curved direction of the 4D Janus scaffold were controlled by adjusting the matrix stiffness of the passive PCL layer and the pattern of the active hydrogel layer, achieving shape morphing into complex tubular shapes with different diameters and directions, as seen in bifurcated vessels (Figure 16a–d). This Janus vascular scaffold could undergo multi‐step transformations triggered by shrinking/swelling of the hydrogel layers and physiological conditions, adapting to intricate vascular geometries when deployed in vivo (Figure 16e,f) [177]. In another study by Stevens’ group, a magnetically driven approach was employed to form freestanding 3D vascular channels within hydrogels, replicating branching topologies and supporting endothelialization [266]. This strategy employed extrusion‐based bioprinting to fabricate 2D patterns from three bioinks: 1) a gelatin‐based ink serving as the primary scaffold, 2) a magnetic ink containing iron oxide particles and gelatin, and 3) a gravity ink incorporating calcium carbonate and gelatin (Figure 16g). Post‐printing, these 2D patterns were submerged in a matrix bath and transformed into 3D branching vascular structures under an applied magnetic field (Figure 16h). The resulting 3D configurations were stabilized by the interplay of buoyancy, gravity, and magnetic forces until the matrix was fully crosslinked. Subsequently, the gelatin‐based vasculature was removed, yielding hollow vascular networks within the matrix (Figure 16i). The hollow vascular networks supported HUVEC growth and functionality during in vitro culture (Figure 16j) [266]. These innovative studies highlight the capability of 4D bioprinting to integrate advanced materials and external stimuli, such as magnetic fields and hydration changes, to produce perfusable, viable cell‐seeded vascular networks. Advances in 4D bioprinting pave the way for shaping soft hydrogel biomaterials into complex 3D vascular morphologies to empower a broad range of vasculature tissue engineering applications.

#### 6.1.5 Other Tissues

4D bioprinting is highly promising for engineering other dynamic tissues such as skeletal muscle, uterine, and cardiac tissues, which require dynamic behaviors to mimic their physiological geometries and functions. The regeneration of skeletal muscle tissue, which is essential for locomotion and constitutes 40%–45% of the body mass, requires scaffolds that promote uniaxial cell alignment, particularly for conditions like volumetric muscle loss. 4D bioprinting can address this need by creating shape‐morphing scaffolds that guide myoblast alignment and support myogenesis. For instance, Ionov's group employed 3D extrusion printing to prepare an AlgMA hydrogel membrane, on which PCL fiber patterns were deposited by melt electrowriting to create bilayer scaffolds that could transform to scroll‐like structures upon swelling. The scroll direction was dependent on the PCL fiber direction, and the formed scroll‐like scaffolds promoted uniaxial cell orientation along the PCL fibers, enhancing skeletal muscle regeneration [267]. Similarly, another study by the same group utilized the same fabrication technique to fabricate bilayer scaffolds comprising a layer of solid HAMA hydrogel and a layer of patterned PCL‐PU fibers. These scaffolds transformed from 2D planar structures to tubular ones, achieving high cell viability and up to 57.5% myoblast alignment, both of which were critical for muscle tissue functionality [268].

**FIGURE 17:**
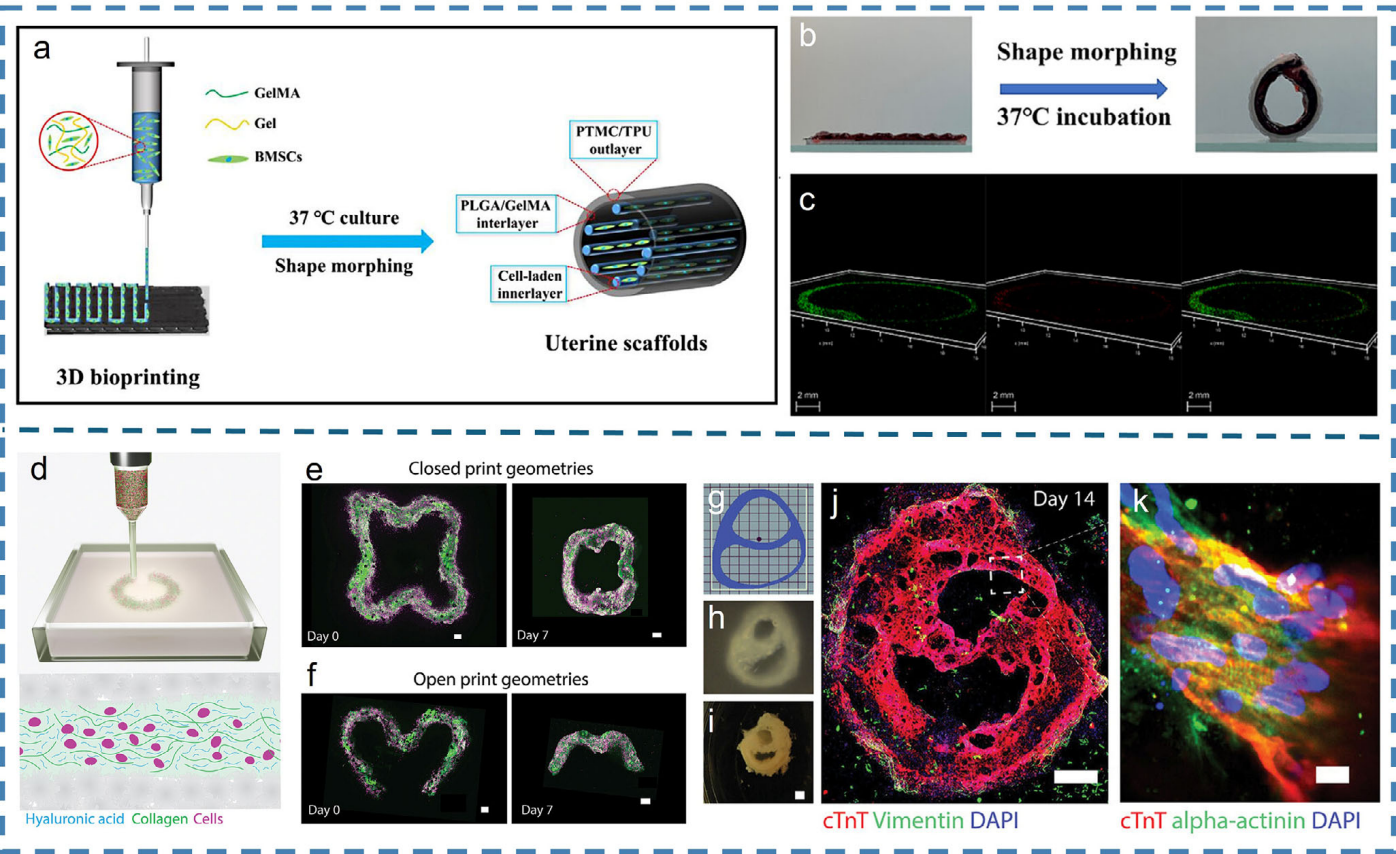
4D bioprinting of other tissue types. (a‐c) Hybrid 4D biofabrication of curved structures for uterine tissue engineering. (a) Schematic illustration of extruding cell‐laden bioinks onto the shape‐memory layer to fabricate trilayer curved scaffolds. (b) Photographs presenting the self‐folding behavior of the trilayered scaffolds at 37 °C. (c) Fluorescent images showing the 3D distribution of BMSCs in folded trilayer scaffolds. Reproduced with permission [40]. Copyright 2024, Wiley. (d‐k) Embedded 4D bioprinting of cardiac tissues using collagen‐HA bioinks. (d) Schematic illustration of the embedded 4D bioprinting technique. (e, f) Confocal images of bioprinted constructs with (e) closed and (f) open geometries undergoing 4D shape‐morphing within the granular support hydrogel over 7 days of culture (Scale bar: 500 µm). (g‐k) A 4D bioprinted double ventricle heart tissue construct: (g) CAD design, (h, i) brightfield images showing the bioprinted construct at (h) day 0 and (i) day 14 (Scale bar: 1 mm), (j, k) Confocal images of (j) cTnT/Vimentin organization and (k) cTnT/sarcomeric alpha‐actinin organization within the bioprinted tissues at day 14 (Scale bar: 1000 µm for figure j and 10 µm for figure k). Reproduced with permission [36]. Copyright 2024, Wiley.

Uterine tissue engineering aims to address infertility caused by damage to the myometrium and endometrium, requiring scaffolds that replicate the uterus’ hierarchical curved structure and dynamic functionality [269]. 4D bioprinting has enabled the development of trilayer scaffolds that mimic the perimetrium, myometrium, and endometrium, with shape‐morphing capabilities to match the uterus's natural curvature. Wang's group utilized a combination of solvent casting, layer‐by‐layer assembly, and extrusion bioprinting to create trilayered scaffolds with a stretchable PLATMC/PLGA shape‐memory base layer (by solvent casting), an estradiol (E2)‐loaded polydopamine (PDA)/HA drug‐delivery middle layer (via layer‐by‐layer assembly), and a BMSC‐laden functional gelatin/GelMA hydrogel inner layer (by extrusion bioprinting). These scaffolds morphed from planar to tubular shapes at 37°C, with controlled E2 release enhancing uterine regeneration [41]. In another study, a trilayered uterine scaffold was fabricated with similar temperature‐driven bending properties by combining 4D printing, electrospinning, and extrusion bioprinting (Figure 17a–c). Specifically, a highly stretchable PLATMC/PLGA shape memory base layer was first 4D printed using high‐temperature extrusion printing, followed by electrospinning of E2‐loaded PDA/GelMA/PLGA fibers onto the base layer, and finally bioprinting of a BMSC‐laden gelatin/GelMA hydrogel [40]. These trilayered scaffolds achieved physiological temperature‐driven curvature, hierarchical structure, high stretchability, and excellent biocompatibility, offering a promising approach to uterine repair.

Cardiac tissue engineering has been extensively explored to create functional heart tissues that replicate the structural and contractile properties of native myocardium, with applications in regenerative medicine and drug screening. 4D bioprinting has been applied to develop dynamic cardiac scaffolds that undergo cell‐driven shape transformations to enhance cardiac tissue maturation. For instance, Daly's group performed embedded bioprinting with collagen‐HA bioinks in yield‐stress granular support hydrogels composed of agarose microgels to create heart tissues that morphed in response to fibroblast‐generated forces (Figure 17d–k) [36]. Co‐culture of iPSC‐derived cardiomyocytes (iPSC‐CMs) with human cardiac fibroblasts (HCFs) improved contractile properties and sarcomere alignment, contributing to the structural organization and maturation of iPSC‐CMs in the shape‐morphing heart tissues. This approach mimicked the developmental processes of native heart tissue, offering potential for engineering organ rudiments with improved functionality.

### 6.2 Disease Modeling and Drug Screening

**FIGURE 18:**
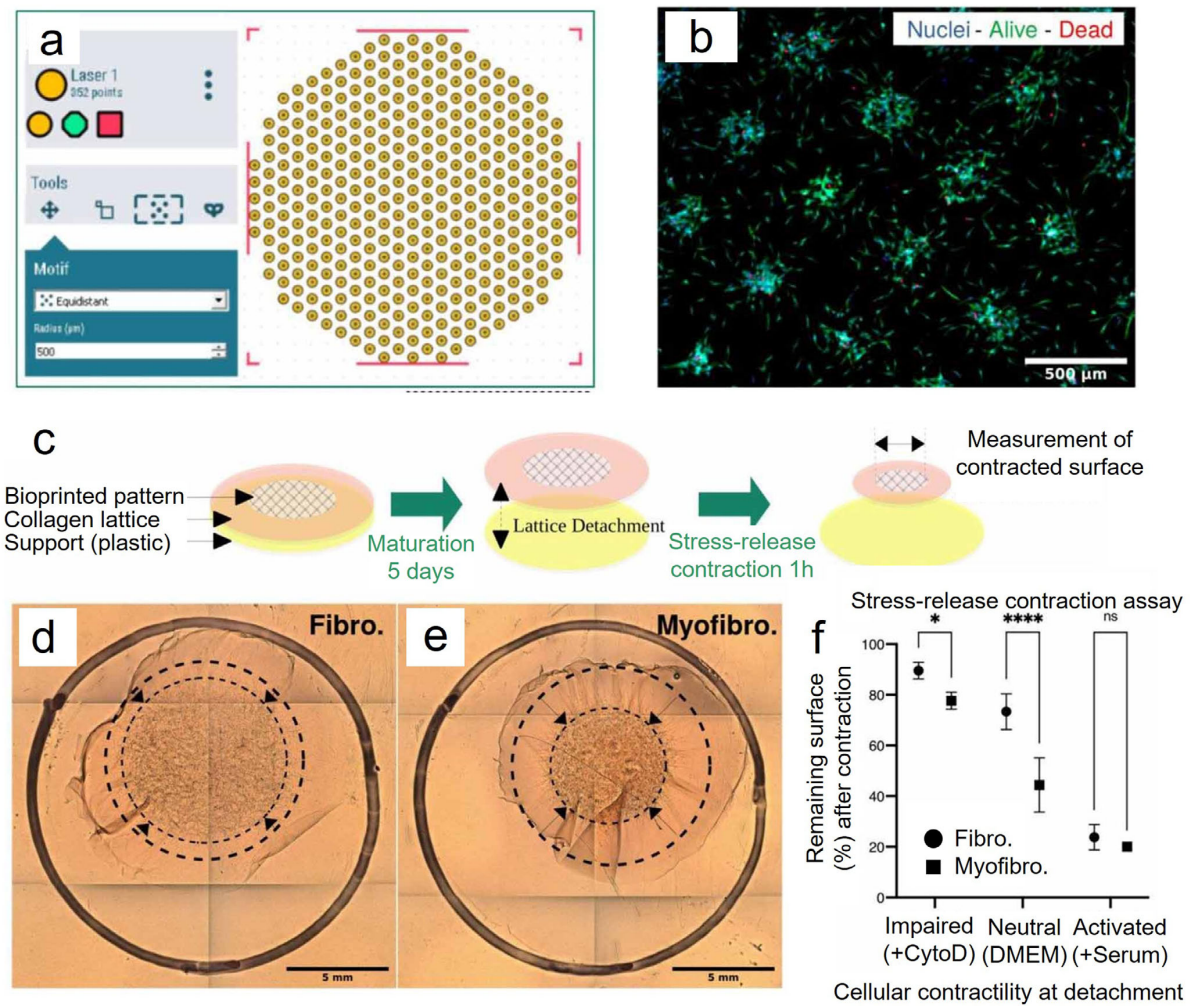
4D laser‐assisted bioprinting of skin models for investigating dynamic interactions between (myo)fibroblasts and the ECM during skin repair. (a) Cell pattern design. (b) Live/Dead image showing viability of bioprinted fibroblasts on collagen after 2 days of culture. (c) Schematic illustration of the collagen matrix contraction process due to the bioprinted (myo)fibroblasts under the cell‐induced shear stress. (d,e) Optical microscopy images showing collagen contracted by the (d) fibroblasts and (e) myofibroblasts (outer and inner dotted lines indicated the change in collagen lattice size during the contraction process). (f) Contracted surface area as a percentage of original bioprinted surface before detachment under different culture conditions: with Cytochalasin D to inhibit cellular contractility, in DMEM, or in DMEM with 10% serum to promote cellular contractility. Reproduced with permission [270]. Copyright 2022, IOP Publishing.

4D bioprinted constructs can replicate the dynamic nature of pathological processes, such as tumor growth, tissue healing, or inflammatory responses, providing a physiologically relevant platform to study disease mechanisms and evaluate therapeutic interventions. For instance, Douillet and coworkers utilized laser‐assisted bioprinting (LAB) to create dynamic, evolving constructs that mimicked human skin (Figure 18) [270]. These models featured fibroblasts and myofibroblasts precisely patterned on collagen matrices, enabling controlled matrix remodeling over time. Fibroblasts promoted fine, anisotropic organization, while myofibroblasts formed contractile clusters, simulating scar formation. Such 4D bioprinted skin models provided insights into dynamic matrix remodeling and dermal organization during wound healing.

Furthermore, 4D bioprinted models offer a physiologically relevant environment to assess drug efficacy and toxicity over time, improving predictive accuracy compared to conventional models. For instance, 4D bioprinting was used for glioblastoma research by creating patient‐derived organoid (PDO) models for drug screening [271]. In this work, a shape‐memory polymer composed of bisphenol A ethoxylate dimethacrylate and PEGDA was SLA‐manufactured to obtain 3D cell‐culture arrays that transformed from 3D inserts into histological cassettes over time due to temperature changes. These arrays, housing glioblastoma PDOs, mimic the tumor's structure and behavior more accurately than traditional 2D cultures. The transformation from culture inserts to histological cassettes streamlined handling and analysis, enabling rapid drug response assessments. Such 4D bioprinted PDOs facilitated drug screening by evaluating the efficacy, on‐target activity, and synergy of drugs in an in vivo‐like, patient‐specific environment, offering a faster, more efficient alternative to conventional methods.

### 6.3 Other Applications

**FIGURE 19:**
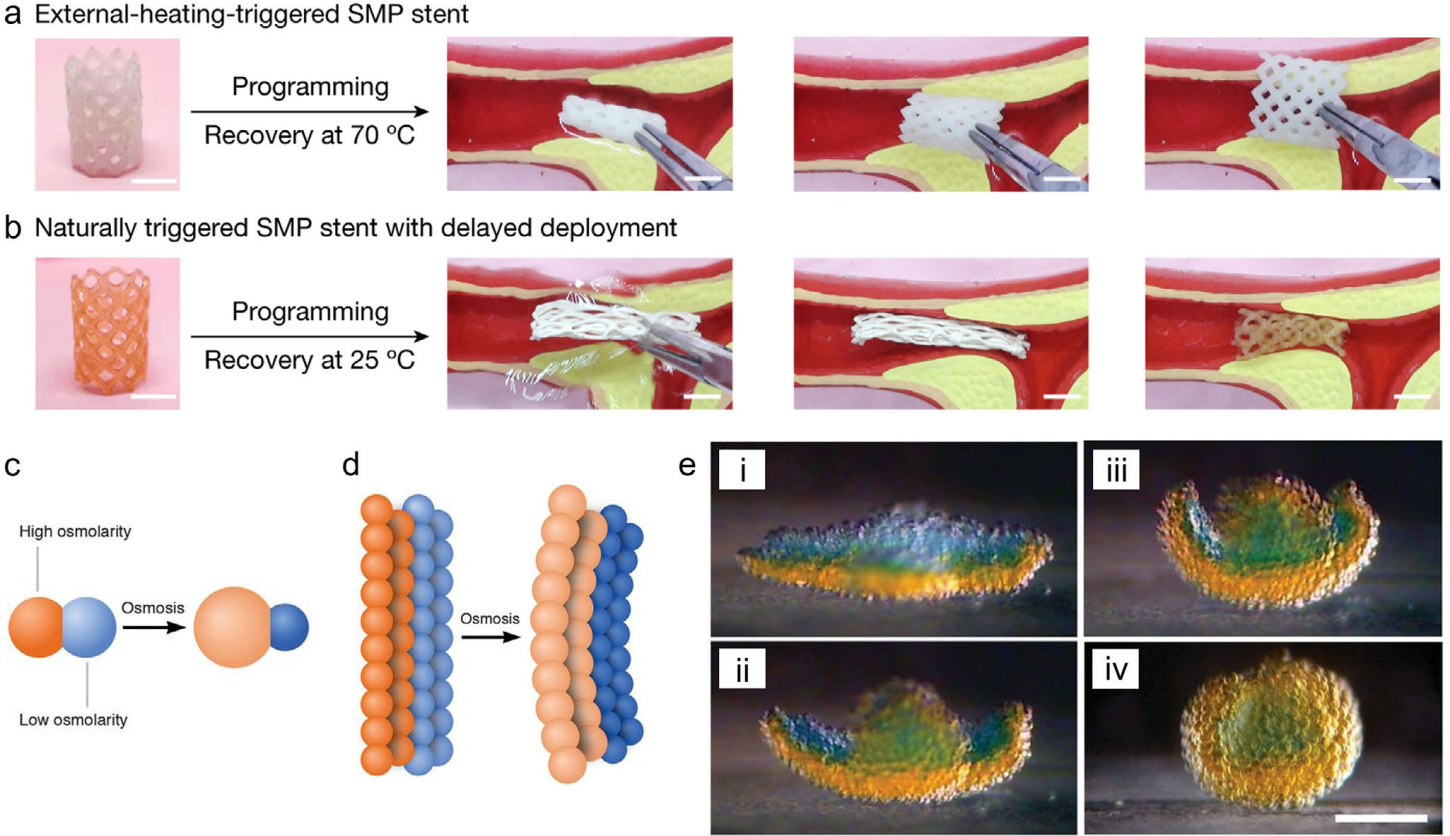
4D printed shape‐morphing constructs for other biomedical applications. (a,b) DLP‐4D printed vascular stents fabricated with (a) conventional SMP (heating in 70°C water) and (b) naturally triggered SMP (triggered in 25°C water), and their shape‐morphing behavior during deployment (Scale bar: 1 cm). Reproduced with permission [272]. Copyright 2023, Springer Nature. (c–e) 4D‐printed self‐folding droplet networks with potential drug delivery applications. (c) Schematic of two droplets with different osmolarities. (d) Schematics of a droplet network that comprises two strips of droplets with different osmolarities, resulting in a curved structure. (e) Photographs showing a flower‐shaped network folding spontaneously into a hollow sphere over a period of 8 h (Scale bar = 200 mm). Reproduced with permission [273]. Copyright 2013, American Association for the Advancement of Science.

4D bioprinting also holds significant promise for other emerging biomedical applications, such as medical devices and drug delivery. In the realm of medical devices, particularly implants, 4D bioprinting offers appealing advantages. For example, shape memory hydrogels, as explored by Ni and coworkers, were DLP‐engineered into smart structures that changed shape at ambient temperatures with a programmable recovery onset delay [272]. This capability is particularly advantageous in the fabrication of devices like stents, which could be delivered in a compact form and then expand to their functional configuration at the target site (Figure 19a,b). The tunable delay in shape recovery enabled precise deployment, addressing challenges in minimally invasive surgeries where premature expansion or external triggers complicate device delivery.

In drug delivery, 4D bioprinting facilitates the development of responsive systems capable of controlled therapeutic release. Villar and colleagues demonstrated the drug‐delivery potential of 4D printed droplet networks, which consisted of lipid‐bound aqueous compartments that could be designed to alter their structure in response to stimuli such as pH and temperature changes (Figure 19c‐e) [273]. Driven by osmotic gradients or protein‐mediated interactions, these networks could fold into hollow spheres to encapsulate drugs and then unfold to release them at specific times or locations. This stimuli‐responsive behavior enhances the precision of drug delivery, offering a significant improvement over conventional systems that lack adaptability to physiological conditions. Apart from the shape reconfiguration for drug delivery, the properties of the smart bioinks can be tuned to achieve smart controlled drug release at the target site. For example, Zu and colleagues presented 4D printing of core‐shell hydrogel capsules for smart, controlled drug release [274]. Using a multi‐material extrusion‐based 4D printing method, the capsules featured a model drug core encapsulated by a temperature‐responsive PNIPAM hydrogel shell. The core‐shell hydrogel capsules exhibited temperature‐dependent drug release because the LCST of PNIPAM enabled it to shrink or swell with temperature changes, facilitating autonomous modulation of drug release. In vitro studies demonstrated that the drug release rates varied with different temperatures, which could be further adjusted by altering the PNIPAM shell thickness. The study highlighted 4D printing's ability to create adaptive, precise drug delivery systems tailored to individual physiological needs.

## 7 Challenges and Future Perspectives

Although 4D bioprinting remains largely in the proof‐of‐concept stage, it holds transformative potential for biomedical applications by enabling the fabrication of programmed, evolving tissues and organs, which is difficult to achieve with conventional 3D printing technologies. Smart bioinks—bioinks composed of cell‐laden materials that exhibit dynamic and stimuli‐responsive properties—are the cornerstone of 4D bioprinting. Currently, increasing efforts are being devoted to the advancement of 4D bioprinting, aiming to overcome the limitations of traditional 3D bioprinting and achieve more dynamic and functional tissue constructs [23, 49, 251, 275]. Nevertheless, considerable limitations and challenges remain.

### 7.1 Biomaterials limitations

Although numerous biomaterials, including metals and alloys, ceramics, polymers, and their hybrids, have been widely used to fabricate tissue constructs via 3D printing technologies, biomaterials suitable for 4D bioprinting remain limited [118, 140]. The main reason is that, compared to traditional inks, smart bioinks used in 4D bioprinting must meet more stringent requirements. They not only need to support cell viability and bioactivity to facilitate tissue formation, but also must exhibit dynamic and stimuli‐responsive properties that enable them to undergo functional transformations or shape morphing in response to external or internal stimuli. Fulfilling these requirements continues to pose significant challenges. Current stimuli‐responsive biomaterials (e.g., SMPs and SMHs) often exhibit poor biocompatibility and are unsuitable for cell encapsulation, as they may lead to cell death and provoke inflammatory responses. This is especially true for SMPs like PLA and PLATMC [39, 213, 276]. Moreover, although SMHs such as Alg, gelatin, and HA exhibit good biocompatibility and can be used to encapsulate cells to form bioinks, they often lack the mechanical strength and stability required for 4D transformations. Consequently, structural collapse may occur during or after bioprinting. Therefore, these natural polymers are commonly chemically modified or blended with additional components to enhance the mechanical properties and stability of the resulting bioinks [117, 205, 277].

Another significant challenge in 4D bioprinting is the inherent trade‐off between the responsiveness and printability of smart bioinks. These properties often conflict, necessitating careful material design and process optimization to ensure that bioinks remain both functionally effective and practically suitable for bioprinting applications. For instance, thermoresponsive bioinks such as PNIPAM undergo rapid sol‐gel transitions near physiological temperatures, which can interfere with the printing process or even cause nozzle clogging [48, 210]. Furthermore, smart bioinks that exhibit high sensitivity to environmental stimuli, such as pH, temperature, moisture, or ionic strength, may experience premature deformation before the intended stimulus is applied, thereby compromising structural integrity.

### 7.2 Biocompatibility and Bioactivity

Smart bioinks with excellent biocompatibility and bioactivity can support cell growth and differentiation while minimizing adverse immune responses. Compared to 3D bioprinting, ensuring both biocompatibility (minimal immune reaction) and bioactivity (ability to support cellular functions) is more challenging in 4D bioprinting due to the incorporation of stimuli‐responsive properties in smart bioinks. Many smart bioinks rely on SMPs or the crosslinking mechanisms of SMHs, which may potentially compromise cell viability or interfere with biological processes. It is well known that certain SMPs, such as PLA and PLATMC, exhibit poor biocompatibility and may trigger inflammatory responses. Moreover, SMHs made of natural polymers typically require crosslinkers to initiate chemical crosslinking, thereby enhancing structural fidelity. For example, certain crosslinkers, such as photoinitiators (e.g., Irgacure 2959) used in light‐responsive smart bioinks, may elicit cytotoxic effects at high concentrations [68, 142]. Furthermore, the dynamic nature of smart bioinks, i.e., the structural or configuration changes after bioprinting, can exert mechanical stress on encapsulated cells, potentially impairing their viability and functionality.

Bioactive smart bioinks support cell viability, proliferation, and differentiation, as well as the realization of specific biological functions within bioprinted constructs. The bioactivity primarily stems from the incorporation of bioactive cues in bioinks, such as growth factors, nanoparticles, and drugs. However, achieving and maintaining effective bioactivity remains challenging due to the difficulty in integrating these bioactive components without compromising the bioink's responsiveness or printability. Additionally, the unpredictable degradation of SMHs can lead to premature release of signaling molecules or loss of structural support before tissue maturation. Furthermore, incorporating multiple bioactive functionalities into a single bioink remains a significant challenge. Many smart bioinks are designed with a focus on a single specific function, which restricts their adaptability and effectiveness in complex biomedical applications.

### 7.3 Control of 4D Transformations

Precise control over 4D transformations of smart bioinks after printing is the cornerstone for 4D bioprinting. The ability to orchestrate predictable, spatially defined, and temporally coordinated shape changes and functional adaptations is critical for generating functional biological constructs that closely mimic natural tissue development and repair processes [24]. Without such control, the dynamic capabilities of 4D bioprinted constructs may become a drawback rather than a benefit, potentially resulting in structural instability, biological incompatibility, and impaired tissue functionality. Although many smart bioinks have been engineered to undergo predefined shape changes or functional transformations, achieving such predictable, spatially controlled, and temporally synchronized transformations remains highly challenging. A major limitation arises from the inherent heterogeneity of biological tissues or organs, where local variations in cell density and type, matrix composition, and pore size and porosity generate unpredictable mechanical stresses that interfere with the intended shape‐morphing behaviors and/or functional transformation. Furthermore, most smart bioinks exhibit isotropic responses to stimuli, which complicates the programming of complex, directional shape changes, such as twisting or folding, without inducing structural deformations or tearing.

Moreover, most current smart bioinks lack temporal precision in shape changes or functional transformations, as these processes often occur at rates that are either too fast or too slow relative to biological timescales. Our recent study demonstrated that the overall shape‐morphing process can be predicted and controlled by adjusting the printed hydrogel layer thickness [205]. However, one limitation is that this humidity‐driven shape‐morphing mechanism may not align with the gradual remodeling dynamics required during tissue development. A general shortcoming of most smart bioinks is the absence of real‐time feedback mechanisms after printing. Without integrated sensors or adaptive control systems, it is extremely difficult to dynamically adjust stimuli to correct deviations from the intended transformation.

Another particularly promising development that directly realizes precise control is reprogrammable 4D bioprinting. In contrast to conventional 4D bioprinting, which usually experiences a single, irreversible shape‐morphing process, reprogrammable approaches allow for repeated, reversible, and on‐demand control over the configuration and functionality of bioprinted constructs [278]. This paradigm shift essentially redefines the concept of “programming” in 4D bioprinting, changing it from a one‐time trajectory to a dynamic, multi‐cycle process in which constructs can be activated, restored to their original state, and reactivated multiple times. Reprogrammable 4D bioprinting represents a fundamental breakthrough that converts the control of 4D transformations from a significant challenge into a powerful capability. By enabling reversible, multi‐cycle, and adaptive shape changes, reprogrammable systems bring 4D bioprinting closer to its ultimate objective: creating truly intelligent, living constructs that can dynamically interact with and respond to their biological environments.

### 7.4 Printing Resolution and Complexity

The printing resolution in 4D bioprinting is much lower than that of conventional 3D printing, mainly due to the incorporation of cells and the requirement of a dynamic nature. In traditional 3D printing, the printing resolution is mainly determined by the rheological properties of inks, printing parameters, and printing technologies. High‐fidelity fabrication techniques such as DLP and 2PP enable the creation of structures with sub‐micron features, whereas FDM is limited to micron‐scale resolution [279, 280, 281]. In 4D bioprinting, the printing resolution of smart bioinks limits our ability to accurately replicate the intricate microstructures of native tissues. As aforementioned, cells in smart bioinks occupy a specific volume within the polymeric matrix, which may influence the rheological properties of the bioinks as well as the efficiency and degree of crosslinking when crosslinking is required. This, in turn, may compromise the printability of bioinks and the structural fidelity of the resulting constructs, ultimately affecting printing resolution [83]. Additionally, unlike conventional inks, smart bioinks must balance printability with stimuli‐responsiveness, often at the expense of high resolution in order to enable stimuli‐triggered shape‐changing properties. Consequently, many stimuli‐responsive hydrogels exhibit suboptimal viscoelastic properties, leading to filament spreading and compromised dimensional accuracy, or structural collapse during deposition.

Furthermore, the printing complexity of smart bioinks in 4D bioprinting is significantly higher compared to conventional 3D printing. While 3D printing is capable of producing intricate, multi‐material constructs with high precision, there are significant challenges in achieving comparable levels of structural complexity without compromising stimuli‐responsive functionality in 4D bioprinting. High‐resolution features, such as microchannels and interconnected micropores, can be preserved in 3D‐printed structures. However, in 4D bioprinting, stimuli‐induced transformations (e.g., swelling, contraction, folding, twisting, etc.) often lead to distortion or collapse of fine architectural details, thereby limiting the achievable structural complexity. When a construct is designed with intricate microarchitecture, its functionality may be compromised after shape morphing or functional transformation due to pore closure or layer misalignment.

### 7.5 Computational Modeling and AI‐Assisted Design

Computational modeling and AI‐assisted design have emerged as increasingly valuable tools for addressing the complex challenges associated with smart bioinks in 4D bioprinting, as highlighted in recent studies [36, 282]. Before physical printing, physics‐based simulations, ML algorithms, and multi‐scale modeling approaches can be used to predict and optimize key properties and behaviors of smart bioinks, such as printability, mechanical characteristics, dynamic 4D transformations, and cell viability. For example, FEA and ML models have been applied to predict the deformation of complex 4D structures under external stimuli, enabling the virtual screening of geometric designs and material combinations [178, 186, 205]. Molecular dynamics simulations can provide insight into polymer–polymer, polymer–nanoparticle, or supramolecular interactions within smart bioink formulations, while rheological and constitutive models can help optimize flow behavior, shear‐thinning properties, and printing parameters [283]. In addition, real‐time computational modeling integrated with in situ monitoring may support adaptive printing processes, in which feedback from the printing process is used to dynamically adjust parameters such as extrusion pressure, nozzle speed, temperature, or crosslinking conditions.

Despite these advances, current AI/ML applications in 4D printing remain concentrated mainly in acellular systems, such as shape‐memory polymers and stimuli‐responsive hydrogel actuators, where design objectives including actuation strain, response time, recovery ratio, and programmed shape change are relatively well defined, rapidly measurable, and reproducible. By contrast, AI/ML‐guided design of cell‐laden 4D bioinks and living constructs remains at an early stage. Several key limitations must be carefully considered and addressed before the full potential of AI/ML in this field can be realized.

First, cell‐laden smart bioinks involve coupled physicochemical, mechanical, and biological processes that are difficult to capture within a single predictive framework. A useful model would need to account simultaneously for bioink formulation, rheological behavior, crosslinking kinetics, printability, stimulus responsiveness, cell viability, cell–material interactions, and time‐dependent 4D transformation. In practice, however, many existing models simplify these relationships by treating the material component and the cellular component separately, or by reducing 4D behavior to a limited set of static outputs. This simplification is particularly problematic for living constructs, where biologically relevant 4D behavior extends beyond material shape change and includes dynamic cell–matrix interactions, matrix remodeling, traction‐mediated deformation, mechanotransduction, and changes in cell phenotype over days to weeks.

Second, the requirement for cytocompatibility substantially narrows the design space for cell‐laden 4D bioinks. Many stimuli and chemistries commonly used in acellular 4D printing, such as large temperature shifts, extreme pH, organic solvents, strong chemical triggers, or high‐intensity irradiation, are unsuitable for living systems. Therefore, AI/ML models trained on acellular 4D materials cannot be directly transferred to cell‐laden bioinks without accounting for cell survival, function, and long‐term tissue maturation. Moreover, living cells introduce stochastic, donor‐dependent, cell source‐dependent, and batch‐dependent variability, which increases experimental noise and complicates model training.

Third, the field remains constrained by the limited size and poor standardization of available datasets. Most training data are generated by individual laboratories using distinct materials, printers, nozzle geometries, crosslinking methods, environmental conditions, cell types, and characterization protocols. Because these datasets are rarely reported in standardized, machine‐readable formats, they are difficult to aggregate across studies. This lack of standardization also causes domain shift, whereby a model trained using data from one laboratory, printer, formulation batch, or cell source may fail to apply to another experimental setting.

Fourth, current modeling practices are often limited by insufficient methodological rigor. Data leakage can arise when replicate measurements from the same formulation, or information extracted from the full dataset during preprocessing, are shared between training and test sets, resulting in overly optimistic estimates of model performance. In addition, many models are evaluated only using internal random splits, while external validation using independent datasets and prospective validation through newly designed experiments remain uncommon. As a result, the ability of these models to generalize to unseen formulations, printers, cell types, or biological contexts remains uncertain.

Fifth, most current models provide point predictions without rigorous uncertainty quantification. This limits their utility for experimental prioritization, as users cannot easily determine whether a prediction is reliable or reflects extrapolation beyond the training domain. Approaches such as Bayesian modeling, ensemble learning, and conformal prediction could provide more informative estimates of model confidence and help guide risk‐aware experiment design. Similarly, the black‐box nature of many ML models, particularly deep‐learning architectures, limits interpretability and makes it difficult to extract mechanistic structure–property–function relationships that would be scientifically actionable.

Finally, the underreporting of negative and failed formulations remains a major obstacle. Published datasets are biased toward successful materials and optimized printing conditions, whereas failed formulations, poor printability, cytotoxic responses, and unsuccessful shape transformations are rarely reported in detail. This publication bias distorts the apparent design space and may cause ML models to overestimate performance or miss important failure boundaries.

### 7.6 Clinical Translation

The clinical translation of smart bioinks for 4D bioprinting holds promise for advancing regenerative medicine, personalized therapies, minimally invasive surgical interventions, disease modeling, and drug testing. Compared to conventional 3D‐printed constructs, 4D‐bioprinted tissues possess the unique ability to dynamically adapt to physiological environments, enabling innovative applications such as self‐fitting implants, programmable drug delivery systems, and responsive tissue scaffolds that evolve in synchrony with the healing process. From a clinical perspective, this technology holds great potential to address key challenges including vascularization in thick tissue grafts, patient‐specific organ repair, and in situ regeneration of complex anatomical structures. Although 4D bioprinting utilizing smart bioinks demonstrates significant transformative potential in biomedical applications, its clinical translation is still in its infancy, with most developments limited to preclinical studies. To date, no 4D bioprinted constructs have received regulatory approval for human use. However, extensive efforts are underway to bridge the gap between laboratory innovation and clinical implementation. The primary challenge lies in ensuring the biocompatibility of smart bioinks and printed structures, as many stimuli‐responsive materials exhibit cytotoxic effects or elicit adverse immune responses when introduced into the human body. The dynamic behavior of these smart bioinks introduces further complications, such as unpredictable shape transformations in complex biological environments and difficulties in maintaining cell viability during structural adaptations. Furthermore, preclinical research into 4D bioprinted constructs remains limited. Although promising preclinical results indicate potential applications, such as self‐fitting implants and programmable drug delivery systems, significant knowledge gaps persist regarding long‐term biosafety and the integration of printed structures with host tissues.

### 7.7 Future Perspectives

Although 4D bioprinting is still in the proof‐of‐concept stage and faces major limitations, sustained interdisciplinary efforts across biomaterials science, biology, life science, engineering, medicine, and computational modeling are underway to advance this emerging field. For example, hybrid bioinks that combine natural and synthetic polymers offer a promising solution to overcome the poor printability and/or insufficient mechanical properties of smart bioinks. By combining the biocompatibility and bioactivity of natural components (e.g., Alg, gelatin, HA, etc.) with the mechanical strength and stimuli‐responsive properties of synthetic polymers (e.g., PNIPAM, PEG, etc.), hybrid bioinks could display not only enhanced cell viability but also more accurate shape‐morphing behavior and functional transformation processes. Such bioinks could show improved printability and structural stability while enabling dynamic responsiveness. Moreover, hybrid formulations allow for tunable degradation rates and responsiveness to multiple stimuli, making them especially well‐suited for advanced biomedical applications in basic research, pharmaceuticals, tissue engineering, and precision medicine.

Due to the early development stage of 4D bioprinting technology, smart bioinks often rely on single stimuli, such as temperature, moisture, and pH, for activation. This reliance typically leads to limited control over shape transformations and reduced adaptability in complex biological environments. Single‐stimulus activation generally induces homogeneous structural changes, which are insufficient to meet the spatially heterogeneous transformations required for anatomically precise tissue reconstruction. Therefore, integrating multi‐stimuli‐responsive and advanced stimuli‐responsive mechanisms into smart bioinks is crucial to overcome these limitations. By incorporating responsiveness to multi‐stimuli, such as combinations of light, temperature, moisture, pH, ionic concentration, magnetic fields, enzymes, and electric signals, smart bioinks can achieve more precise, sequential, and spatially controlled deformations. Furthermore, advanced stimuli‐responsive features, such as biochemical cues (e.g., enzyme‐sensitive peptides) or biohybrid actuators (e.g., engineered living materials), can enhance biocompatibility and bioactivity, enabling dynamic interactions with cellular microenvironments. These interactions promote tissue maturation while preserving structural integrity. Overall, the integration of multi‐stimuli and advanced responsiveness in smart bioinks can address key challenges facing smart bioinks, including poor shape fidelity, limited biological functionality, and unpredictable in vivo behavior.

While current smart bioinks have predominantly relied on external physical or chemical stimuli for actuation, the ultimate objective of 4D bioprinting is to fabricate autonomous, intelligent constructs capable of sensing and adapting to the body's intrinsic biochemical cues. Endogenous stimuli‐responsive bioinks, which are materials that react to microenvironmental alterations in vivo, such as changes in inflammatory cytokines, disease‐specific enzymes, pH variations, and oxidative stress, represent the next frontier in this field. These bioinks would facilitate genuinely personalized, on‐demand therapeutic interventions, where the construct functions as a living sensor and actuator that continuously monitors the tissue environment and delivers precise, spatially controlled therapeutic responses. Realizing this vision will necessitate a fundamental transformation in bioink design from passive responsiveness to active sensing, computation, and adaptive behavior.

With continued advancements in 3D printing technologies, it is feasible to fabricate complex 3D structures with controlled geometries and satisfactory precision. However, the encapsulation of cells within bioinks compromises the achievable printing resolution in bioprinting. Notably, high resolution (≤50 µm) is critical for constructing intricate microarchitectures, such as vascular networks or neural interfaces, that are essential for functional bioprinted tissues. Without sufficient precision, stimuli‐responsive shape transformations may distort fine structural details, thereby compromising biological functionality. Meanwhile, improvements in printing speed are necessary to enable scalable production while preserving cell viability, as prolonged printing processes can reduce bioink stability and cell survival rates. Therefore, technological innovations in bioprinting are indispensable. For instance, advancements in light‐based bioprinting techniques, such as volumetric bioprinting and projection SLA, enable high‐resolution and high‐speed patterning of photo‐responsive bioinks, facilitating the creation of complex 4D architectures with micron‐scale accuracy. Furthermore, multi‐material bioprinting technologies allow for the simultaneous deposition of complementary bioinks, which can be employed to integrate stimuli‐responsive polymers with supportive hydrogels to simultaneously achieve structural integrity and dynamic responsiveness. Embedded bioprinting within granular gels or yield‐stress baths further improves shape fidelity for delicate smart bioinks, preventing structural collapse during the printing process and subsequent transformations.

AI is a promising and rapidly developing tool for addressing persistent challenges in smart bioink design for 4D bioprinting. For cell‐laden 4D bioprinting, the relationships between formulation, processing, structure, and function are complex, costly to map experimentally, and strongly affected by biological variability. In such cases, sample‐efficient strategies, such as active learning and Bayesian optimization, are attractive because they can use each experimental outcome to guide the selection of the next most informative formulation or processing condition. In addition, datasets from acellular 4D materials, hydrogel systems, and broader biomaterials research may provide useful priors for transfer‐learning or physics‐informed models that can be further refined using smaller, biologically relevant datasets. AI‐assisted multi‐objective optimization could help balance the competing requirements of printability, shape‐morphing fidelity, mechanical performance, degradation behaviour, and cell viability. AI‐assisted computational models may also help predict the behaviour of 4D‐printed constructs under physiologically relevant conditions, thereby reducing reliance on purely trial‐and‐error experimentation.

Despite this potential, the practical impact of AI in smart bioink design will strongly rely on the availability of high‐quality, standardized, and reusable data. Accordingly, an important priority in the near future is to establish the data infrastructure required for trustworthy model development. First, bioink and 4D‐printing datasets should be curated according to the FAIR principles (i.e., Findable, Accessible, Interoperable, and Reusable) with persistent identifiers, machine‐readable metadata, controlled vocabularies, explicit provenance, and clear licensing. Second, the community should develop minimum‐information reporting standards for bioprinting, analogous to reporting frameworks such as MIAME and MIQE, to improve reproducibility and cross‐study comparability. Such standards should include key information on bioink composition and preparation, rheological properties, printer type and settings, crosslinking and stimulation conditions, printability metrics, cell source and viability assays, and post‐printing transformation behaviour. Third, these reporting standards should be paired with standardized experimental protocols, for example, harmonized rheological and printability assays and mechanical testing aligned with existing ISO/ASTM additive‐manufacturing standards, so that measurements are directly comparable across groups. Fourth, open, version‐controlled repositories and community benchmark datasets, deposited in general‐purpose archives or dedicated bioprinting databases, would support data aggregation, model training, and fair comparison through shared benchmark tasks and challenges. Fifth, negative and failed formulations should be systematically reported to reduce publication bias and better define the true design space. Alongside these data‐centric measures, AI models should adopt rigorous external and prospective validation, uncertainty quantification, safeguards against data leakage, and interpretable modeling strategies that reveal meaningful structure–property–function relationships. Only with such standardized FAIR datasets and validation practices in place can AI evolve from a promising adjunct into a reliable enabler of clinically relevant smart bioinks and dynamic 4D‐bioprinted tissue constructs.

Furthermore, beyond structural shape morphing, 4D bioprinting provides distinctive advantages to endow printed constructs with enhanced biological functionality that develops over time. Dynamic transformations can be utilized to incorporate bioactive cues (e.g., growth factors, adhesion ligands) in a temporally controlled way, as shape alterations expose previously sequestered biochemical domains to guide cell behavior. The mechanical signals generated during shape morphing, such as variations in substrate stiffness or tensile strain, can actively regulate stem cell fate via mechanotransduction pathways, potentially steering differentiation towards desired lineages. In fact, such mechanoresponsive responses are innate to tissue morphogenesis and matrix turnover in vivo. Moreover, programmed shape changes can be engineered to modulate immune responses by, for example, increasing anti‐inflammatory agent release in response to inflammatory signals, reducing foreign body reactions and promoting host integration. As scaffolds degrade and remodel in synchronization with tissue maturation, 4D constructs can gradually transfer mechanical load to newly formed ECM, facilitating functional tissue regeneration. By progressing beyond structural mimicry towards functional programming, 4D bioprinting has the potential to create intelligent, adaptive living systems that actively guide tissue regeneration and restore physiological function.

The clinical translation of 4D bioprinted constructs hinges on tackling several industrial and regulatory challenges. First, smart bioinks should be fabricated in strict accordance with Good Manufacturing Practice (GMP) standards to guarantee batch‐to‐batch consistency, sterility, and effective quality control. The transition from laboratory‐scale synthesis to industrial production poses substantial challenges, especially for multi‐component bioinks such as those incorporating polymers, nanoparticles, and cells. Crucial considerations include maintaining reproducible rheological properties and stimuli‐responsive behavior across different batches, ensuring the uniform dispersion of components, and establishing robust quality control metrics, such as viscosity, gelation kinetics, and mechanical properties. Second, conventional sterilization methods, such as autoclaving, ethylene oxide treatment, and gamma irradiation, can cause polymer degradation, protein denaturation, or impairment of stimuli‐responsive properties, rendering them largely incompatible with smart bioinks. This necessitates the development of alternative strategies, such as sterile filtration, aseptic processing, low‐temperature plasma sterilization, and supercritical CO_2_ sterilization. Moreover, preservation technologies like lyophilization or cryopreservation are essential to prolong the shelf life and enable off‐the‐shelf availability of smart bioinks, while ensuring that the reconstituted bioinks preserve their original printability, responsiveness, and cell‐supporting properties. Third, there is currently a lack of regulatory frameworks specifically designed for 4D bioprinted constructs, as these devices occupy a regulatory gray area spanning tissue‐engineered products, combination products, and medical devices. Key considerations encompass the classification of such constructs, demonstration of safety and efficacy under dynamic in vivo conditions, establishment of standardized testing protocols for stimuli‐responsive behavior, and development of risk assessment frameworks tailored to dynamic constructs. Collaborative engagement with regulatory agencies such as the FDA will be crucial to establish clear and harmonized guidelines that facilitate clinical translation while guaranteeing product safety.

## 8 Conclusions

4D bioprinting has emerged as a revolutionary advancement over conventional 3D bioprinting, enabling the creation of dynamic, stimuli‐responsive structures that more accurately replicate the complexity of native tissues. The success of 4D bioprinting critically depends on smart bioinks, which govern the shape‐morphing behavior, functionality, and performance of the printed constructs. While recent advances in AI‐driven optimization have significantly expedited the design and development of smart bioinks, challenges such as limitations in biomaterial selection, the need for precise stimulus control, and the barriers to clinical translation remain to be addressed. Specifically, the structural, anatomical, and temporal needs of the target tissues must be fully integrated into the design of the relevant bioinks. Future research should prioritize the development of multi‐stimuli‐responsive bioinks, incorporation of real‐time monitoring systems, and the application of big data modeling to facilitate the design of smart bioinks and fabrication of 4D bioprinted constructs. Furthermore, to expand the applications of 4D bioprinting in personalized medicine, drug discovery, and disease modeling, interdisciplinary collaboration among materials scientists, engineers, and biomedical researchers will be essential. By overcoming current limitations and harnessing emerging technologies, 4D bioprinting is poised to drive transformative advancements in biomedical innovation and tailored therapeutic solutions.

It is important to note that while this review encompasses a broad range of smart materials—from acellular 4D printing systems to emerging cell‐laden bioinks—the translational readiness of these technologies varies considerably. Acellular approaches are closest to clinical translation, whereas true 4D bioprinting remains at an early stage of development. Hybrid strategies represent an intermediate development pathway that may accelerate clinical translation.

## Author Contributions

**Shangsi Chen**: writing – original draft, conceptualization, writing – review and editing, methodology, investigation. **Jiahui Lai**: conceptualization, writing – original draft, writing – review and editing, methodology, investigation. **Qiongjiao Zeng**: writing – original draft, investigation. **Liangbin Zhou**: writing – original draft, investigation. **Boguang Yang**: writing – original draft, investigation. **Bin Zhang**: writing – review and editing, formal analysis. **Min Wang**: writing – review and editing, formal analysis. **Jiajing Zhou**: writing – review and editing, formal analysis. **Kieran Lau**: writing – review and editing, formal analysis. **Khoon S. Lim**: writing – review and editing, formal analysis. **Zhong Alan Li**: writing – review and editing, conceptualization, project administration, supervision, funding acquisition. **Rocky S. Tuan**: writing – review and editing, supervision, funding acquisition, project administration.

## Conflicts of Interest

The authors declare no conflicts of interest.

## Supporting information




**Supporting File**: advs77791‐sup‐0001‐SuppMat.docx.

## Data Availability

Data will be available upon reasonable request.
