# Supplementary material for: Smart Bioinks for 4D Bioprinting: Requirements, Design, and Applications
Source: Adv Sci (Weinh). 2026 Sep 27:e77791. Online ahead of print. doi: 10.1002/advs.77791 (PMC13616272; doi:10.1002/advs.77791)
Supplement: Supplementary file 1 [file ADVS-9999-e77791-s001.docx]

*Supplementary materials*

**Smart Bioinks for 4D Bioprinting:**

**Requirements, Design, and Applications**

Shangsi Chen ^a, b, †^, Jiahui Lai ^a, b, †^, Qiongjiao Zeng ^a^, Liangbin Zhou ^a, b^,

Boguang Yang ^a, b^, Bin Zhang ^c^, Min Wang ^d^, Jiajing Zhou ^e^, Kieran Lau ^f^,

Khoon S. Lim ^f^, Zhong Alan Li ^a, b, g, h, i, j, *^, Rocky S. Tuan ^a, b, g, h, k *^

^a^	Department of Biomedical Engineering, The Chinese University of Hong Kong (CUHK), Shatin, Hong Kong, China

^b^	InnoHK Center for Neuromusculoskeletal Restorative Medicine, Hong Kong Science Park, Shatin, Hong Kong, China

^c^	Department of Mechanical and Aerospace Engineering, Brunel University London, London, UK

^d^ Department of Mechanical Engineering, The University of Hong Kong, Hong Kong, China

^e^	College of Biomass Science and Engineering, Key Laboratory of Leather Chemistry and Engineering of Ministry of Education, National Engineering Laboratory for Clean Technology of Leather Manufacture, Sichuan University, Chengdu, China

^f^	Charles Perkins Centre, and School of Medical Sciences, The University of Sydney, Camperdown, NSW, Australia

^g^	Institute for Tissue Engineering and Regenerative Medicine, CUHK

^h^	Peter Hung Pain Research Institute, CUHK

^i^	Shun Hing Institute of Advanced Engineering, CUHK

^j^	Key Laboratory of Regenerative Medicine, Ministry of Education, CUHK

^k^	School of Biomedical Sciences, CUHK

^1^ These authors contributed equally to this work.

* Corresponding authors:

Rocky S. Tuan: [tuanr@cuhk.edu.hk](mailto:tuanr@cuhk.edu.hk)

Zhong Alan Li: [alanli@cuhk.edu.](mailto:alanli@cuhk.edu.cn)hk

**Literature search methodology**

This review was carried out as a narrative review with a systematic approach to literature identification. The following databases were searched: Web of Science, PubMed, Scopus, and Google Scholar. The search timeframe spanned January 2015 to March 2026, with a specific focus on studies published after 2020, during which the concepts of 4D printing and bioprinting gained substantial momentum. Key search terms included "4D printing" and "4D bioprinting" (Fig.S1).

Inclusion criteria were as follows: (1) original research articles and reviews published in English; (2) studies on 4D printing, 4D bioprinting, smart bioinks, or stimuli-responsive biomaterials; (3) studies that provided quantitative data on printability, biocompatibility, stimuli-responsiveness, or tissue engineering applications.

Exclusion criteria were as follows: (1) studies concentrating solely on 3D printing without stimuli-responsive components; (2) studies on non-biomedical applications of 4D printing; (3) conference abstracts, patents, and non-peer-reviewed publications.

Fig.S1 The literature search methodology in this review articles.

Given the interdisciplinary nature of this review and the heterogeneity of the literature, we adopted a thematic organization instead of a systematic quantitative synthesis. Articles were chosen based on their representativeness, scientific rigor, and relevance to the key themes of the review, which include smart bioink design (printability, biocompatibility, stimuli-responsiveness), biomaterial classification (responsiveness to physical, chemical, and biological stimuli), and biomedical applications (tissue engineering, disease modeling, drug delivery). Preference was given to studies that offered mechanistic insights and quantitative characterization, or showed high translational potential. The selection process was iterative, with additional references identified via citation tracking of key articles.

Given the limited number of primary research articles on 4D bioprinting, we also incorporated 4D printing studies with high potential for adaptation to 4D bioprinting applications, while clearly indicating the distinction throughout the manuscript. This approach guarantees comprehensive coverage while preserving scientific precision.
